# Dual-path suppression of thermal and wetting-driven steel corrosion for marine structure

**DOI:** 10.1038/s41467-026-71930-x

**Published:** 2026-04-21

**Authors:** Xiantong Yan, Fan Zhang, Shirui Peng, Hanya Yan, Meng Yang, Wenhui Duan, Xiaohua Bao, Xiangsheng Chen, Hongzhi Cui

**Affiliations:** 1 State Key Laboratory of Subtropical Building and Urban Science, GuangDong Engineering Technology Research Center of Low-Carbon and Energy Efficiency in Buildings, College of Civil and Transportation Engineering, Shenzhen University, Shenzhen, China; 2 Department of Materials Science and Engineering, Southern University of Science and Technology, Shenzhen, Guangdong China; 3 Department of Civil Engineering, Monash University, Clayton, VIC Australia

**Keywords:** Materials science, Engineering

## Abstract

Steel corrosion in marine concrete structures is critically exacerbated by solar heating and seawater penetration, accelerating chloride ingress and electrochemical degradation. Existing strategies often fail to synergize efficient thermal regulation and anti-wetting functionality for costal structures protection. To address this limitation, we develop a double-effect protective bi-coating, consisting of a carbonated dicalcium silicate (C_2_S)/BaSO_4_ composite gel overlaid with a layer of hydrophobic SiO_2_ nanoparticles, designed to passively suppress thermal and chemical corrosion drivers. The carbonation-activated C_2_S forms a chemically bonded calcium-modified silicate gel matrix, ensuring robust concrete adhesion, while BaSO_4_ nanoparticles enhance solar reflectance (94.6%) and mid-infrared emittance (92.8%), enabling efficient passive cooling. Integrated with the hydrophobic overlayer for waterproofing, this protective bi-coating achieves a sub-ambient cooling (4.13 °C) under direct sunlight and excellent anti-wetting property (water contact angle of 151.3°), effectively mitigating solar thermal loading and chloride penetration. Furthermore, after 30 days of cyclic solar illumination-salt spray exposure, the protective layer can significantly reduce corrosion initiation time and current density, demonstrating double-effect protection for reinforced concrete through ion-blocking and cooling-enabled corrosion buffering. This work pioneers a passive strategy that synergizes radiative cooling, chemical compatibility, and ion-blocking functionality to extend the service life of marine infrastructure under coupled thermo-chemo degradation.

## Abstract (web summary)

Dual-functional coating with radiative cooling and superhydrophobicity protects marine structures by cooling below ambient temperature and repelling seawater. Cooling slows corrosion, while hydrophobicity prevents wetting.

## Introduction

Steel corrosion in coastal concrete infrastructure represents a persistent global challenge, precipitating catastrophic structural failures and incurring economic losses exceeding $2.5 trillion annually^1^. This degradation is synergistically accelerated by chloride ingress through seawater absorption and elevated temperatures via solar heating^2^, contrary to the durability requirements of resilient infrastructure. Conventional corrosion mitigation strategies—improving concrete compactness (via low w/c ratios, optimized aggregates)^3–5^, applying protective coatings (epoxy, galvanized, or bio-based layers)^6,7^, or developing corrosion-resistant rebars^8^ (alloyed steels) and cathodic protection systems^9^—have achieved partial success by either passively isolating steel from aggressive ions or actively modifying electrochemical reactions. Despite extensive advancements, existing corrosion mitigation methods exhibit critical limitations under harsh coastal conditions. For instance, concrete densification strategies, though effective in reducing chloride diffusivity via low-porosity designs, remain vulnerable to thermal cracking and cannot suppress temperature-enhanced corrosion kinetics; surface-applied barrier coatings (both concrete and rebar types) suffer from UV degradation, thermal shrinkage cracks, or compromised adhesion—with epoxy systems weakening concrete-rebar bonding and galvanized layers failing in high-chloride environments^10,11^. Although advanced alternatives like alloyed rebars elevate de-passivation thresholds and sacrificial anodes provide electrochemical protection^12,13^, they introduce prohibitive costs, weldability issues, or energy/maintenance dependencies^11^. Crucially, such strategies typically target singular corrosion drivers (e.g., chloride barrier formation or anodic passivation), neglecting the thermo-wetting coupling induced corrosion acceleration under marine exposure. This critical oversight calls for a paradigm shift from single-mechanism protection to a coupled temperature-mediated suppression approach.

Modifying cementitious materials to achieve superhydrophobic characteristics offers a promising corrosion protection approach by leveraging water-repelling properties to minimize concrete-corroded interfaces^14^. These modifications typically employ micro-nano textured surfaces combined with low surface energy materials to trap air pockets and prevent corrosive agents from reaching interior structures^15^. In some cases, the porous structure of superhydrophobic material can facilitate the expulsion of water droplets containing corrosive ions due to Laplace pressure^16^, further enhancing corrosion resistance. Based on the multiscale roughness of concrete substrate, additive agents like fluorocarbons^17^, silanes^18,19^, and polydimethylsiloxane^20^ etc. are commonly applied to reduce the surface energy, enhancing the water-repelling effect of whole bulk concrete^21^. However, the bulk modification is only applicable in new buildings and its effectiveness usually achieved at the expense of deteriorated mechanical strength of concrete materials^22^. By contrast, incorporating nanoparticles into coatings on new/old concrete surface can simultaneously enhance both surface roughness and low surface energy properties, mitigating the mechanical compromission and application limitation as compared to bulk modification^23^. Despite its effectiveness, the coating’s anti-wetting performance is susceptible to deterioration from cyclic evaporation-condensation of seawater under high thermal load. More importantly, this strategy solely focuses on wetting-mediated corrosion suppression in coastal concrete structures, leaving the kinetic acceleration caused by solar thermal loading unmitigated, thereby failing to resolve the thermo-wetting coupling inherent to marine corrosion.

Recent research has identified temperature regulation as a promising auxiliary mechanism for corrosion suppression, given that thermal elevation exponentially accelerates both chloride diffusion coefficients (decreases by at least 80% from 20 °C to 70 °C)^24^ and electrochemical reaction rates^4,11^. Although investigations have explored reflective surfaces^25,26^ and phase-change materials^27^ for thermal regulation of cementitious materials, these solutions are often limited to either insufficient cooling efficiency or substrate mechanical degradation. Particularly, conventional reflective pigments in cementitious substrate demonstrate limited solar reflectivity^28^, while phase-change materials are constrained by low thermal conductivity and cyclic instability^29^. In striking contrast, radiative cooling technology—enabling passive sub-ambient cooling via high solar reflectance (0.28–2.5 μm) and efficient infrared emission within the atmospheric transparency window (8–13 μm)^30^—presents untapped potential for mitigating Arrhenius-type kinetic acceleration in coastal concrete structures. As a compatible choice for concrete, cementitious radiative coolers have attracted significant interest due to a unique combination of being readily available and scalable, cost-effective, durable, and highly emissive (from infrared-active groups), with a hydraulic property that avoids energy-intensive sintering^28,31,32^. Existing research has primarily focused on tailoring the spectral characteristics of cementitious materials to match those of ideal radiative coolers through dielectric modification (using Al_2_O_3_, BaSO_4_, SiO_2,_ etc.)^31,33^, meta-surface engineering for enhanced Mie scattering^34^, or hybrid design optimization^35,36^. Among these efforts, three studies^31,34,36^ have surpassed the practical threshold of 94% solar reflectance required for meaningful daytime radiative cooling. On parallel research directions, low-carbon cementitious radiative coolers based on industrial byproducts^37,38^ and/or carbonation mineralization^39^ have been explored, leveraging both carbon reduction benefits and enhanced optical properties through either dielectric modification^40^ or kinetics modulation^39,41^. Despite these advances in understanding the chemical and physical origins of the optical properties of cementitious radiative coolers, these existing cementitious coolers primarily target building energy saving and their rational transformation into protective coatings for marine infrastructures remains unexplored. The fundamental challenge lies in simultaneously achieving high cooling performance, impermeability to chloride-laden seawater, and concrete-compatible adhesion in costal environment. Addressing these limitations requires innovative material architectures that integrate passive radiative cooling, hydrophobic barriers, and concrete-compatible bonding within a unified coating system. Consequently, no existing solution achieves the simultaneous delivery of passive sub-ambient cooling, robust chloride exclusion, and durable concrete adhesion—a technological gap rooted in incompatible material design paradigms for mitigating the temperature-wetting coupling in marine corrosion.

Herein, we propose a temperature-wetting mediated passive anti-corrosion coating by harnessing the synergistic combination of radiative cooling and superhydrophobicity within a cementitious system. This multifunctional protective coating is achieved through a rationally designed barium sulfate-modified carbonated dicalcium silicate (C_2_S) inorganic gel integrated with a hydrophobic SiO_2_ overlayer. The carbonation-activated C_2_S forms a calcium modified silica gel matrix, functioning as a cementitious binder to enhance interfacial adhesion on concrete substrates. The as-fabricated coating exhibits exceptional solar reflectance (94.6%) and mid-infrared emissivity ( ~ 0.93), yielding 4.13 °C sub-ambient temperature reduction under peak solar irradiance—thereby fundamentally suppressing corrosion thermodynamics via Arrhenius kinetics retardation. Concurrently, the integrated hierarchical SO_2_ overlayer confers superhydrophobicity (water contact angle of 151.3°), effectively inhibiting capillary-driven chloride transport. These two effects synergistically enables a multi-stage corrosion protection with the hydrophobic layer blocks initial ions ingress, while cooling retards subsequent diffusion and reaction kinetics. Our work establishes a unified paradigm for marine corrosion resistantence via bridging advances in material chemistry, thermal physics, and corrosion electrochemistry into an energy-free protective technology.

## Results

### Targeted pathway to suppress thermo-wetting coupled corrosion in marine concrete

**Fig. 1:**
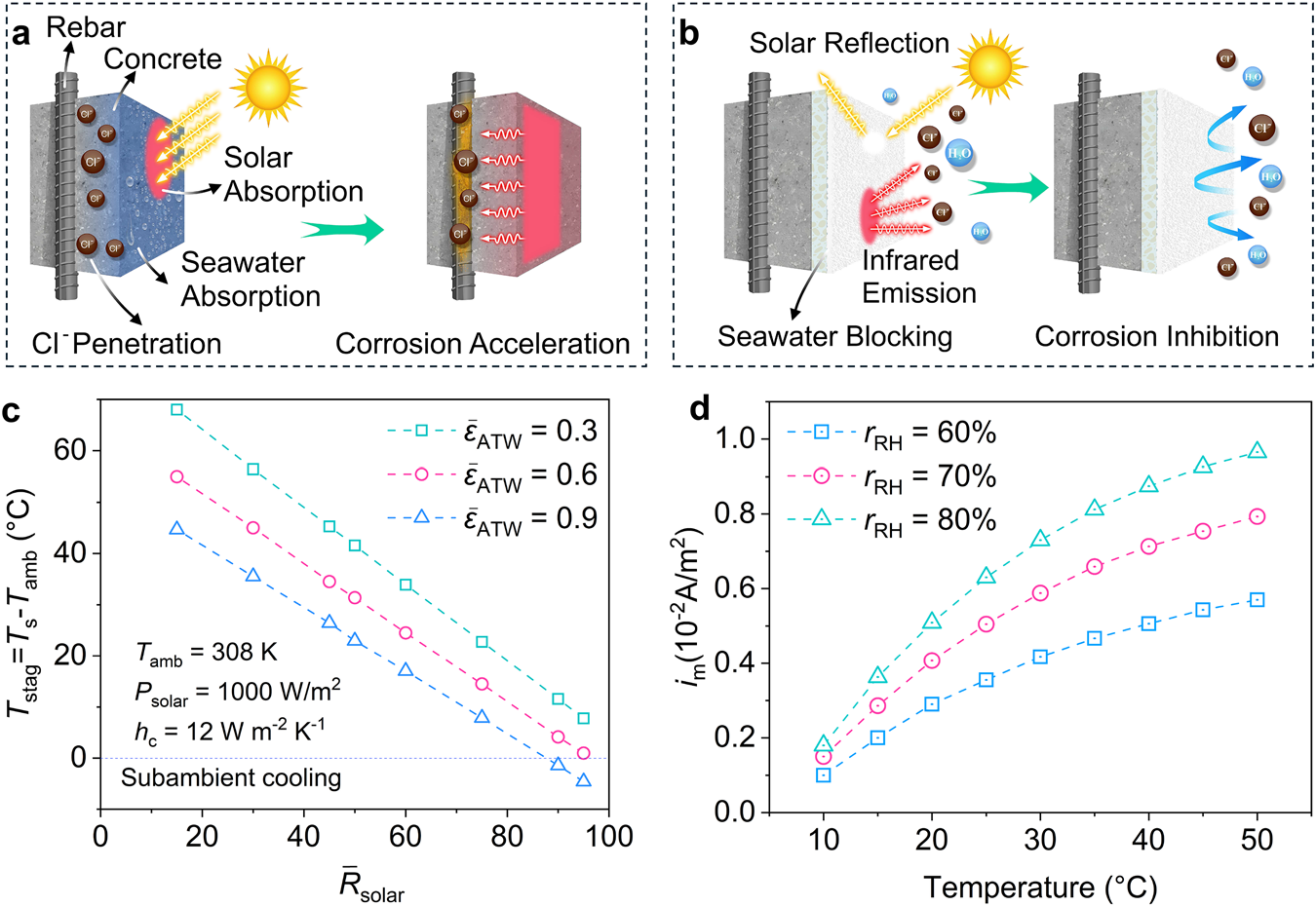
Design and model calculations of traditional reinforced concrete and double-effect bi-coating protected reinforced concrete. **a**,** b** Schematic diagram of two corrosion models. The proposed double-effect protective coating is characterized by a bilayer structure, which synergizes passive radiative cooling and hydrophobic ion-blocking simultaneously. **c** The relationship between the average solar reflectance (*R̅*_solar_) and stagnation temperature (*T*_stag_) for different average atmospheric transparency window (ATW) emissivity (*ε̅*_ATW_) in the long wave infrared range. **d** The impact of temperature (*T*_s_) and relative humidity (*r*_RH_) on the corrosion current density (*i*_m_) of steel bars in concrete structure. The dashed lines in Fig. 1c, d represent theoretical calculations based on the radiative cooling model (see Supplementary Note 1 and Eq. 1) and the corrosion kinetics model (see Eqs. 2–6), respectively. Source data are provided as a Source Data file.

Steel corrosion in coastal reinforced concrete structures is inherently governed by the synergistic interplay of temperature and humidity, which collectively accelerate chloride ingress and electrochemical degradation. Generally, concrete materials are intrinsically hydrophilic and solar-absorptive (Fig. 1a). When exposed to marine climates, capillary absorption of seawater under high relative humidity (RH > 80%) establishes continuous electrolyte pathways, facilitating chloride diffusion and localized corrosion cell formation^42^. Simultaneously, under solar irradiation, the low reflectivity of concrete surface leads to substantial heat absorption and significant temperature fluctuations (e.g., 18.9–57.4 °C diurnal cycles in a summer day of ShenZhen City, Figure S1). As a result, the internal temperature of the steel bar embedded within the concrete cover rises rapidly until reaching a high-temperature equilibrium. This elevated temperature reduces critical chloride threshold required for depassivation and improves the corrosion rates via the Arrhenius effect, severely compromising the lifespan of steel bars in coastal structures^24^. Traditional corrosion mitigation strategies, such as passive epoxy coatings or active cathodic protection, often address singular factors (e.g., chloride barrier or electrochemical passivation) but fail to account for the temperature-wetting coupling inherent to marine climates. Therefore, the key to high-performance anti-corrosion requires effective control of temperature elevation on the concrete surface and blocking the transportation of corrosive substances.

For this purpose, we designed a temperature-wetting mediated passive anti-corrosion coating (T-PAC) that synergizes radiative cooling and hydrophobic barriers for marine structures protection. In our design, the anti-corrosion coating comprises a hydrophobic layer deposited on a carbonated dicalcium silicate (C_2_S)/BaSO_4_ gel matrix (Fig. 1b). The hydrophobic layer with excellent water repellence is capable of suppressing capillary absorption of seawater in marine environment, thus preventing the ingression of corrosive medium. Even though the corrosive medium penetrates into the coating as time goes by, the radiative cooling layer with improved solar reflectivity and mid-infrared emissivity has the potential to reduce the surface temperature of marine structures by reflecting solar irradiation and emitting infrared radiation, decelerating the transport of corrosive medium and reducing corrosion rate. To characterize the thermal response of the anti-corrosion coating protected concrete structures during direct solar irradiation, a steady state heat transfer model incorporating radiative cooling is developed. Considering the ambient air and atmosphere in the marine environment, the cooling power of the radiative cooling coating applied to the underneath concrete structures can be calculated using the following Eq. (1):

1 $${P}_{{\rm{coating}}}={P}_{{\rm{rad}}}-{P}_{{\rm{amb}}}-{P}_{{\rm{sun}}}-{P}_{{\rm{non}}}-{P}_{\rm{non}\,-{{\rm{radiative}}}}$$

where *P*_coating_, *P*_rad_, *P*_amb_, *P*_sun_, and *P*_non−radiative_ represent the net cooling power of the coating, radiative cooling power, ambient radiation absorption power, solar absorption power, and non-radiative power, respectively. Detailed calculations of these powers are available in Supplementary Note 1.

During the heat transfer equilibrium, heat gain from the solar irradiation elevates the temperature of the concrete-steel system, which significantly accelerates the transport of chlorides to the steel surface, thereby promoting earlier depassivation and corrosion initiation^24^. This thermal activation phenomenon is further amplified by temperature-wetting coupling as high RH ( > 60%) sustains pore solution electrolytic conductivity and lowers the corrosion potential. Based on Butler-Volmer kinetics^43^, the relationship between temperature (*T*) and activation overpotential *η*_a_ can be expressed by the following Eq. (2):

2 $${\eta }_{{\rm{a}}}=({RT}/{\alpha n}_{{\rm{e}}}F)\cdot {\mathrm{ln}}\left\{\right.{i}_{{\rm{n}}}/{2i}_{0}+{[{({i}_{{\rm{n}}}/{2i}_{0})}^{2}+1]}^{1/2}$$

where *i*_n_ is net current density; *i*_0_ is exchange current density; *R* is universal gas constant; *F* is Faraday’s constant; *α* is transfer coefficient; *n*_e_ is number of electrons transferred in reaction. From Eqs. (1–2), the steel bar reinforced concrete structure with higher solar reflectivity and infrared emissivity enables lower surface temperatures that surpass the initiation of corrosion reaction.

In the corrosion process, the corrosion rate of the steel bar can be described by:

3 $${i}_{{\rm{corr}}}=(1/{L}_{{\rm{a}}}) \int _{0}^{{L}_{a}}{i}_{a}{dx}$$

where *L*_a_ is the length of the anode area and *i*_a_ is the corrosion current density of the anode. For the steel bar reinforced concrete system, *i*_a_ can be expressed as:

4 $${i}_{{\rm{a}}}=(-1/\rho )\cdot (\partial E/\partial n)$$

where *E* is the corrosion potential, *n* is the direction normal to the bar surface; *ρ* is concrete resistivity (Ω·m) defined by ref. ^44^:

5 $$\rho={\rho }_{0}\cdot {({t}_{{\rm{h}}}/{t}_{0})}^{{N}^{{\rm{a}}}}\cdot {K}_{{\rm{t}}}\cdot {K}_{{\rm{c}}}\cdot {K}_{{\rm{T}}}\cdot {K}_{{\rm{r}}}\cdot {K}_{{\rm{Cl}}}\cdot {K}_{{\rm{w}}/{\rm{c}}}$$

where *ρ*_0_ is reference electrical resistivity (Ω·m); *t*_0_ is curing time (d); *t*_h_ is hydration time (d); *N*_a_ is age factor; *K*_t_, *K*_c_, *K*_T_, *K*_Cl_, and *K*_w/c_ are influential factors for test method, curing method, temperature, chloride content, and water-to-cement ratio (*R*_W/C_), respectively; *K*_r_ is influential factor of relative humidity (*r*_RH_), which can be described as

6 $${K}_{{\rm{r}}}={[-1.3059{({r}_{{\rm{RH}}}/100)}^{2}+3.605({r}_{{\rm{RH}}}/100){\rm{\hbox{--}}}1.327]}^{-1};{\rm{for}}\,50\%\le {r}_{{\rm{RH}}}\le 100\%$$

Based on the above outlined physical models, the cooling function of the anti-corrosion coating was firstly analyzed by calculating its stagnation temperature (*T*_stag_ = *T*_surface_ – *T*_ambient_), defined as the maximum temperature difference between concrete surface and ambient air at *P*_net_ = 0, across different solar reflectance under varying thermal emissivity (Supplementary Note 2). As shown in Fig. 1c, the coating protected concrete surface with a high solar reflectance exhibits a much lower surface stagnation temperature as compared to that of unprotected concrete surface with low solar reflectance. Based on Eq. 1, which incorporates radiative and non-radiative heat transfer, it is calculated that, at a low atmospheric transparency window (ATW) emissivity of 0.3 in the long wave infrared range, increasing the solar reflectance of the concrete surface leads to remarkable reduction in *T*_stag_ from ~68 °C to 15 °C under a solar power of 1000 W/m^2^. At higher ATW emissivity of 0.6, the significant decreasing trend maintained as the solar reflectance increases, indicating the great potential of cooling coating in reducing the surface temperature of concrete structures. However, it is also found that enhancing solar reflectance can yield a higher cooling capacity than improving ATW emissivity by the same amount. We also note that efficient cooling performance characterized by *T*_stag_ < 0 under direct solar irradiation, known as subambient daytime radiative cooling, only achieved when both solar reflectance and ATW emittance surpass 90%. Since the solar reflectance and ATW emissivity of traditional concrete materials are respectively around 30% and 0.9 (Figure S2), the key to efficient thermo-wetting mediated anti-corrosion performance relies on effective rejecting of solar radiation on concrete surface and suppressing corrosive ion transportation within the coating. Based on Eqs. (2–6), the effect of thermo-wetting coupling on the corrosion rate of steel bar in concrete was evaluated. Figure 1d shows that elevated temperatures accelerate rebar corrosion and increased relative humidity further enhances this corrosion acceleration phenomenon. Particularly, combining 45 °C and 85% RH conditions improves the corrosion rate by ~100% compared to 25 °C/60% RH scenarios. These findings underscore the limitations of conventional single-factor corrosion models and justify the development of multifunctional coatings capable of simultaneously regulating thermal and wetting environments-a critical gap addressed by our radiative cooling/hydrophobic bi-coating design.

### Synthesis and characterization of the dual-protective coating

In developing the radiative cooling-superhydrophobic coating for marine structure protection, material selection prioritizes environmental durability, interfacial compatibility, optical efficiency, and hydrophobicity. Guided by the above design principles, we select gamma-dicalcium silicate (*γ*-C_2_S) as a carbonatable binder due to its inorganic nature against environmental aging and unique carbonation activation capability. Compared to C_3_S, C_2_S possess slower hydration rate and higher carbonation reactivity, allowing for better carbonation control^45^. Upon CO_2_ exposure, C_2_S transforms into calcium carbonates (CaCO_3_) and calcium-modified silicate gel, firmly integrating with concrete substrates via chemical bond^46^, resolving interfacial-mismatch-induced delamination issues in conventional coatings like epoxy. In addition, unlike the most widely used ordinary Portland cement (OPC), C_2_S has minimal content of Fe_2_O_3_ (Table S2) to eliminate Fe-induced solar absorption risks, while maintaining 34.3% SiO_2_ to impart intrinsic high emissivity due to Si-O vibrational modes in the atmospheric window (ATW, 8–13 μm, Figure S2). Further, to avoid seawater absorption resulting from the intrinsically hydrophilic nature of C_2_S and its carbonation products, a layer of hydrophobic SiO_2_ nanoparticles (NPs) is applied to enhance the anti-wetting properties of the coating.

To effectively minimize heat generation from solar irradiation and reduce surface temperature for corrosion inhibition, the coating should exhibit both high solar reflectivity and ATW emissivity for heat dissipation. Generally, high solar reflectivity is achieved via multiple scattering of sunlight, which can be realized by incorporating pores or dielectric particles with different refractive index into a binder matrix to create scattering interfaces^47^. However, increasing porosity in cementitious coating will cause stress concentration, thus deteriorating mechanical robustness. To mitigate this issue, introducing micro/nano-scale dielectric particles with high refractive index and wide bandgap into binder matrix present to be an appropriate strategy for simultaneously optimizing solar scattering and mechanical robustness^31^. In line with this concept, barium sulfate nanoparticles (BaSO_4_ NPs, average particle size of 480 nm, Figure S3) are selected for the optical functional modification. On the one hand, BaSO_4_ NPs possess a negligible extinction coefficient (*k*) across the solar spectrum (0.28–2.5 μm) and a wide bandgap ( ~ 7.6 eV) that is greater than the upper energy limit of solar photon ( ~ 4.13 eV)^48^, ensuring negligible solar absorption ( < 5%). Equally important, the high refractive index (*n* = 1.64) of BaSO_4_ NPs contributes to creating more scattering interfaces within the coating, thereby enhancing solar reflectivity. Moreover, phonon resonance peaks within 8–13 μm induced by the SO_4_^2−^ of BaSO_4_ NPs enhance mid-infrared emission, further promoting radiative heat dissipation^49^. In this study, the adopted BaSO_4_ NPs has a broad size distribution that is comparable to the wavelength range of sunlight (Figure S3), which enables broadband reflection in solar spectrum (Figure S4). These ideal electromagnetic properties hold great potential for sunlight scattering and infrared radiation, which is convinced to reducing the temperature of concrete surface under direct solar irradiance^50^.

**Fig. 2:**
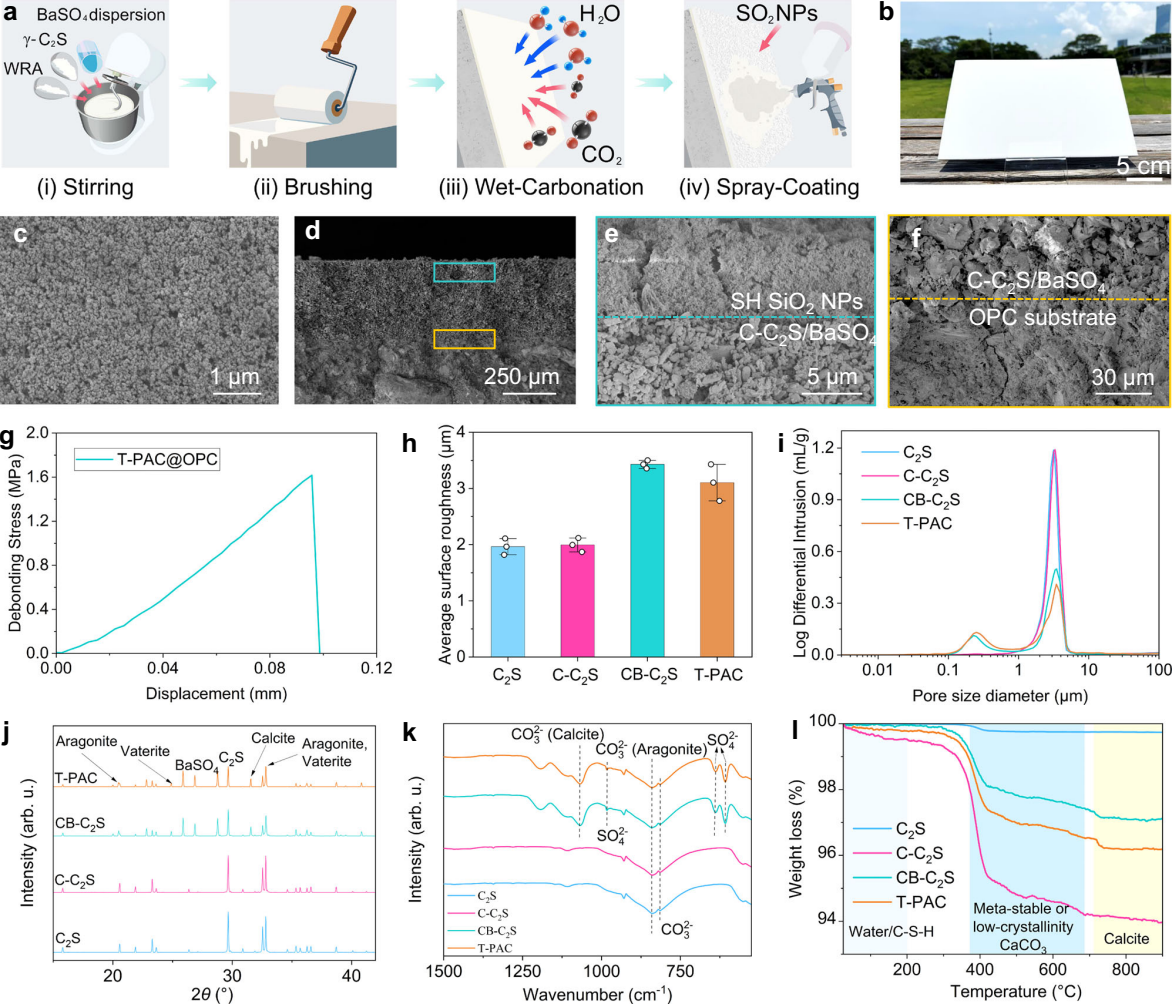
Fabrication and characterization of the double-effect protective bi-coating. **a** Schematic diagram showing the fabrication processes of the temperature-wetting mediated passive anti-corrosion coating (T-PAC). **b** Optical photograph of the T-PAC deposited on an ordinary Portland cement (OPC) substrate. **c** Top-view scanning electron microscopy (SEM) image of the T-PAC surface. **d**–**f** Cross-sectional SEM image showing the interfaces between the superhydrophobic overlayer, radiative cooling layer and the OPC substrate. **g** Interfacial bonding strength measurement conducted between T-PAC and OPC substrate. **h** Average surface roughness of the C_2_S-derived coatings tested by 3D nano-profiler. **i** Pore size distribution measured by mercury intrusion porosimetry (MIP). **j** X-ray diffraction (XRD) patterns of the C_2_S-derived coatings. **k** Fourier transform infrared spectrometer (FT-IR) spectra of the C_2_S-derived coatings. **l** Thermogravimetry analysis (TGA) curves of the C_2_S-derived coatings. The light blue, blue, and yellow shadings represent the decomposition temperature range of water/C-S-H, meta-stable/low-crystallinity CaCO_3_, and calcite, respectively. The error bars in Fig. 2h represent the standard deviations from three parallel measurements. Source data are provided as a Source Data file.

With rationally selected components, the inorganic superhydrophobic-radiative cooling coating, designed for corrosion protection of coastal structures, is fabricated through a systematic five-step process (Fig. 2a). Initially, BaSO_4_ NPs are dispersed in deionized water via ultrasonic treatment in a water bath for 15 min to ensure homogeneous dispersion. Simultaneously, C_2_S powders and water reducing agent are added to the BaSO_4_ NPs suspension in predetermined proportions and thoroughly mixed to ensure thorough contact between BaSO_4_ NPs, C_2_S particles, and water reducing agent. Then, the resulting slurry is evenly brushed onto a concrete substrate to form a uniform coating layer. The coating thickness can be tailored in an on-demand manner by applying varied amounts of fresh slurry on the concrete surface. After 24 h curing at ambient conditions, the coating undergoes a carbonation treatment in controlled environmental conditions, specifically 20% CO_2_ concentration and 70% relative humidity, for a specified duration. This carbonation reaction transforms the paste into a hardened coating, facilitating the formation of a dense cementitious matrix. Ultimately, a layer of superhydrophobic SiO_2_ NPs was spray-coated on the surface of radiative cooling layer to form a bi-coating structure. To demonstrate the advantages of T-PAC in corrosive medium blocking and radiative cooling-induced corrosion inhibition, three control coatings were fabricated under identical processing protocols: (i) Control 1 (C_2_S), comprising pure C_2_S and deionized water; (ii) Control 2 (C-C_2_S), formulated with carbonated C_2_S and deionized water while lacking BaSO_4_ NPs and superhydrophobic SiO_2_ layer; and (iii) Control 3 (CB-C_2_S), a radiative cooling coating prepared from carbonated C_2_S, deionized water, and BaSO_4_ NPs in the absence of superhydrophobic SiO_2_ layer. A preliminary estimate confirms the T-PAC's competitive cost of ~$2.71/m^2^ (Supplementary Note 3) compared to commercial alternatives like epoxy and silane treatments. Moreover, the carbonation activation of C_2_S is a carbon-negative process, offsetting the high carbon footprint associated with conventional organic coatings. These two merits in combination with the straightforward preparation process make the T-PAC coating highly promising for large-scale applications in coastal infrastructure.

The as-fabricated T-PAC on an OPC substrate (Fig. 2b) displays a pure white appearance conducive to sunlight reflection. Top-view SEM microstructural observation (Fig. 2c) reveals a flat morphology of T-PAC coating, featuring randomly distributed micro-nanovoids with numerous nanosized SiO_2_ spheres forming a hierarchically porous structure through random stacking, which can facilitate sunlight scattering and air entrapment. This ordered nanosphere-assembled architecture provides effective spherical structures, contrasting markedly with the irregular columnar particle-assembled amorphous morphologies observed in C_2_S, C-C_2_S, and CB-C_2_S coated specimens (Figure S5). Cross-sectional examination (Fig. 2d) discloses a bi-layered coating adhered to the cementitious substrate. Higher magnification imaging (Fig. 2e) demonstrates a sharp interfacial transition between densely packed SiO_2_ nanospheres and C_2_S/BaSO_4_ composites. In contrast, seamless interfacial fusion characterized by a gradual transition occurs between the carbonated C_2_S/BaSO_4_ layer and OPC substrate (Fig. 2f). This integrated interface yields robust coating-substrate adhesion (1.61 MPa, Fig. 2g, S6), exceeding conventional coating bonding strengths (0.4–0.8 MPa)^51^ and effectively mitigating interfacial-mismatch-induced delamination issues in conventional coatings. Quantitative 3D profiler and Mercury intrusion porosimetry (MIP) analyses across the coated samples further elucidate microstructural variations: pristine C_2_S coatings exhibited microscale surface roughness and is moderately enhanced by carbonation (Fig. 2h, S7), while synergistic carbonation-BaSO_4_ NP incorporation coarsened surface roughness relative to singular carbonation modifications. Conversely, additional SiO_2_ NP deposition diminished the topographic irregularities. MIP data (Fig. 2i) indicate that C_2_S carbonation reduces nanoporosity, whereas subsequent BaSO_4_ NP addition or nano-SiO_2_ deposition exerts counterproductive effects due to pore-filling. This hierarchically porous structure, crucial for radiative cooling, simultaneously functions as an effective barrier against capillary absorption of chloride-laden seawater for the bilayer coated sample.

To elucidate phase evolution mediated by BaSO_4_ NPs incorporation, carbonation, and hydrophobic modification, we conducted multi-modal characterization. Crystalline phase examination by X-ray diffraction (XRD) analysis (Fig. 2j) revealed that both pristine C_2_S and carbonated C_2_S coatings exhibited analogous diffraction patterns dominated by orthorhombic *γ*-C_2_S and metastable aragonite and vaterite polymorphs. Upon introducing BaSO_4_ NPs, orthorhombic barite (SO_4_²⁻) became the predominant crystalline phase in BaSO_4_-modified C-C_2_S composites. Concomitantly, this change is accompanied by the emergence of sharp diffraction peaks corresponding to calcite and vaterite polymorphs. However, further engineering the BaSO_4_ NPs modified C-C_2_S composites with hydrophobic overlayer induced no detectable phase alterations via XRD. This confirms that BaSO_4_ NPs exclusively drive the aragonite-to-calcite polymorphic transition, corroborated by Fourier transform infrared spectrometer (FT-IR) spectral shifts (Fig. 2k): while pristine and carbonated C_2_S samples exhibited characteristic aragonite bands, BaSO_4_-containing composites (CB-C_2_S and T-PAC) manifested distinct calcite signatures–the thermodynamically stable CaCO_3_ polymorph. Quantitative thermogravimetry analysis (TGA, Fig. 2l) further substantiated these findings: C_2_S sample shows negligible mass loss within calcite decomposition regimes ( > 780 °C), whereas carbonated C_2_S decomposed predominantly below this threshold. In contrast, all BaSO_4_-doped specimens displayed additional mass loss at 780–900 °C. These observations collectively demonstrate the BaSO_4_-enhanced crystallinity stabilization during carbonation of C_2_S, which convinced to enhanced mechanical robustness^52^ and solar reflectivity^53^.

### Anti-wetting and chloride ion blocking of the dual-protective coating

**Fig. 3:**
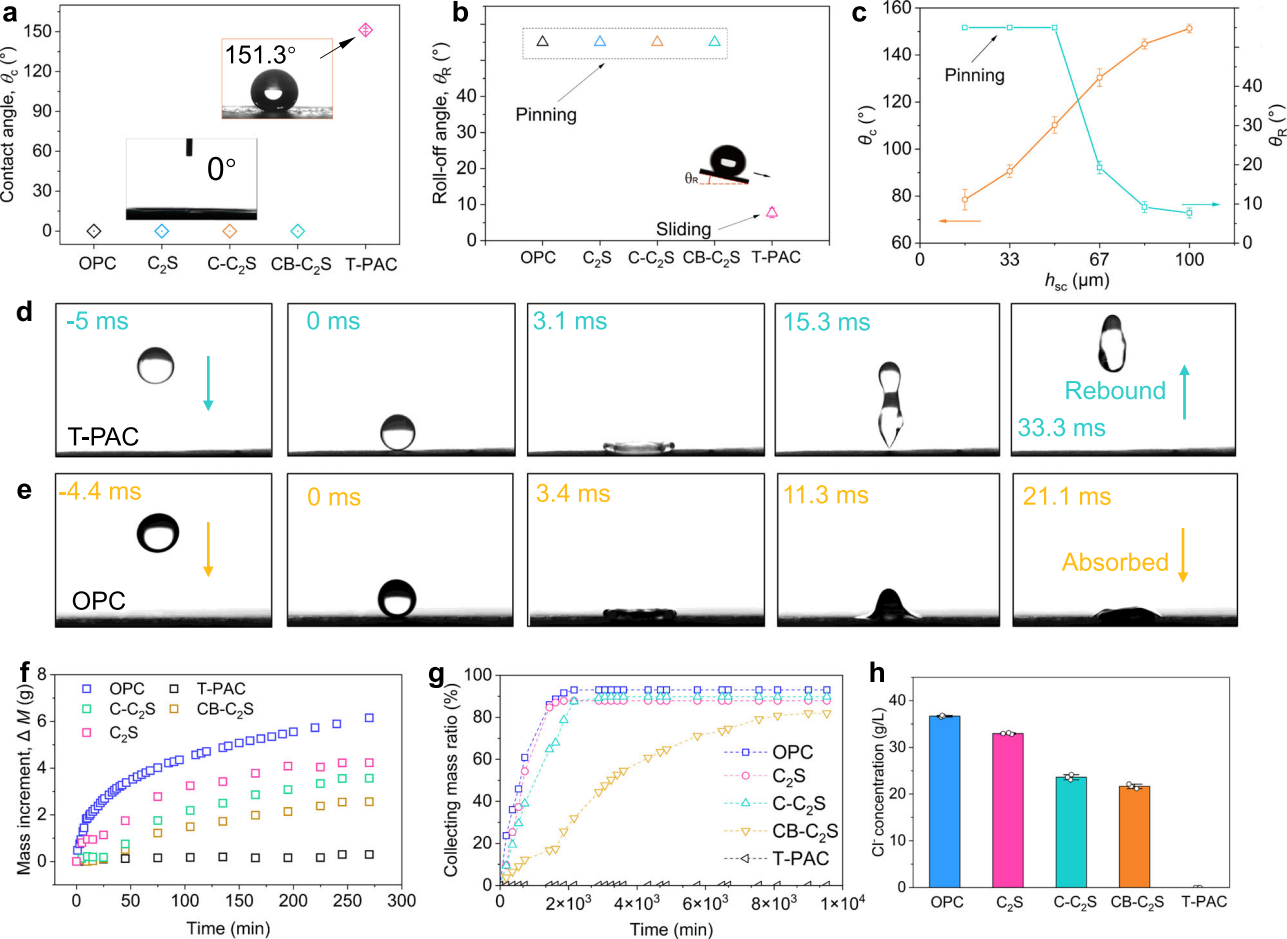
Hydrophobicity and chloride ion blocking of the coatings. **a** Static water contact angles of the C_2_S-derived coatings. **b** Dynamic droplet roll-off angles of the C_2_S-derived coatings. **c** Anti-wetting properties of the T-PAC in response to varied overlayer thicknesses. **d** High-speed images showing the complete droplet bouncing on T-PAC surface. **e** High-speed images showing the complete droplet absorption by an OPC substrate. **f** Measurement of mass increment for the C_2_S-derived coatings under high humidity (RH ≈ 100%) and elevated temperature (35 °C). **g** Collected mass ratio as a function of time for varied C_2_S-derived coatings. **h** Cl^−^ concentration in the collected solutions for varied C_2_S-derived coatings. The error bars in Fig. 3**a**–**c**, and **h** represent the standard deviation, calculated based on three replicate experiments. Source data are provided as a Source Data file.

In coastal environments, tiny seawater drops produced by splashing waves contain corrosive ions, which can penetrate into the concrete structures, compromising the protective effectiveness of concrete layer and inducing rebar corrosion. Accordingly, anti-wetting properties of the coating become the key factor governing the ingression of corrosive mediums into concrete structures through water penetration. To compare the anti-wetting properties of bare OPC and coated OPC samples, we first assessed the static water repellence of the coatings by 5 μL droplet contact angle measurements. As shown in Fig. 3a, the pristine OPC substrate can be rapidly diffused and absorbed by the deposited droplet, exhibiting superhydrophilic property with a static contact angle of 0°. This unfavorable wetting behavior is attributed to inherent hydrophilicity of OPC and its porous structure that promotes H_2_O molecules absorption. Likewise, C_2_S, C-C_2_S and CB-C_2_S coated OPC specimens maintained their inherent hydrophilicity as evidenced by their identical contact angles of 0°, despite carbonation treatment densifies substrate microstructures. Strikingly, benefitting from a hydrophobic overlayer, the T-PAC coating retained a nearly spherical profile of the deposited droplet, exhibiting excellent anti-wetting property with a static contact angle of 151.3°. Such an exceptional anti-wetting property suggests significant potential in impeding the ingression of corrosive mediums into concrete structures via water absorption.

Considering the dynamic feature of splashing drops, we then characterized the dynamic anti-wetting properties of coatings via 5 μL water droplet roll-off angle measurements on inclined surfaces. As depicted in Figs. S8 and 3b, the coating samples without hydrophobic overlayer undergo complete absorption upon droplet contact, failing to shed droplets gravitationally even at inclinations of 90°, exhibiting pronounced pinning effects indicative of strong interfacial adhesion. Conversely, the hydrophobically optimized T-PAC coating enables gravity-driven droplet detachment at angles below 5°. This efficient droplet shedding stems from synergistic low surface energy and hierarchical structures, which imparts a significant reduction in droplet/coating contact area, thereby drastically diminishing interfacial adhesion. We further note that the anti-wetting properties of the T-PAC coating are tailorable through hydrophobic layer thickness modulation (Fig. 3c). Additionally, the low interfacial adhesion is further revealed when the T-PAC coating is vertically impacted by a falling water droplet with a *Weber* number of 86.7. Figure 3d demonstrates complete droplet rebound within 40 ms after rapid droplet spreading on the T-PAC coating surface. By contrast, on an OPC surface without hydrophobic overlayer, the impacting water droplets directly spread and rapidly absorbed in the absence of rebounding process (Fig. 3e). The high affinity between OPC and water droplets underscores the critical role of low interfacial adhesion and dynamic water repellence in marine anti-corrosion design.

Having characterized the coatings’ wetting properties under individual droplet and ambient conditions, we further evaluated their performance in coastal-simulated complex environments involving multiple droplets, high humidity (RH ≈ 100%), and elevated temperature (35 °C) using 3.5 wt.% NaCl solution (Figure S9). Dynamic seawater droplet repellency in coastal-simulated complex environments was quantified via time-dependent mass increment measurements. Figure 3f reveals significant seawater absorption in unmodified samples over time, while the mass of hydrophobically optimized T-PAC rarely changes. Specifically, the fastest mass increment is observed in OPC sample due to intrinsic porous microstructure with polar groups facilitating capillary absorption of seawater. When C_2_S was coated on OPC surface, the mass increment significantly decreased since denser microstructures reduced seawater transport paths, thus inhibiting seawater absorption. It is also observed that accelerated carbonation further reduces the mass increment of the C_2_S coated sample as the carbonation product of C_2_S can fill pores in the matrix, leading to reduced permeability. This permeability-dominated water absorption is also reflected in carbonated C_2_S dosed with BaSO_4_ NPs, which exhibited smaller mass increment versus carbonated C_2_S alone due to reduced porosity rendered by BaSO_4_ NPs^39^. In striking contrast to the above unmodified coating samples, the T-PAC demonstrated minimal mass increase due to exceptional hydrophobicity that resists water spread and penetration, underscoring cooperative chemical-structural modifications’ significance in blocking the penetration of corrosive medium ladened seawater.

Except for inhibiting seawater absorption, the coatings’ ability to block the ingression of corrosive ions is another key factor affecting steel corrosion in concrete structures. In this study, the corrosive ion-blocking capabilities of different C_2_S-derived coatings were evaluated by filtering seawater through porous films coated with various C_2_S derived coatings (Figure S10). The amount of filtered solutions and the concentration of Cl^−^ in the filtered solutions were monitored over time. As shown in Fig. 3g, h, the pristine OPC sample recorded the greatest increase in the amount of collected solutions and highest Cl^−^ concentration as a result of high permeability. In contrast, coating OPC with a layer of C_2_S slightly reduced the amount of filtered solutions and Cl^−^ concentration, demonstrating that the transport of corrosive medium remains largely dominated by water permeability. This permeability-dominated Cl^−^ blocking effect is further confirmed in hydrophobically optimized T-PAC coating, which hardly allows any seawater to pass through, giving rise to the greatest Cl^−^ blocking capability. Notably, the Cl^−^ blocking effect can be amplified by additional carbonation treatment, as evidenced by a substantial reduction in Cl⁻ concentration without altering the amount of filtered solution compared to C_2_S-coated OPC. This indicates an additional Cl^−^ blocking mechanism driven by C_2_S carbonation. Furthermore, the incorporation of BaSO_4_ NPs into the carbonated C_2_S matrix leads to a notable decrease in both the Cl^−^ concentration and the volume of filtered solution, highlighting the synergistic effect of permeability-dominated and carbonation-dominated Cl^−^ blocking. These findings suggest that in the permeability-dominated regime, the coating’s Cl^−^ blocking capability is primarily influenced by surface wettability, while the carbonation of C_2_S provides an additional barrier to Cl^−^ diffusion when hydrophobic performance deteriorates. This dual mechanism stands in sharp contrast to traditional single-function coatings, which fail to simultaneously block seawater ingress and resist Cl^−^ penetration.

### Spectral characteristics and thermal regulation of the dual-protective coating

**Fig. 4:**
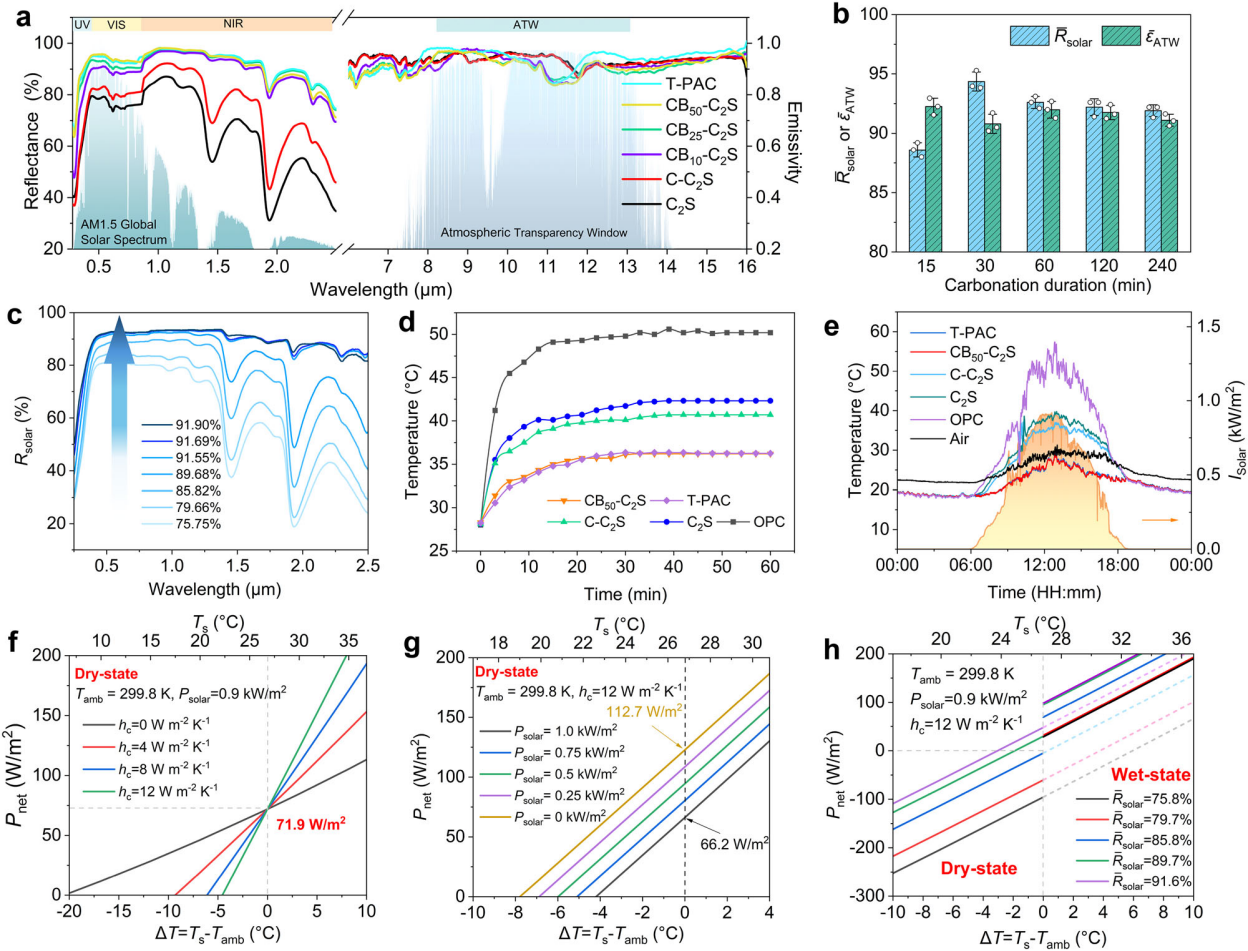
Spectrum characteristics and passive thermal regulation of the C_2_S-derived coatings. **a** Reflectance and emittance spectra of C_2_S-derived coatings subjected to different treatments and BaSO_4_ NPs dosages in the 0.28–16 μm range. **b** Average solar reflectance (*R̅*_solar_) and average atmospheric transparency window (ATW) emittance (*ε̅*_ATW_) of the T-PAC in response to varied carbonation durations. The error bars in Fig. 4b represents the standard deviation, calculated based on three replicate experiments. **c** *R̅*_solar_ variation of the water-wetted T-PAC when losing superhydrophobic properties. **d** Indoor static thermal response of the C_2_S-derived coatings as a function of time. **e**. Outdoor dynamic cooling performance of the C_2_S-derived coatings. **f** Theoretical calculations of net cooling power (*P*_net_) for T-PAC under varied non-radiative heat transfer coefficient (*h*_c_). The horizontal and vertical dash lines represent the net cooling power of 71.9 W/m^2^ and Δ*T* = 0 °C, respectively. **g** Theoretical calculations of *P*_net_ for T-PAC under varied solar radiation power (*P*_solar_). The vertical dash line represents the maximum cooling power at Δ*T* = 0 °C. **h** T**h**eoretical calculations of *P*_net_ for water-wetted T-PAC considering evaporative heat transfer. The calculations of *P*_net_ in Fig. 4f–h were conducted at an ambient temperature (*T*_amb_) of 299.8 K. The horizontal and vertical dashed lines mark the conditions of net cooling power = 0 W/m^2^ and Δ*T* = 0 °C, respectively, while the slanted dashed line illustrates the relationship between net cooling power and Δ*T* in the absence of evaporative heat dissipation. Source data are provided as a Source Data file.

The corrosion of rebar in coastal concrete structures is highly sensitive to elevated temperatures caused by solar radiation (1000 W/m^2^). To mitigate solar-heating induced corrosion acceleration, it is essential to optimize the hemispherical solar reflective and middle infrared emissive properties of the coating samples. Figure 4a shows the spectral reflectance curves of the coating samples across the ultraviolet−visible-near-infrared spectrum (0.28 − 2.5 μm). In this region, the pristine C_2_S sample exhibits an average solar reflectance (*R̅*_solar_) of 76.14% with two discernable absorption peaks located at ∼1.43 and ∼1.95 μm, attributed to O − H vibrations from molecular H_2_O. Such a relatively low *R̅*_solar_ value would generate significant heat gain from solar radiation, causing pronounced temperature elevation. In comparison, the carbonated C_2_S sample delivers an enhanced *R̅*_solar_ of 81.44%, with the improvement primarily observed in the visible-near-infrared spectrum, while the ultraviolet reflectance remains unchanged, inferring component changes induced by carbonation treatment. Further enhancement is achieved by incorporating 10 wt.% BaSO_4_ NPs into the carbonated C_2_S, raising the *R̅*_solar_ to 90.36%, exceeding the theoretical threshold (90%) for subambient daytime radiative cooling. Additionally, increasing the BaSO_4_ content to 25 wt.% and 50 wt.% further boosts the *R̅*_solar_ to 92.86% and 94.35%, respectively, due to the increased scattering interfaces. These high *R̅*_solar_ values are attributed to the synergistic effect of BaSO_4_’s high refractive index and wide bandgap, which enhance sunlight scattering and suppress solar absorption, inferring great potential in effectively minimizing temperature elevation under solar irradiation. By contrast, coating a layer of superhydrophobic SiO_2_ NPs has negligible influence on the *R̅*_solar_, probably due to the low refractive index contrast between nano-SiO_2_ and ambient air. Importantly, the average infrared emittance (*ε̅*_ATW_) in the 8–13 µm range remains consistent, fluctuating within a narrow range between 90.2 − 92.35% across all C_2_S derived coating samples. These findings indicate that the infrared emissive properties of the C_2_S-based coatings are insensitive to components variation induced by carbonation or BaSO_4_ NPs incorporation.

The hemispherical solar reflective and middle infrared emissive properties were also investigated for coatings of different thicknesses and carbonation durations. Under fixed BaSO_4_ NPs fraction and carbonation duration, the *R̅*_solar_ measured from different coatings shows an increasing trend with increased coating thickness (*h*_t_, Figure S11), owing to enhanced backscattering. However, the infrared emissive properties in the 8–13 µm range remains nearly constant across different thicknesses. This is primarily due to the ATW extinction intensity of the silicate chain (Si−O−Si) in OPC substrate is comparable to that in C_2_S, CaCO_3_, and BaSO_4_ NPs. The reflectance and emittance of coating samples treated with different carbonation durations are presented in Fig. 4b. It is observed that, the *R̅*_solar_ of the C_2_S-based composite coatings in response to varied carbonation durations shows a turning point occurring at 30 min. Specifically, an increasing trend is observed when the duration is shorter than 30 min, while a slight decrease is obtained with further extended carbonation duration, probably due to the partial screening of carbonation products on BaSO_4_ NPs^19^. In analogy to middle infrared emissive properties under varied thickness, the measured *ε̅*_ATW_ values from coating samples in the 8–13 µm range is irrelevant to carbonation durations. This can be understood because silica gel and CaCO_3_ generated by carbonation of C_2_S are intensively emissive in the ATW, thus partially covering the initial composite surface with carbonation products exerts limited impact on the infrared emissive properties. We also note that, even if the coating is wet by seawater when the hydrophobic layer lost efficacy over time, the solar reflective properties only experience a slight decrease and can restore to a *R̅*_solar_ of 91.6% within 60 mins in ambient conditions (Fig. 4c), whereas the middle infrared emissive properties remain almost constant (Figure S12), suggesting robust radiative-optical properties in coastal environments.

The excellent radiative-optical properties of T-PAC can be maintained across diverse climates and service conditions, owing to its all-inorganic composition, rational design, and functional synergy. To examine its robustness, we challenged the T-PAC by mechanical abrasion, corrosive solutions, accelerated UV aging, outdoor weathering, and submerged biofouling. Particularly, when cyclically abraded with sandpaper under 1.96 kPa (Figure S13a), the T-PAC maintained high solar reflectance with stable water repellence in the first 50 abrasion cycles and preserved high solar reflectance even after 1000 abrasion cycles (Figure S13b). Although the water contact angle significantly declined from 150.4° to 72.2° after 50 abrasions (Inserted figure of Figure S13b), it can be easily restored by recoating with hydrophobic SiO_2_ NPs. Since T-PAC serves as a surface coating rather than a structural component on costal infrastructures, there is small chance to experience such significant abrasion. Thus, enduring 50 abrasion cycles under ~1.96 kPa should be sufficient to support its coastal applications. Even under extreme abrasion, localized damage can be readily repaired by recoating (Figure S13b), unlike traditional films or membranes that often require complete replacement. Furthermore, in harsher conditions involving highly corrosive solutions with extreme pH values (at pH 0.66 and 13.03), the T-PAC retained stable reflectivity and water repellence with negligible degradation (Figure S14), which were typically detrimental to conventional radiative cooling materials like wood, polymers, and metal-based coatings. In an anti-biofouling test in submerged environment (Figure S15a–i), the T-PAC maintained a clean and white surface after 30 days of immersion, whereas the control sample without superhydrophobic protection (CB-C_2_S) was extensively covered ( ≈ 73.8% area) by yellowish biofouling (Figure S15b, c). This pronounced difference is attributed to the entrapped air layer on the submerged T-PAC, which forms an air/water interface (Figure S5a-ii) that effectively inhibits the contact and subsequent adhesion of microorganisms—preventing anchoring via pili and other cellular mechanisms typically employed at solid/water interface^54^. Consequently, the T-PAC exhibited only a slight decline in *R̅*_solar_. (Figure S15d). This contrast sharply with CB-C_2_S, which suffered severe, irreversible reflectivity loss due to biofouling that blocked light/surface interaction. In addition to these merits, the T-PAC also showed excellent resistance to 1000 h of accelerated UV aging (equivalent to 6 months of natural UV exposure, Figure S16a) and ~90 days of outdoor weathering (Figure S16b). Combining with these attributes, the passive nature of radiative cooling and low cost further endow T-PAC with superior advantages over conventional anticorrosion strategies in durability, energy efficiency, and repairability (Table S3).

To demonstrate the feasibility of transforming advantageous radiative-optical properties into preferential thermal regulation properties, we conducted static indoor and dynamic outdoor cooling measurements based on the coating samples. In static indoor scenario, a high-power Xenon lamp with a uniform power density of 1000 W m^−2^ was used to simulate solar radiation. As depicted in Fig. 4d, the time-dependent temperature variation measured from the coating samples exhibited two discernable stages: an initial rapid rising stage and a subsequent plateau stage with increased irradiation time. The continuous increment of temperature in the first stage is attributed to solar absorption that enables temperature elevation via photothermal conversion. However, at the plateau stage, solar absorption of the coating samples reaches a saturation state that cannot be improved with prolonged irradiation time. Despite similar trends in the evolution of time-dependent temperature variation, these coating samples exhibit remarkable differences in steady-state temperatures and times. Particularly, the uncoated OPC sample shows the highest steady-state temperature of ~50 °C achieved at 15 mins radiation, which contrasts sharply to that of the coated samples. Specifically, for C_2_S coated sample, the steady-state temperature remarkably decreased to ~42 °C at a prolonged radiation time of 35 mins. Coating the OPC sample by carbonated C_2_S further decreased the steady-state temperature to ~40 °C under the same radiation time. A significant drop was observed in the steady-state temperature ( ~ 36 °C) with further BaSO_4_ NPs modification in the carbonated coating layer, while the radiation time remains unchanged. Furthermore, the superhydrophobic SiO_2_ NPs layer exerts negligible influence on the thermal response of the coating. These results highlight the significance of solar reflective properties in regulating the coatings’ thermal response under solar irradiance.

We then conducted continuous outdoor temperature measurements to validate the radiative cooling performance of different coatings under dynamic and complex outdoor conditions in Shenzhen (113° 56′E, 22° 31′N; elevation: 9 m), China. The setup for outdoor experiments is shown in Figure S17a, which is enclosed with aluminum foil on all sides to reduce solar absorption. To more accurately simulate the real-world conditions and provide practically relevant cooling data, the coatings were directly exposed to the air without a wind shelter. The real-time temperatures of the coating samples were monitored using *K* type thermocouples and environmental parameters (i.e., wind speed and relative humidity, Figure S17b) were recorded using a meteorological station. Figure 4e displays the temperature evolution of the experimental samples along with the recorded ambient temperature and solar irradiance. Briefly, all coating samples showed increasing temperatures with rising solar intensity and decreasing temperatures as solar intensity declined. Before sunrise at 6:00 a.m., the temperature variations of the coating samples are dominated by their infrared emissive properties and are irrelevant to their solar reflective properties. Accordingly, in the absence of solar radiation (before 6:00 a.m.), the measured temperatures (*T*_s_) of all coating samples are comparable and about 3.5 °C below the ambient temperature (*T*_amb_) due to high ATW emittance. As the solar intensity increases rapidly after 6:00 a.m., the OPC, C_2_S, C-C_2_S samples constantly absorb solar radiation from the sun, leading to a fast temperature rise in an above-ambient level, underscoring the necessity of effective cooling for coastal structures. Meanwhile, the CB-C_2_S and T-PAC samples exhibited a combined cooling effect enabled by strong infrared emission and solar reflection, resulting in subambient temperature drops even under peak solar intensity at 14:00. Subsequently, as the solar intensity gradually decreases after 14:00, the temperature of the coating samples gradually decreases with continuously narrowing temperature differences. Resulting from excellent thermal radiation performance enabled by high ATW emittance, all the samples cooled to the same temperature when the sun sets after 18:00. During the continuous 24 h test, the CB-C_2_S and T-PAC samples exhibited subambient temperature drop with a temperature fluctuation smaller than 10 °C, which has the potential to suppress thermal stress and Arrhenius-driven steel corrosion in concrete structures. By contrast, the other samples only had subambient temperature drop in the absence of solar radiation (from 00:00 to 6:00 and from ∼18:00 to 24:00) with temperature fluctuation greater than 20 °C. Moreover, the CB-C_2_S and T-PAC samples delivered an average subambient temperature drop of 2.3 °C with a peak value of 4.13 °C and an average temperature drop (compared to the OPC sample) of ~ 25.2 °C with a peak value of ~28.4 °C (Figure S18) in the hottest period (11:00 ~ 13:00) of a sunny day. By contrast, the C_2_S and C-C_2_S samples manifest less pronounced temperature drops.

To theoretically quantify the radiative cooling power of the coating samples under varying conditions, the net cooling power (*P*_net_) and stagnation temperature (*T*_stag_) were calculated based on energy balance analysis^55^, where *P*_net_ represents the cooling power when the sample temperature (*T*_s_) equals the ambient temperature (*T*_amb_), and *T*_stag_ is defined as Δ*T* = *T*_s_ - *T*_amb_ when *P*_net_ = 0. Using equation S4-8, the influence of nonradiative heat transfer coefficient (*h*_c_), *R̅*_solar_, and hydrophobic properties on the *P*_net_ was analyzed, setting the average *T*_amb_ in the day at ~26.65 °C, as determined from outdoor measurements, and different *h*_c_ of 0, 4, 8, and 12 W m^−2^ K^−1^ for varied practical conditions^56^. The calculated results, derived from the spectral reflectivity/emissivity shown in Fig. 4a, are presented in Fig. 4f, demonstrating that, under dry conditions, the *P*_net_ of the optically optimized coating samples (T-PAC) reached ~71.9 W m^−2^ with a *T*_stag_ of ~4.8 °C that is close to the measured value and can be modulate by altering *h*_c_. The *P*_net_ of T-PAC is significantly higher than the -44.3, -92.4, and -484 W m^−2^ calculated from C-C_2_S, C_2_S, and OPC samples, respectively (Figure S19). The discrepancy in *P*_net_ is attributed to thermal gain from solar radiation, which diminishes the cooling efficiency of coatings with lower solar reflectance. Accordingly, optical-optimized coatings with high solar reflectance give rise to improved net cooling power under reduced solar intensity, achieving a maximum value of 112.7 W/m^2^ at a solar irradiance of 0 kW/m^2^ and a minimum value of 66.2 W/m^2^ at a solar irradiance of 1000 kW/m^2^ (Fig. 4g). Notably, even when the hydrophobic properties of the optimized coatings degrade over time, leading to a decline in solar reflectivity due to seawater absorption, the coatings can still recover a high *P*_net_ comparable to their dry-state performance by incorporating evaporative heat transfer (Fig. 4h, S20). These findings indicate the coating’s ability to maintain excellent thermal regulation performance in coastal environment, even with long-term hydrophobic degradation.

### Corrosion inhibition performance of the dual-protective coating

**Fig. 5:**
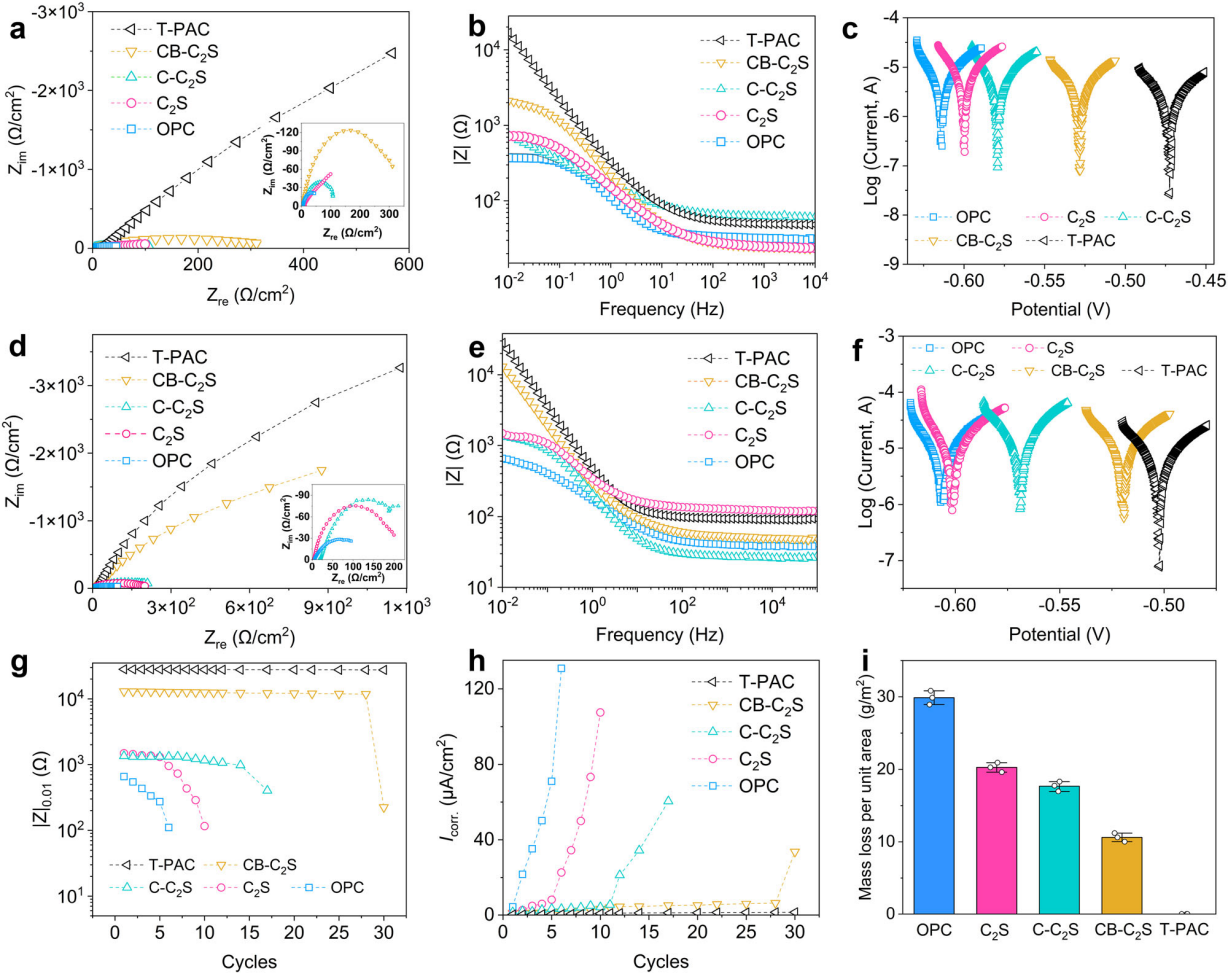
Corrosion inhibition validation. **a-c** Nyquist spectrum, bode plots, corrosion potential (*E*_corr._) and corrosion current (*i*_corr._) of the C_2_S derived coatings after immersed in 3.5 wt.% NaCl for 45 min. Z_im_, Z_re_, and |Z| represent the imaginary part, real part and modulus of the impedance, respectively. **d-f** Nyquist spectra, bode plots, *E*_corr._ and *i*_corr._ of the C_2_S derived coatings after 30 h salt spray treatment. **g** | Z| value of the C_2_S derived coatings at 0.01 Hz ( | Z | _0.01_) in response to the cycles of salt spray and solar radiation treatments. **h** *i*_corr._ of the C_2_S derived coatings in response to the cycles of salt spray and solar radiation treatments. **i** Mass loss per unit area of the C_2_S derived coatings after 30 cycles salt spray and solar radiation accelerated corrosion treatment. The error bars in Fig. 5**i** represent the standard deviation from three replicate experiments. Source data are provided as a Source Data file.

Contrasting sharply with traditional single-function coatings that fail to simultaneously block seawater ingress and reflect solar radiation, the combination of radiative cooling and hydrophobic performance in our study renders the coatings with excellent thermal regulation and corrosive medium blocking capability, which are beneficial for inhibiting steel corrosion in costal concrete structures. In this study, we adopted electrochemical impedance spectroscopy and potentiodynamic polarization techniques to characterize the corrosion resistance of coatings subjected to varied treatment. Prior to the tests, the samples were immersed in 3.5 wt.% NaCl for 45 min to simulate static wetting state. As depicted in Fig. 5a, the Nyquist spectrum of T-PAC sample exhibited the largest semicircle, which is followed by CB-C_2_S, C-C_2_S, C_2_S, and OPC. This implies that T-PAC can effectively protect steel bars from corrosion, and the rest coatings give rise to inferior protection effectiveness. Generally, the low-frequency impedance ( | Z | _0.01_) in the Bode plot reflects the corrosion resistance of coatings. Results in Fig. 5b illustrates that the |Z | _0.01_ value of the T-PAC coated sample was found to be greater than 10^3^ ohms, while the |Z | _0.01_ values for the rest samples were below 400 ohms. The difference is primarily attributed to the excellent hydrophobic performance of T-PAC coating, which effectively prevents the penetration of seawater into concrete structures, thereby inhibiting the ingression of corrosive ions. Further analysis using potentiodynamic polarization technique (Fig. 5c) reveals that the corrosion potential (*E*_corr._) of T-PAC coated sample was -0.47 V, higher than that in the CB-C_2_S, C-C_2_S, C_2_S, and OPC sample (-0.52 V, -0.57 V, -0.6 V,-0.61 V); while the corrosion current (*i*_corr._) of T-PAC coated sample was significantly lower than that of the rest samples. The higher *E*_corr._ and lower *i*_corr._ in the T-PAC coated sample imply that steel corrosion was notably inhibited, ascribed to the corrosive medium blocking effect enabled by exceptional hydrophobic properties.

Comparing the impedance and polarization characteristics after 30 h salt spray treatment allows us better understanding the anti-corrosion performance of coated samples in splash zone of practical scenarios. Figure 5d shows that the T-PAC coated sample after 30 h salt spray treatment still maintained the largest semicircle in the Nyquist spectrum, as compared to that of the rest samples. Likewise, the |Z | _0.01_ value of the T-PAC coated sample at low-frequency (0.01 Hz) in the Bode plot remains to be the highest among the coated samples (Fig. 5e). However, after salt spray treatment, the |Z | _0.01_ value gap between T-PAC and CB-C_2_S coated samples significantly narrowed as compared to that in the untreated samples. This variation could be assigned to reduced hydrodynamic pressure imposed by moisture, which makes it difficult for seawater penetration into the dense microstructure of T-PAC and CB-C_2_S coatings, thus leading to improved corrosion resistance. Despite reduced hydrodynamic pressure, the apparently low initial |Z | _0.01_ values of OPC/C_2_S/C-C_2_S samples remain almost unchanged due to the highly aggressive testing environment. Specifically, the high chloride concentration (3.5 wt.% NaCl) rapidly destabilizes the innate passive film, while the inherent permeability of the three samples facilitates rapid electrolyte saturation, collectively resulting in diminished impedance and premature activation. Consequently, the T-PAC coated sample still delivered the higher *E*_corr_ with relatively lower *i*_corr_ across the experimental samples (Fig. 5f), implying significant steel corrosion inhibition. These findings suggest the significant adaptability and robustness of T-PAC coated sample in corrosion inhibition under dynamic coastal environments involving splash seawater moisture.

To elucidate the advantageous integration of radiative cooling and hydrophobic performance in mitigating rebar corrosion in coastal structures, we continue to implement electrochemical measurements after cyclic salt spray and solar radiation treatments. Prior to the measurements, all coated specimens underwent cyclic treatments, each consisting of 3-hour salt moisture exposure followed by 3-hour 1000 W/m^2^ solar irradiation, with subsequent 18-hour ambient exposure per cycle. We note that the 30 cycles of our test, while equivalent to only 30 days in absolute time, subject the coating to an intensity of coupled thermo-chemo-hygroscopic stress that would be accumulated over a much longer period in a natural marine splash zone environment. Based on an equivalent analysis conducted by a precious study, the 30 cycles of harsh treatment involving cyclic salt-spray and solar illumination should be equal to more than 76 years of natural exposure^57^. Despite this equivalence is not a universal rule, it allows for a quick evaluation of our protective coatings’ durability in a natural marine environment without waiting years for real-world data. The impedance and polarization characteristics, monitored as a function of cycle number and presented in Fig. 5g, revealed that the |Z | _0.01_ value of the T-PAC coated sample remained virtually unchanged throughout cycling, whereas significant declines occurred in comparative samples, indicating differential corrosion resistance degradation induced by solar radiation and salt spray. This demonstrates T-PAC’s superior corrosion resistance attributable to synergistic radiative cooling and hydrophobic properties, which collectively suppress capillary-driven seawater penetration and solar-induced temperature elevation, thereby preserving excellent protective performance. Conversely, other samples exhibited susceptibility to either seawater intrusion or temperature elevation, both accelerating steel corrosion and compromising protective capacity. This combined advantage was further corroborated through continuous monitoring of *i*_corr_ values across treatment cycles (Fig. 5h), where the T-PAC displayed significantly prolonged initiation time for corrosion, as indicated by the delayed turning point relative to counterparts, demonstrating the buffering effect provided by its radiative cooling and hydrophobic properties against corrosion onset in coastal concrete structures.

The enhanced corrosion resistance of T-PAC coated sample was further evidenced by assessing mass loss rate per unit area across the coated samples after 30 cycles (30 corrosion days). As shown in Figure S21, the T-PAC coated sample displayed an intact surface morphology without visually discernable corrosion, contrasting with severe pitting in other specimens. Specifically, the severely degraded, rough surfaces with filamentous rust signs on the OPC, C_2_S, C-C_2_S, and even the CB-C_2_S samples indicate a state of active and non-uniform corrosion. The roughness is a consequence of the widespread breakdown of the passive film and the formation of porous, non-protective corrosion products (e.g., FeOOH, Fe_2_O_3_, Fe_3_O_4_) as chloride ions penetrated the inadequate protective layers. The presence of both corroded and seemingly intact areas on the same rebar, particularly in OPC, C_2_S and C-C_2_S, suggests the formation of localized macro-cells, where anodic sites (corroding) and cathodic sites (passive) coexist, accelerating the deterioration process. In stark contrast, the smooth and pristine surface of the T-PAC sample is direct visual proof of its exceptional barrier property. The coating successfully prevented chloride ions and moisture from reaching the steel surface, thereby preserving the initial passivity and preventing any visual corrosion initiation. The superior surface condition of the T-PAC sample validates the synergistic protective effect of its integrated radiative cooling and superhydrophobic layers. Correspondingly, the mass loss rate per unit area (Fig. 5i) of the T-PAC coated sample decreased to nearly 0 g/m^2^, substantially lower than OPC (29.9 g/m^2^), C_2_S (20.3 g/m^2^), C-C_2_S (17.7 g/m^2^), and the CB-C_2_S sample (10.6 g m^2^) under the identical conditions, confirming effective corrosion suppression through integrated radiative-cooling and hydrophobic mechanisms.

### Mechanism analysis of anti-corrosion performance based on gel mechanism, phase assemblage, interface adhesion and synergistic behavior

The significantly enhanced anti-corrosion performance observed in the C_2_S-deviated samples are highly correlated to their distinct hydration or gelation mechanisms, phase assemblage, microstructure, and interfacial adhesion characteristics. In a pure C_2_S system, the basic hydration process follows the pathway [2(2CaO·SiO_2_) + 4H_2_O → 3CaO·2SiO_2_·3H_2_O + Ca(OH)_2_], which proceeds at a low rate due to the limited solubility of C_2_S in neutral water. Although a superficial hydration layer comprising calcium hydroxide (portlandite) and C-S-H gel may form on C_2_S particles, the XRD pattern (Fig. 2j) reveals no discernible diffraction peaks corresponding to portlandite. Instead, the dominant crystalline phases are identified as orthorhombic *γ*-C_2_S and metastable calcium carbonate polymorphs (aragonite and vaterite). The minimal content of calcium carbonate, as confirmed by TGA, further corroborates the low hydraulic reactivity of C_2_S. Consequently, the pristine C_2_S coating consists of discrete and irregular columnar C_2_S particles, forming a hierarchically porous structure (Fig. 2i) characterized by numerous randomly distributed micro-voids (Figure S5a). While such a porous architecture may favor sunlight scattering, the intrinsic absorption of C_2_S within the solar spectrum limits the overall reflectance to 76.14%, thereby yielding only a modest reduction [(*i*_corr._OPC_ – *i*_corr_C2S_) / *i*_corr._OPC_ = 17.8%] in the temperature-dependent corrosion rate. Furthermore, in conjunction with the abundance of polar groups inherent to hydrophilic C_2_S, the porous structure facilitates capillary water absorption (Fig. 3f) by providing continuous pathways for chloride-laden seawater ingress. This leads to the earliest onset of corrosion and the highest corrosion rate among all coated samples (Fig. 5h). Moreover, the discrete C_2_S particles are primarily deposited on the substrate through physical adhesion, which lacks effective interparticle bonding to withstand long-term dynamic impacts in harsh marine environments. Thus, the pristine C_2_S coating fails to provide an effective physical barrier against seawater penetration and lacks the durability required for prolonged service in coastal areas, resulting in inferior anti-corrosion performance.

Although C_2_S exhibits limited hydraulic reactivity, it can be activated via carbonation in the presence of CO_2_ and moisture, serving as a carbonatable binder that facilitates powder consolidation. The fundamental gelation mechanism of carbonated *γ*-C_2_S involves a phase transition in a CO_2_-rich environment, as expressed by the reaction: C_2_S + 2CO_2_ + 2H_2_O → CaCO_3_ (Calcite/Aragonite) + SiO_2_·nH_2_O (Calcium-modified silicate gel)^58^. Under a CO_2_ concentration of 20% and relative humidity of 70%, the carbonation products comprise calcium-modified silica gel and CaCO_3_ polymorphs (aragonite and vaterite), as verified by XRD and ATR-FTIR analyses (Fig. 2j, k). Although the phase assemblage appears similar to that of pristine C_2_S, quantitative TGA (Fig. 2l) reveals a substantially higher CaCO_3_ content in the carbonated sample. This contributes to a moderate increase in solar reflectance from 76.14% to 84.44% (Fig. 4a), which in turn reduces the Arrhenius-driven corrosion kinetics, yielding a 53.8% decrease in corrosion rate (Supplementary note 8). Structurally, the carbonation transforms the initially loose *γ*-C_2_S powder into a compact matrix of CaCO_3_ with markedly reduced porosity (Fig. 2i), thereby enhancing the physical barrier properties and suppressing capillary water uptake (Fig. 3f) due to reduced pathway for seawater transportation. The combined effect of improved solar reflectance and enhanced barrier performance leads to enhanced anti-corrosion performance relative to the pristine C_2_S coating (53.8% *vs*. 17.8% of *i*_corr._ reduction compared to OPC, Fig. 5h). Further examination of microstructure (Figure S5b) found that the carbonated matrix is formed by continuous calcium carbonates encapsulating the unreacted particles and silica gels. This configuration enhances interparticle bonding, thereby improving the mechanical robustness of the coating. In addition, the increased compactness and the formation of chemical bonds at the C-C_2_S/substrate interface^46^ enhance mechanical interlocking, leading to a significantly improved interfacial debonding strength of approximately 1.86 MPa (Figure S22). These enhancements in both coating cohesion and interfacial adhesion are critical for withstanding harsh marine conditions and ensuring long-term corrosion protection.

The gelation mechanisms of CB-C_2_S and T-PAC are identical, as the hydrophobic SiO_2_ overlayer is deposited after the gelation of BaSO_4_-modified carbonated C_2_S layer. However, they differ substantially from those of C-C_2_S and pristine C_2_S due to the incorporation of BaSO_4_ NPs. Generally, the carbonation gelation path of pure *γ*-C_2_S follows a kinetic control process, in which the precipitation of CaCO_3_ occurs when the concentration of Ca^2+^ and CO_3_^2+^ ions reached an high supersaturation. In this process, CaCO_3_ nucleation proceeds predominantly via homogeneous nucleation—either within the solution or on non-ideal substrates such as silica gel formed during the reaction. This homogeneous nucleation process is associated with a high energy barrier (ΔG*__hom_ ≈ 7.03 × 10^−19^ J, Supplementary Note 4), placing the system under kinetic control and favoring the formation of faster-to-nucleate metastable aragonite and vaterite, rather than the stable calcite phase that requires a higher critical nuclear size. In contrast, the introduction of chemically inert BaSO_4_ NPs offers favorable heterogeneous nucleation sites for CaCO_3_ precipitation. Crucially, the (001) plane of BaSO_4_ (barite) exhibits a high degree of lattice matching with the (104) plane of calcite in terms of atomic arrangement and the interionic spacing (Ca^2+^ spacing of CaCO_3_ ~ 4.99 Å) of CaCO_3_ is relatively close to BaSO_4_ (Ba^2+^ spacing of BaSO_4_ ~ 5.2 Å), with a lower degree of mismatch. This structural compatibility allows Ca^2+^ and CO_3_^2+^ ions to easily arrange on the surface of BaSO_4_ particles, growing according to the crystal structure of calcite, a phenomenon called ‘epitaxial growth’. Such epitaxial nucleation markedly reduces the energy barrier (ΔG*__het_ ≈ 8.96 × 10^−21^ J, Supplementary Note 4) required for calcite nucleation on the BaSO_4_ NPs surfaces. As a result, the BaSO_4_ NPs function as a preconfigured template that promotes the preferential crystallization of calcite via a heterogeneous nucleation pathway, thereby suppressing the formation of metastable aragonite and shifting the crystallization regime from kinetic to thermodynamic control. This BaSO_4_-induced transition accounts for the observed aragonite-to-calcite polymorphic transformation (Fig. 2j–l) in both CB-C_2_S and T-PAC samples.

Theoretically, the calcite^59^ possesses a bandgap ~1.0 eV wider than that of aragonite (calculated to be 4.23 eV)^60^, which contributes to its higher solar reflectance^53^. Combined with the increased scattering interfaces introduced by BaSO_4_ NPs, the aragonite-to-calcite polymorphic transition in CB-C_2_S and T-PAC results in a remarkable improvement in solar reflectance, from 76.14% to ~94.35% (Fig. 4a). This high solar reflectance, which surpasses the 90% threshold for effective daytime radiative cooling, significantly reduces heat gain by rejecting solar radiation and resulted in a notably lower surface temperature, with a maximum drop of 28 °C compared to ordinary Portland cement (OPC), thereby attenuating chloride ion transport by 66.8% (Supplementary Note 6) and consequently suppressing the Arrhenius-driven corrosion kinetics by 74.3% (Fig. 5h). Detailed microstructure analysis further reveals that BaSO_4_ NPs modification and the associated polymorphic transition yield a denser coating morphology with reduced micro-scale porosity and optimized nano-scale pore structure (Fig. 2i) due to pore-filling effects. This trend is more pronounced in T-PAC, suggesting that hydrophobic SiO_2_ NPs penetrate the rough surface of underlying BaSO_4_-modified carbonated C_2_S layer, further reducing micro-pores and enhancing mechanical interlocking between the SiO_2_ overlayer (Figure S23) and the radiative cooling underlayer via van der Waals forces and hydrogen bonding. Although a denser microstructure generally improves mechanical robustness and interfacial adhesion, the partial replacement of cementitious binder by BaSO_4_ NPs in CB-C_2_S and T-PAC results in a slightly lower adhesion strength (1.61 MPa) compared to that of C-C_2_S (1.86 MPa). Nonetheless, this value still exceeds typical cementitious coating bonding strengths (0.4–0.8 MPa) reported in the literature^51^, thus is strong enough to ensure anti-corrosion durability under coastal environment. Moreover, the denser morphology with reduced overall porosity in CB-C_2_S effectively limits capillary water absorption (Fig. 3f), delaying corrosion initiation by ~2.5 times compared to C-C_2_S (Fig. 5h). With the additional barrier provided by the superhydrophobic SiO_2_ overlayer, T-PAC exhibits the best performance in inhibiting seawater penetration and chloride ingress, leading to superior corrosion resistance through the combined effects of ion-blocking and cooling-enabled corrosion buffering.

Quantitively assessing the individual contribution of radiative cooling to corrosion suppression provides deeper insight into how it works synergistically with superhydrophobic properties to impart enhanced anti-corrosion performance. For two different temperatures of ${T}_{1}$ (uncoated) and ${T}_{2}$ (coated), the relationship between temperature and *i*_corr._ follows the Arrhenius equation (Supplementary Note 5). Based on temperature measurements (Fig. 4e) during the peak solar period (11:00-13:00), where the average temperatures were 24.53 °C (coated) and 49.55 °C (uncoated), the calculated corrosion current density ratio is *i*_corr._coated /_ *i*_corr._uncoated_ = 0.207 (Supplementary Note 5). This indicates that passive cooling alone reduces the corrosion rate by 71.6%, which aligns closely with the experimentally measured reduction of 74.3% (calculated as [*i*_corr._OPC_ – *i*_corr_CB-C2S_] / *i*_corr._OPC_). The minor discrepancy ( ~ 2.7%) is attributable to an additional physical barrier provided by the CB-C_2_S layer, which leads to an underestimation in the theoretical calculations of the corrosion rate–temperature relationship. To quantitatively address the multifaceted impact of radiative cooling on chloride transport and corrosion processes, we then performed a comprehensive analysis based on established physical models and our experimental data. The temperature dependence of the chloride diffusion coefficient follows the Arrhenius-type relationship derived from the transition state theory for ionic transport in porous materials^61^. Using the same temperature data applied in *i*_corr._ calculation, a 66.8% reduction in the chloride diffusion coefficient is obtained (Supplementary Note 6), which is consistent with the experimental results (69.05%) of a previous study^61^, conducted at similar temperature conditions. Furthermore, the extension in initiation time enabled by radiative cooling is also quantified to give a detailed analysis of its impact on the corrosion behavior. The time for chloride ions to reach the critical concentration at the steel surface can be estimated by the error function solution to Fick’s second law under non-steady-state conditions for a semi-infinite medium^62^. Considering both the reduced diffusion coefficient and the increased critical chloride threshold, radiative cooling is estimated to extend the corrosion initiation time by a factor of 15.1 in CB-C_2_S coated specimen compared to uncoated OPC (Supplementary Note 7), highlighting its significant role in retarding corrosion initiation.

To further elucidate the synergistic behavior between radiative cooling and superhydrophobicity within our coating system, a stepwise analytical framework (Supplementary Note 8) was employed to deconvolute their individual and combined contributions on the corrosion behavior, with each step building upon the previous one to quantify the added value of each component, using our experiments data: *i*_corr._OPC_ (130.868 μA/cm^2^), *i*_corr._C-C2S_ (60.447 μA/cm^2^), *i*_corr._CB-C2S_ (33.644 μA/cm^2^), and *i*_corr._T-PAC_ (1.453 μA/cm^2^). In the analytical model, carbonation (C-C_2_S) alone provides a baseline improvement, reducing *i*_corr_ by ~54% compared to OPC, establishing the baseline upon which the individual functionalities are added. Then, the *i*_corr._ reduction of radiative cooling is calculated to be 26.803 / 60.447 ≈ 44.3% relative to the dense barrier baseline and the net contribution of superhydrophobicity is calculated to be 32.191 / 33.644 ≈ 95.7% relative to the baseline (Barrier + Cooling). If the effects were purely multiplicative, the theoretical *i*_corr._ of T-PAC would be 2.94 μA/cm^2^. However, the actual measured *i*_corr._ of the full T-PAC system is 1.453 μA/cm^2^, which is 50.6% lower than this theoretical multiplicative value, demonstrating that the combination achieves substantially better performance than predicted by simple additive models. The synergy between radiative cooling and superhydrophobic barrier can be further quantified using a Synergy Index (SI, a probability model for independent events) calculated as: SI = (PE__combined_) / (PE__cooling_ + PE__barrier_ - PE__cooling_ × PE__barrier_), in which PE__barrier_ represents the estimated protection efficiency of the superhydrophobic barrier alone. Based on the performance improvement observed when individually adding the superhydrophobic barrier or the radiative cooling functionality to the system, we estimate the synergy index to be 1.013. An SI > 1 quantitatively demonstrates that the two functions are not merely independent but interact synergistically, producing a combined effect that surpasses the theoretical prediction.

**Fig. 6:**
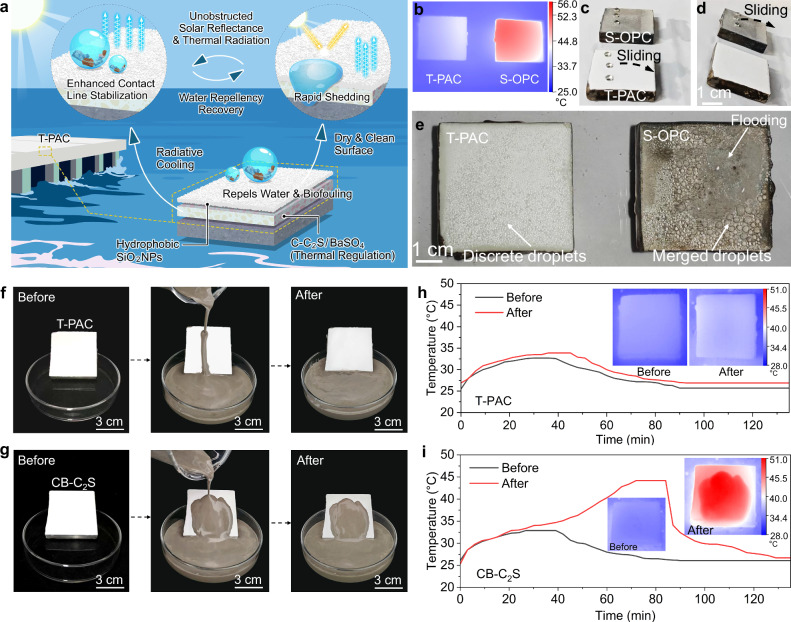
Mutual protection mechanism of radiative cooling and superhydrophobic property in T-PAC. **a** Schematic diagram showing the interactive cooperation of radiative cooling and superhydrophobic property for dual-protection. Briefly, the superhydrophobic SiO_2_ layer keeps the radiative cooling layer dry and clean for unobstructed solar reflection and ATW emission, while the cooler’s temperature regulation reduces surface condensation and facilitates the recovery of water repellency, which overcomes the inherent limitations of each technology when used independently for corrosion protection. **b** Infrared image showing the temperature difference of T-PAC and superhydrophobic ordinary Portland cement (S-OPC) sample under simulated solar illumination (1000 W/m^2^). **c** Droplets on low-temperature T-PAC begin to slide under a tilting angle of ~3°. **d** Droplets on S-OPC sample with elevated temperature begin to slide under a tilting angle of ~26.5°. **e** Condensation behavior of T-PAC and S-OPC samples under simulated conditions combining sunlight illumination and high humidity. **f** Contamination test showing the self-cleaning capability of T-PAC. **g** Contamination test showing the fouling susceptibility of carbonated BaSO_4_-modified C_2_S (CB-C_2_S). **h** Temperature variation of T-PAC before and after contamination treatment. Inserted figures are infrared images showing the temperature of T-PAC before and after contamination treatment. **i** Temperature variation of CB-C_2_S before and after contamination treatment. Inserted figures are infrared images showing the temperature of control before and after contamination treatment. Source data are provided as a Source Data file.

The synergy operates in a mutually protective and enhancing manner through the following mechanisms (Fig. 6a): On the one hand, the radiative cooling layer does more than just slow down electrochemical kinetics, it enhances anti-wetting performance by promoting rapid surface drying through sub-ambient cooling, since a lower temperature thermally stabilizes the three-phase contact line of seawater during dry intervals, which reduces surface condensation and facilitates the recovery of water repellency. As shown in Fig. 6b, under continuous irradiation at ambient humidity (Figure S24a), the surface temperature of T-PAC was ~18.6 °C lower than that of the control (a superhydrophobic OPC sample, S-OPC), and its water droplets slide off at a tilt angle of only ~3° (Fig. 6c), compared to ~26.5° for the control (Fig. 6d), indicating maintained Cassie–Baxter stability. Moreover, under sustained irradiation while humidity was raised to >90 % RH (Figure S24b), T-PAC exhibited discrete, fine condensation droplets (<1 mm), characteristic of dropwise condensation, whereas the control sample showed coalesced droplets and film-wise condensation (Fig. 6e), signaling a transition toward the Wenzel state. These results indicate that the radiative cooling layer protects the superhydrophobic layer via stabilizing solid–liquid–air interface, thereby suppressing the wetting transition and reduces condensation under coupled thermal-humid conditions. The enhanced stability is also reflected by outdoor cooling measurements ( ~ 2.95 °C sub-ambient temperature drop) under high-humidity weather (Figure S25), despite traditional radiative coolers perform optimally in hot, dry climates. On the other hand, the superhydrophobic layer preserves the radiative cooling performance via preventing seawater absorption or fouling adhesion, thus ensuring sustained high solar reflectance and unobstructed mid-infrared thermal emissions to the sky. Our tests corroborate this mechanism, showing that even after being treated to viscous mud slurry, the T-PAC recovered its clean and white surface (Fig. 6f) with its thermal regulation performance remains unchanged upon fouling shedding (Fig. 6h), demonstrating that the superhydrophobic layer effectively preserves optical efficiency of the radiative cooling layer. This capability is of great significance for durable corrosion protection, as a fouled or wet surface (Fig. 6g) would suffer from plummeting reflectance and a consequent dramatic increase in solar absorption, thereby severely compromising cooling performance (Fig. 6i). These results indicate that the radiative cooling and superhydrophobic functionalities in our design are not simply additive but operate in a mutually reinforcing and protective manner, which overcomes the inherent limitations of each technology when used independently, rendering the T-PAC uniquely suited for real marine applications.

Based on the above analysis, the T-PAC enables multi-stage corrosion suppression via comprehensive defense through complementary mechanisms (Figure S26). During the corrosion initiation stage, the superhydrophobic layer serves as the primary defense, physically blocking >90% of chloride-laded seawater ingress (Fig. 3g). The cooling function acts as a secondary defense, reducing the chloride diffusion coefficient by ~66.8% and significantly delaying the time for residual chlorides to reach the steel surface. In the corrosion propagation stage, once chlorides breach the barrier, the cooling layer becomes the primary defense, suppressing the corrosion reaction rate by 74.3% via Arrhenius kinetics (Fig. 5h). Therefore, the superhydrophobic layer and radiative cooling function operate in a cooperative manner: the former drastically reduces initial chloride ingress, while the latter retards the diffusion and electrochemical kinetics of any residual chlorides. This synergy effectively mitigates the individual failure modes of each component, resulting in a composite coating whose overall anti-corrosion performance exceeds the sum of its parts, achieving near-complete corrosion suppression. Compared to conventional protection strategy, our T-PAC coating establishes a paradigm shift by moving beyond single-mechanism protection, which simultaneously suppresses the Arrhenius-type kinetic acceleration of corrosion and capillary seawater absorption, positioning the T-PAC system as a comprehensive solution to the coupled thermo-chemo degradation inherent to marine environments.

## Discussion

In this study, we establish a temperature-wetting mediation paradigm for corrosion protection, demonstrating that the synergistic integration of radiative cooling and superhydrophobicity in C_2_S-based coatings fundamentally disrupts corrosion pathways in marine concrete. Through spectral engineering ( ~ 94.6% solar reflectance plus 92.8% ATW emittance) and superhydrophobic structure (151.3° contact angle), the T-PAC coating simultaneously achieves sub-ambient cooling (Δ*T* = -4.13 °C) and near-zero water permeability, addressing the dual corrosion accelerators—chloride ingress and solar-driven thermal acceleration. Under accelerated aging representing 30 days of salt-spray and solar irradiation cycling, the coating exhibited unparalleled stability: |Z | _0.01_ remained virtually unchanged, *I*_corr_. increased by merely 0.02 μA/cm^2^, and mass loss was suppressed to below 1 g/m^2^—an order of magnitude lower than control samples. Crucially, we identified that radiative cooling and superhydrophobicity operate via complementary mechanisms: radiative cooling suppresses Arrhenius-type kinetic acceleration by reducing thermal load, while superhydrophobic property disrupts electrolyte percolation through Cassie-state stabilization. This work thus provides a paradigm shift for multi-physics corrosion inhibition, in which temperature-wetting regulation replaces conventional single-mechanism barrier strategies, with material principles extendable to a range of structures exposed to hygrothermal extremes.

## Methods

### Raw materials

Dicalcium silicate (C_2_S, >99% purity, *γ*-phase, average particle size: 15 μm, Figures S27) was purchased from Home of Single Mine Co., Ltd. (China). Barium sulfate nanoparticles (BaSO_4_ NPs, 99.9% purity, average particle size: 480 nm, Figures S3) were obtained from Xi-Long Scientific Co., Ltd. (China). Compared to high-performance alternatives such as *h*-BN and Al_2_O_3_, BaSO_4_ is more chemically stable and cost-effective. 42.5-grade ordinary Portland cement was provided by Shouxin Cement Group Co., Ltd. Deionized water was produced using a Milli-Q water purification system (Millipore, USA). Commercial solution of superhydrophobic SiO_2_ NPs was purchased from Schanda Chemical Co. Ltd. (Foshan, China). The water-reducing agent used in this study was a modified polycarboxylate ether (Basf, MELLUX ® 2651 F) synthesized via a spray-drying process. Analytic grade sodium chloride (NaCl, 99% purity) was obtained from sigma-Aldrich. Epoxy-resin was supplied by Leaftop Co., Ltd. (China). All reagents were used without further purification. Q235 steel bars, aluminum foil, acrylic boxes (30 × 30 × 30 cm), and spray coating equipment were purchased from a local supplier.

### Preparation of cement paste substrate embedded with a steel rebar

Cement pastes substrates (50 × 50 × 10 mm^3^) were prepared for coating deposition and electrochemical measurements. A steel reinforcing bar (Ø 0.3 cm × 7 cm length), which had been polished to a smooth surface to eliminate surface defects, was partially embedded into each specimen. Two ends of the rebar were sealed with epoxy resin to confine the electrochemically active surface area. The paste was formulated at a water-to-cement mass ratio of 0.4, mixed at low speed for 2 min followed by high-speed stirring for another 2 min (with a 20-second interval). The fresh paste was cast into steel molds and vibrated for 45 s to remove entrapped air. After casting, the specimens with embedded steel bars were covered with plastic film and cured under ambient conditions for 24 h, followed by standard curing at ~25 °C and ~95% relative humidity for 27 days prior to coating application.

### Synthesis of C_2_S/BaSO_4_ Composite Gel Coating

The T-PAC coating was fabricated via a sequential process. First, a composite slurry was prepared by mixing *γ*-C_2_S powder with a water-reducing agent (mass ratio: 1:0.06) for 2 min. Separately, BaSO_4_ nanoparticles (10–50 wt.% relative to C_2_S) were dispersed in deionized water (50 wt.% relative to C_2_S) using ultrasonic treatment (40 kHz, 600 W, 15 min). The BaSO_4_ dispersion was then incorporated into the C_2_S mixture under mechanical stirring (500 rpm, 4 min) to form a homogeneous slurry. This slurry was brush-applied onto pre-prepared cement paste substrates to achieve a wet thickness of approximately 50–300 μm, followed by a 24-hour ambient drying period. The dried coating was subsequently carbonated in a controlled chamber (20% CO_2_, 70% RH, 25 °C) for durations ranging from 15 to 240 min, transforming it into a dense, rigid composite of silica gel, BaSO_4_, and CaCO_3_. Finally, a 10 wt.% suspension of hydrophobic SiO_2_ nanoparticles in ethanol was spray-coated onto the carbonated surface using an airbrush (nozzle: 0.3 mm, pressure: 0.2 MPa), resulting in a top layer of 16–100 μm. The bilayer coated sample was then cured for 30 min at ambient conditions to allow solvent evaporation, forming the hierarchical superhydrophobic structure. The mass of deposited SiO_2_ was controlled by the spraying time and confirmed gravimetrically.

To demonstrate the advantages of T-PAC in corrosive medium blocking and radiative cooling-induced corrosion suppression, three control coatings were prepared under identical processing protocols: (i) Control 1 (C_2_S), a pure *γ*-C_2_S paste without carbonation or additives; (ii) Control 2 (C-C_2_S), a carbonated *γ*-C_2_S coating, lacking both BaSO_4_ nanoparticles and the superhydrophobic SiO_2_ overlayer; and (iii) Control 3 (CB-C_2_S), a radiative cooling coating composed of carbonated *γ*-C_2_S and BaSO_4_ nanoparticles, but without the superhydrophobic SiO_2_ top layer. For Controls 2 and 3, the formulations included deionized water and BaSO_4_ nanoparticles each at 50 wt.% relative to C_2_S, and both underwent a 30-minute carbonation treatment (20% CO_2_, 70% RH, 25 °C) to ensure a consistent baseline.

### Multiple analytic characterization methods

#### Micromorphology, phase identification, functional groups, TGA and size distribution

Scanning electron microscopy (SEM, Su-70, Hitachi, Japan) was used to analyze surface morphology (accelerating voltage: 20 kV, working distance: 5 mm). To assess the average roughness of the experimental samples, a 3D nano-profiler equipped with a green light tip was employed. The size of the scanning area was set at 300 µm × 500 µm. Furthermore, a mercury intrusion porosimeter (Autopore, V 9600, USA) was used to investigate the pore size distribution at a maximum pressure of 420 MPa. The contact angle and surface tension were 130° and 485 mN/cm, respectively. X-ray diffraction (XRD, Bruker D8 Advance) with Cu-Kα radiation (λ = 1.5406 Å, 40 kV, 40 mA) was employed to identify crystalline phases (scan range: 5°–70°, step size: 0.02°). Fourier-transform infrared spectroscopy (FTIR, Thermo Nicolet Nexus 670, USA) was conducted in attenuated total reflectance mode (400–4000 cm⁻¹, resolution: 4 cm⁻¹) for amorphous phase identification. The experiments were conducted in the wavenumber range of 400 ~ 4000 cm^−1^ with 64 scans for each test. Constituent variation was determined using a NETZSCH STA 409 PC TGA system. For each test, about 20 mg of powder was heated from ambient temperature (25 °C) to 900 °C with a heating rate of 10 K/min. Laser diffraction particle size analysis was performed on solid powder samples using a laser granulometer Helos from Sympatec GmbH. The measurements were conducted at a wavelength of 632.8 nm with a detection range from 0.1 µm to 200 µm.

#### Spectral characterization and thermal-regulation performance evaluation

Hemispherical reflectivity within the solar band (0.28–2.5 μm) and infrared emissivity (3–15 μm) were measured using a UV-visible−near IR spectrometer (SolidSpec-3700) equipped with a polytetrafluoroethylene integrating sphere and an FT-IR spectrometer equipped with a diffuse gold integrating sphere (Bruker Vertex 70), respectively. The emittance in the mid-infrared region within the transparent atmospheric window (*ɛ̄*_ATW_) was calculated by averaging the emittance data over the wavelengths of 8–13 μm (detailed calculations are provided in the Supporting Information). For each sample, the averaged data and corresponding standard deviation were obtained based on a minimum of 3 measurements.

The scattering behavior of the BaSO_4_ with varied particle sizes was investigated by Finite-difference time-domain simulations using Lumerical FDTD solutions. The simulation focused on the near-field electromagnetic distribution of solar photons in the solar spectrum. To streamline the simulation process and simplify calculations, a two-dimensional model was employed. Perfect matching layer (PML) absorption boundary conditions and the full-field scattering field (TFSF) source were utilized to ensure accurate results. The diameter of BaSO_4_ nanoparticles varied between 50 and 1000 nm, with an overlapping range of 0 to 1, to explore the effects of nanoparticle size on the scattering behavior. These parameter variations were chosen to achieve comprehensive coverage and capture a wide range of potential scenarios.

For thermal performance evaluation, a Xenon lamp (Zhongjiao Jinyuan, HXF300) emulating the solar spectrum generated spatially uniform irradiation at ~1000 W/m^2^ on sample surfaces in static indoor conditions. Samples were positioned atop insulating polystyrene foam to minimize conductive/radiative thermal interference from the substrate, with temperatures recorded via a multi-channel thermometer (Toprie, TP700) using three *K*-type thermocouples. Complementarily, outdoor evaluations employed a custom apparatus comprising two circular grooves (Ø8.6 cm × 0.5 cm depth) centered within 30 cm^3^ foam boxes. Experimental samples covered each groove, while aluminum foil encapsulation mitigated ambient radiative effects. The assembly was elevated 0.6 m above ground, with groove temperatures monitored by embedded thermocouples and ambient temperature tracked externally. Concurrent meteorological parameters (wind speed, relative humidity, ambient temperature, solar irradiance) were logged by an RS485 station (JianDaRenKe). Continuous thermal measurements were conducted under direct sunlight on a building rooftop in Shenzhen, China (22°31’N, 113°56’E; 9 m elevation).

#### Anti-wetting and anti-corrosion assessment

Water contact and roll-off angles were measured using a Dataphysics OCA20 system (Dataphysics, Germany). For static contact angles, a 5 μL water droplet was deposited on the sample surface via motorized syringe. Upon droplet stabilization, a side-view image was captured, with the static contact angle determined by system software analyzing the droplet profile. Roll-off angle measurements employed a manually operated tilt stage: beginning at 0° inclination, a 5 μL droplet was deposited, followed by gradual stage tilting until droplet movement initiated. The tilt angle at this instant, defined as the roll-off angle, was recorded. To ensure statistical reliability, triplicate measurements were performed per sample at distinct surface positions.

Electrochemical impedance spectroscopy and potentiodynamic polarization tests of the coated samples were conducted in a neutral salt mist (ASTM B117) environment. In order to realize one-dimension chloride ingress, only one surface of each specimen was exposed to simulated environments while other surfaces were firmly sealed by epoxy-resin. A salt mist corrosion tester (Dongguan LESTEST Equipment Co., Ltd.) was used and the amount of salt-spray deposition was controlled to be 1.5 mL/(cm^2^·h), which met the requirement of Chinese standard GB/T 5170.8. The sample was exposed to salt mist containing 5 wt% NaCl for continuous 180 min under 35 °C (Figure S9), followed by solar illumination at ~1000 W/m^2^ for another 180 min. The electrochemical measurements of the coating were conducted by using CHI 660D electrochemical workstation (Princeton applied research Model 283 Potentialstat/Galvanostat) under steady open-circuit voltage. Figure S28 schematically illustrates the configuration of the three-electrode electrochemical cell for the electrochemical tests. The working electrode was a two-end sealed Q235 steel bar (Ø0.3 cm × 7 cm) partially embedded in a coated sample (50 mm × 50 mm × 10 mm). The counter electrode and reference electrode were the platinum electrode (Type 213, Shanghai Precision Scientific Instrument Co. Ltd.) and the saturated calomel electrode (Type 232, Shanghai Precision Scientific Instrument Co. Ltd.). The scanning rate of the polarization curve was 0.1667 mV/s, the electrochemical impedance spectroscopy was conducted within the frequency scope of 10^−2^ Hz ~ 10^5^ Hz with a 5-mV amplitude Sine wave. Each test was repeated more than three times to guarantee the accuracy of the experiment result.

### Robustness and durability assessments

#### Interfacial adhesion assessment

The adhesion between the superhydrophobic SiO_2_ overlayer and the carbonated gel substrate was evaluated following the ASTM 5B standard. A cross-cut pattern with a 1 mm grid spacing was first introduced on the coating surface using a QHF cutting knife. 3 M™ 600 tape was then firmly applied over the grid area, and a 2 kg load was used to ensure intimate contact. After a dwell time of 90 s, the tape was rapidly peeled off at a 90° angle. This peel-off procedure was repeated twice on the same test area. The substrate was subsequently inspected to assess the extent of coating removal, thereby quantifying the interfacial adhesive strength.

#### Accelerated UV irradiation weathering test

The UV durability of the T-PAC coating was assessed using a QUV accelerated weathering tester (Q-Lab). The apparatus was equipped with UVA-340 fluorescent bulbs to simulate the medium-to-short wavelength ultraviolet spectrum of sunlight. To accelerate material degradation, the samples were subjected to a continuous UV irradiance of 0.89 W m^−2^ at 340 nm and a constant temperature of 60 °C for a total duration of 1000 h (approximately 42 days). The cumulative UV dose delivered in this test was ~275 MJ m^−2^, aligning with an established international benchmark for evaluating the durability of construction materials.

#### Abrasion tolerance evaluation

The abrasion resistance of the T-PAC coating was evaluated using a linear abrasion test. A coated sample was placed face-down on a 400-grit sandpaper. A normal load of 500 g was applied to ensure uniform contact between the coating surface and the abrasive medium. The sample was then pulled horizontally at a constant speed of ~0.02 m/s over a travel distance of 20 cm per cycle. This procedure was repeated, and the solar reflectance of the coating was measured at pre-designed abrasion cycles to quantify the degradation of its optical performance.

#### Corrosive solution resistance test

The chemical stability of the T-PAC coating was assessed using an immersion method. Coated samples were fully immersed in aggressive aqueous solutions with pH values of 0.66 and 13.03, which were prepared by titrating with 18.4 mol/L hydrochloric acid and 1 mol/L sodium hydroxide, respectively. After an immersion duration of 15 min, the samples were removed, rinsed with deionized water, and subsequently dried in an oven at 40 °C for 120 min to ensure complete moisture evaporation. The chemical resistance was then quantified by comparing the solar reflectance and water contact angle of the samples before and after immersion.

#### Anti-biofouling test

A biofouling test was performed in a simulated marine environment using a transparent glass aquarium (45 × 45 × 45 cm^3^). The tank was filled with ~91 L of synthetic seawater (salinity: 33 g/L) equipped with an Eheim 250 filter to maintain water circulation. To mimic a natural ecosystem, artificially cultivated coral stones were introduced as substrates. The system was inoculated with 10 mL of commercial nitrifying bacteria to establish a functional nitrogen cycle and illuminated by a 100 W LED lamp (400–750 nm) for 8 h daily to promote microbial and biofilm growth. Following a 7-day stabilization period, T-PAC and CB-C_2_S samples (50 × 50 × 10 mm^3^) were immersed and exposed to this accelerated biofouling condition for 30 days. The anti-biofouling efficacy was determined by quantifying the surface biofouling coverage and measuring the change in solar reflectance of the samples before and after the exposure period.

## Supplementary information

Supplementary Information

Transparent Peer Review file

## Source data

Source Data

## Data Availability

All data are available in the main text or the supplementary information. Source data are provided with this paper.
