# Supplementary material for: Dual-path suppression of thermal and wetting-driven steel corrosion for marine structure
Source: Nat Commun. 2026 Apr 21;17:5471. doi: 10.1038/s41467-026-71930-x (PMC13284360; doi:10.1038/s41467-026-71930-x)
Supplement: Supplementary file 1 — Supplementary Information [file 41467_2026_71930_MOESM1_ESM.pdf]

Supporting information

Dual-Path Suppression of Thermal and Wetting-Driven Steel Corrosion for Marine Structure

Xiantong Yan[1], Fan Zhang[1], Shirui Peng[1], Hanya Yan[1], Meng Yang[2], Wenhui Duan[3], Xiaohua Bao[1], Xiangsheng Chen[1], Hongzhi Cui[1, *]

[1]State Key Laboratory of Subtropical Building and Urban Science, GuangDong Engineering Technology Research Center of Low-Carbon and Energy Efficiency in Buildings, College of Civil and Transportation Engineering, Shenzhen University, Shenzhen 518060, China

[2]Department of Materials Science and Engineering, Southern University of Science and Technology, Shenzhen, Guangdong 518055, China

[3]Department of Civil Engineering, Monash University, Clayton, VIC 3800, Australia

*Corresponding author: Hongzhi Cui; Email: h.z.cui@szu.edu.cn

The PDF file includes:

Supplementary Note 1 to 8
Supplementary Figure 1 to 28
Supplementary Table 1 to 3

Supplementary Note 1. Theoretical calculation of the net cooling power and stagnation temperature of the dual-protective coating

Considering a horizontal surface at temperature T_s exposed to the sky with ambient temperature T_{amb}, the net cooling power of the surface P_{net} is then given by:

$$P_{net}(T_s) = P_{rad}(T_s) - P_{atm}(T_{amb}) - P_{sun} - P_{con+\mathrm{cov}} \tag{1}$$

P_{rad} in equation (4) is the power radiated by the surface, which can be calculated from

$$P_{rad}(T_s) = \int_{\cap}\int_0^{\infty}\left[\varepsilon_s(\lambda,\theta)I_{BB}(\lambda,T_s)\right]d\lambda\cos\theta d\Omega \tag{2}$$

where $\int_{\cap} d\Omega = \int_0^{\pi/2}\sin\theta d\theta\int_0^{2\pi}d\phi$ is the hemispherical angular integral, and $\varepsilon_s(\lambda,\theta)$ is the spectral and angular emissivity of the cooler surface. $I_{BB}(\lambda, T_s) = \frac{2hc^2}{\lambda^5}\frac{1}{e^{hc/(\lambda k_B T_s)}-1}$ indicates the spectral radiance of a blackbody, where h is Planck's constant, c is the speed of light in vacuum, and k_B is the Boltzmann constant.

The radiation power from the atmosphere absorbed by the surface $P_{atm}(T_{amb})$ can be expressed as:

$$P_{atm}(T_{amb}) = \int_{\cap}\varepsilon_s(\lambda,\theta)\int_0^{\infty}\left[\varepsilon_{atm}(\lambda,\theta)I_{BB}(\lambda,T_{amb})\right]d\lambda\cos\theta d\Omega \tag{3}$$

According to Kirchhoff's law, under thermal equilibrium conditions, the absorptivity value at a given wavelength and direction equals to the emissivity value for the same wavelength and direction. Thus, in equation (3), the surface's absorptivity $\alpha_s(\lambda,\theta)$ is replaced with the emissivity of the atmosphere $\varepsilon_{atm}(\lambda,\theta) = 1-(1-\varepsilon_{atm}(\lambda,0))^{1/\cos\theta}$, where $\varepsilon_{atm}(\lambda,0)$ is the emissivity in the zenith direction.

The absorbed solar power P_{sun} in equation (1) is given by:

$$P_{sun} = \int_0^{\infty}\left[\varepsilon_s(\lambda,\theta_{sun})I_{AM1.5}(\lambda)\right]d\lambda \tag{4}$$

where $I_{AM1.5}$ represents the AM1.5 solar irradiance.

The non-radiative heat power can be calculated as follows:

$$P_{con+\mathrm{cov}} = h_c(T_{amb} - T_s) \tag{5}$$

Supplementary Note 2. Definition of the average solar reflectivity and thermal emissivity

According to the measured solar reflectivity (R_{solar}) and thermal emissivity ($\varepsilon_{\mathrm{MIR}}$), the average $\overline{R}_{\mathrm{solar}}$ and $\bar{\varepsilon}_{\mathrm{MIR}}$ can be calculated using the following equations:

$$\bar{R}_{solar}=\frac{\int_{0.3\ \mu m}^{2.5\ \mu m} I_{AM1.5}(\lambda)R(\lambda)d\lambda}{\int_{0.3\ \mu m}^{2.5\ \mu m} I_{AM1.5}(\lambda)d\lambda} \quad (6)$$

and

$$\bar{\varepsilon}_{MIR}=\frac{\int_{8\ \mu m}^{13\ \mu m} I_{BB}(\lambda, T)\varepsilon(\lambda)d\lambda}{\int_{8\ \mu m}^{13\ \mu m} I_{BB}(\lambda, T)d\lambda} \quad (7)$$

where λ is the wavelength, $I_{AM1.5}(\lambda)$ is the AM 1.5 global solar intensity and $R(\lambda)$ is the surface solar reflectivity at wavelength λ. Besides, $I_{BB}(\lambda, T)=\frac{2hc^2}{\lambda^5}\frac{1}{e^{hc/(\lambda k_B T)}-1}$ is the spectral radiance of a blackbody at temperature T and wavelength λ, where h is Planck's constant, c is the speed of light in vacuum and k_B is the Boltzmann constant.

$\varepsilon(\lambda)$ is the surface thermal emissivity at wavelength λ, which is calculated via following equation:

$$\varepsilon(\lambda) = A(\lambda) = 1 - R(\lambda) - T(\lambda) \quad (8)$$

where $A(\lambda)$ is the absorptivity and $T(\lambda)$ is the transmittance.

Supplementary Note 3. Cost analysis of the T-PAC

Price and cost are crucial factors for real-world implementation of T-PAC in the construction market. The preparation of T-PAC utilizes widely available, mature, low-cost raw materials (γ-C_2S, $BaSO_4$, SiO_2). Among these, $BaSO_4$—widely employed in industries such as radiative cooling, paper, cosmetics, and medical imaging—is particularly economical, with nanoparticle prices around \$0.17 kg^{-1} [a]. Hydrophobic SiO_2 nanoparticles, though priced higher at \$4.2 kg^{-1} [b], contribute critically to the coating's water-repellent performance. Although γ-C_2S currently represents the primary cost contributor at \$4.93 kg^{-1} [c], its price is anticipated to decrease substantially with scaled production using established synthetic routes[1-3]. Based on this, a preliminary material cost calculation yields approximately \$2.71/m^2, positioning T-PAC as a highly competitive alternative to commercial marine coatings such as epoxy (\$28.9/m^2) [d] and silane treatments (\$4.38/m^2) [e]—costing only about 62% of the latter. (1 USD = 7.09 RMB). Although C_2S is currently the primary cost driver, its price is expected to decrease significantly with mass production by calcining a $Ca(OH)_2$-SiO_2 mixture at a 2:1 molar ratio[1-3].

[a] $BaSO_4$ price (accessed Nov. 25, 2025): https://detail.1688.com/offer/801022743348.html
[b] SiO_2 price: https://detail.1688.com/offer/908898017077.html

[c] C_2S price: https://detail.1688.com/offer/934908599599.html
[d] Epoxy-based coating price: https://e.tb.cn/h.SDISclFWl5RrmKD?tk=miMlfnd1MTM MF937
[e] Silane impregnating agent price: https://e.tb.cn/h.SxGWfaZTrZ8lFSN?tk=uAPxfn2CmOZ CZ057

Supplementary Note 4. Energy barrier calculations of $CaCO_3$ nucleation with and without $BaSO_4$ nanoparticles

The fundamental difference in the nucleation energy barriers before and after adding barium sulfate ($BaSO_4$) nanoparticles to the pure γ-C_2S system can be rationalized through the lens of Classical Nucleation Theory (CNT), which posits that the energy barrier for forming a stable nucleus is a balance between the energy penalty for creating a new surface and the energy gain from forming the bulk crystal. For homogeneous nucleation in the pure γ-C_2S system, where calcium carbonate ($CaCO_3$) must form spontaneously from the solution, this barrier ($\Delta G_{\_hom}$) is calculated using the formula $\Delta G_{\_hom} = (16\pi\gamma^3\Omega^2) / [3(kT \ln S)^2]$, where γ is the interfacial energy between $CaCO_3$ and the solution (estimated at ~0.1 J/m^2), Ω is the molecular volume of $CaCO_3$ (6.136×10^{-29} m^3), k is Boltzmann's constant, T is the temperature (298 K), and S is the supersaturation (assumed to be 10). Plugging in these values yields a homogeneous nucleation barrier on the order of 7.03×10^{-19} J. The introduction of $BaSO_4$ nanoparticles dramatically alters this scenario by providing a template for heterogeneous nucleation. The barrier for this process ($\Delta G_{\_het}$) is substantially reduced and is given by $\Delta G_{\_het} = \Delta G^*_{\_hom} \times f(\theta)$, where $f(\theta)$ is a function of the contact angle (θ) between the nascent $CaCO_3$ nucleus and the $BaSO_4$ substrate. The exceptional lattice matching between the $BaSO_4$ (barite) surface and the calcite structure implies a very low contact angle; assuming $\theta = 30°$, $f(\theta)$ calculates to a very small value of approximately 0.01275. Consequently, the heterogeneous nucleation barrier plummets to roughly 8.96×10^{-21} J. This calculation demonstrates that the presence of the $BaSO_4$ nanoparticles lowers the kinetic barrier for calcite formation by nearly two orders of magnitude, effectively shifting the crystallization pathway from one dominated by kinetics, which favors the metastable aragonite in the pure system, to one governed by thermodynamics, where the stable calcite phase is overwhelmingly favored. It is crucial to note that while the absolute values of these barriers are sensitive to the precise parameters chosen for γ and θ, the profound relative reduction in the energy barrier is a robust theoretical prediction that quantitatively explains the observed polymorphic phase difference.

Supplementary Note 5. Quantifying the effect of radiative cooling on corrosion rate

5.1 Calculation basis and experimental data

The Arrhenius equation describes the relationship between the reaction rate constant (k) and temperature (T):

$$k = A\exp\left(-\frac{E_a}{RT}\right) \tag{9}$$

where k is the reaction rate constant, A is the pre-exponential factor (related to the collision frequency), E_a is the activation energy for the reaction (we adapted a well-established value of ~40 kJ/mol for cement paste[4]), R is the universal gas constant (8.314 J·mol^{-1}·K^{-1}), T is the absolute temperature in Kelvin (K).

For two different temperatures T_1 (uncoated) and T_2 (coated), the relationship between temperature and corrosion rate follows the Arrhenius equation:

$$\frac{i_{corr}(T_2)}{i_{corr}(T_1)} = \frac{i_0 \cdot \exp\left(-\frac{E_a}{RT_2}\right)}{i_0 \cdot \exp\left(-\frac{E_a}{RT_1}\right)} = \exp\left[-\frac{E_a}{R}\left(\frac{1}{T_2} - \frac{1}{T_1}\right)\right] \tag{10}$$

Our experimental temperature measurements during the peak solar period (11:00-13:00) showed:

- Average ambient temperature: $T_{\text{ambient}} = 26.65°\text{C} = 299.80\ \text{K}$
- Average coated surface temperature: $T_{\text{coated}} = 24.35°\text{C} = 297.50\ \text{K}$ (2.3 °C below ambient)
- Average uncoated concrete temperature: $T_{\text{uncoated}} = 49.55°\text{C} = 322.70\ \text{K}$ (25.2°C above coated surface)

5.2 Calculation Procedure

Calculate the reciprocal temperature difference:

$$\frac{1}{T_{\text{coated}}} = \frac{1}{297.50} = 0.003361\ \text{K}^{-1}$$

$$\frac{1}{T_{\text{uncoated}}} = \frac{1}{322.70} = 0.003099\ \text{K}^{-1}$$

$$\Delta\left(\frac{1}{T}\right) = \frac{1}{T_{\text{coated}}} - \frac{1}{T_{\text{uncoated}}} = 0.003361 - 0.003099 = 0.000262\ \text{K}^{-1}$$

Calculate the exponent term:

$$-\frac{E_a}{R} = -\frac{40000}{8.314} = -4811.6$$

$$\text{Exponent} = -\frac{E_a}{R} \times \Delta\left(\frac{1}{T}\right) = -4811.6 \times 0.000262 = -1.26$$

Calculate the corrosion current density ratio:

$$\frac{i_{\text{corr}}(T_{\text{coated}})}{i_{\text{corr}}(T_{\text{uncoated}})} = \exp(-1.575) = 0.284$$

This means the corrosion current density for the coated sample is only 28.4% of that for the uncoated

concrete. Therefore, the passive cooling effect alone provides a 71.6% reduction in corrosion rate, a result that aligns closely with our measured value of 74.3% (calculated as [i_{corr_OPC} - $i_{corr\_CB\text{-}C_2S}$] / i_{corr_OPC}). The slight discrepancy of 2.7% is primarily due to an additional physical barrier provided by the CB-C_2S layer and, consequently, resulting in an underestimation in the theoretical calculations of the corrosion rate–temperature relationship. This calculation demonstrates that temperature regulation is a pivotal mechanism in the coating's overall anti-corrosion strategy.

Supplementary Note 6. Quantifying the effect of radiative cooling on chloride ion transport

To quantitatively address the multifaceted impact of passive cooling on chloride transport and corrosion processes, we then performed a comprehensive analysis based on established physical models and our experimental data. The key findings are summarized as follows:

6.1 Theoretical derivation of the temperature-dependent diffusion coefficient:

The temperature dependence of the chloride diffusion coefficient follows the Arrhenius-type relationship[5] derived from the transition state theory for ionic transport in porous materials. The fundamental form is:

$$D_{Cl}(T) = D_0 \cdot \exp\left(-\frac{E_{a,diff}}{RT}\right) \tag{11}$$

where D_0 is the pre-exponential factor (m^2/s), $E_{a,diff}$ is the apparent activation energy for chloride diffusion (J/mol), typically ranging from 30–45 kJ/mol in cementitious systems[5].

6.2 Derivation of the relative diffusion ratio:

For two different temperatures T_1 (uncoated) and T_2 (coated), the ratio of diffusion coefficients is derived as follows:

$$\frac{D_{Cl}(T_2)}{D_{Cl}(T_1)} = \frac{D_0 \cdot \exp\left(-\frac{E_{a,diff}}{RT_2}\right)}{D_0 \cdot \exp\left(-\frac{E_{a,diff}}{RT_1}\right)} = \exp\left[-\frac{E_{a,diff}}{R}\left(\frac{1}{T_2} - \frac{1}{T_1}\right)\right] \tag{12}$$

Using our experimental data:

$T_1 = 322.70\ \text{K}$ (49.55°C, uncoated)

$T_2 = 297.50\ \text{K}$ (24.35°C, coated)

$E_{a,diff} = 35\ \text{kJ/mol} = 35{,}000\ \text{J/mol}$ (a conservative situation)

$$\frac{D_{Cl}(T_2)}{D_{Cl}(T_1)} = \exp\left[-\frac{35000}{8.314}\left(\frac{1}{297.50} - \frac{1}{322.70}\right)\right] = \exp(-1.103) \approx 0.332$$

This indicates a 66.8% reduction in the chloride diffusion coefficient due to the temperature reduction, which is quite close to the experimental results (69.05%) of a previous study[5], conducted at similar temperature

conditions.

Supplementary Note 7. Quantifying the effect of radiative cooling on corrosion initiation time

7.1 Derivation from Fick's second law

The time for chloride ions to reach the critical concentration at the steel surface can be estimated by the error function solution to Fick's second law under non-steady-state conditions for a semi-infinite medium[6]:

$$C(x,t) = C_s \cdot [1 - \mathrm{erf}(\frac{x}{2\sqrt{D \cdot t}})] \tag{13}$$

where $C(x,t)$ is the chloride concentration at depth x and time t, C_s is the surface chloride concentration, D is the chloride diffusion coefficient.

The corrosion initiation time t_{init} occurs when $C(x,t)$ at the steel depth $x = d$ equals the critical chloride threshold C_{cr}:

$$\frac{C_{cr}}{C_s} = 1 - \mathrm{erf}(\frac{d}{2\sqrt{D \cdot t_{init}}}) \tag{14}$$

7.2 Derivation of the time ratio

For a fixed depth d and concentration ratio C_{cr}/C_s, the argument of the error function must remain constant. Therefore:

$$\frac{d}{2\sqrt{D_1 \cdot t_{init,1}}} = \frac{d}{2\sqrt{D_2 \cdot t_{init,2}}} \tag{15}$$

The ratio of initiation times for two different conditions is given by:

$$\frac{t_{init,coated}}{t_{init,uncoated}} = \frac{D_{uncoated}}{D_{coated}} \cdot \left(\frac{C_{cr,coated}}{C_{cr,uncoated}}\right)^2 \tag{16}$$

This equation reveals that the extension of service life is proportional to the reduction in the diffusion coefficient and the square of the increase in the critical chloride threshold. Substituting the calculated value of relative diffusion ratio (D_{Cl,T_1} / D_{Cl,T_2} = 3.012), the ratio of initiation times for two different temperatures can be expressed as:

$$\frac{t_{init,coated}}{t_{init,uncoated}} = 3.012 \cdot (\frac{C_{cr,coated}}{C_{cr,uncoated}})^2 \tag{17}$$

7.3 Quantifying the increase in critical chloride threshold ($\frac{C_{cr,coated}}{C_{cr,uncoated}}$)

The critical chloride threshold is also highly temperature-dependent. An empirical relationship states that the threshold decreases by approximately ~36% for every 10 °C increase in temperature[7]. The relationship can be expressed as:

$$\frac{C_{cr,coated}}{C_{cr,uncoated}} = (1.36)^{\Delta T/10} \tag{18}$$

where ΔT is the temperature drop in °C. Based on our temperature results, we have:

$$\frac{C_{cr,coated}}{C_{cr,uncoated}} = (1.36)^{\frac{25.2}{10}} = (1.36)^{2.52} \approx 2.17$$

Therefore, the cooling effect increases the critical chloride threshold by a factor of 2.17.

7.4 Final calculation of initiation time extension

We now combine the two effects in the theoretical model:

$$\frac{t_{init,coated}}{t_{init,uncoated}} = \frac{D_{uncoated}}{D_{coated}} \cdot \left(\frac{C_{cr,coated}}{C_{cr,uncoated}}\right)^2 = 3.012 \times (2.17)^2 = 3.21 \times 4.71 \approx 15.1$$

This indicates that the initiation time is calculated to be extended by approximately 15.1-fold solely due to the passive cooling effect.

Supplementary Note 8. Synergistic analysis of radiative cooling and hydrophobic property on anti-corrosion performance

8.1 Quantitative analysis of individual contributions and synergistic enhancement

To accurately isolate the effects of each functionality, we employed a step-by-step analytical framework, with each step building upon the previous one to quantify the added value of each component.

Table S1. Step-by-step analytical framework

Sample	Functionality	i_{corr} (μA/cm²)	Protection Efficiency*	Purpose in Analysis
**OPC**	None (Baseline)	130.868	0%	Reference for no protection
**C-C_2S**	Dense Barrier (Carbonation)	60.447	53.8%	Base improvement from densification
**CB-C_2S**	Dense Barrier + Radiative Cooling	33.644	74.3%	Quantify added value of radiative cooling
**T-PAC**	Full System (All components)	1.453	98.9%	Quantify total synergy

*Protection Efficiency (PE) = [1 - ($i_{corr, sample}$ / $i_{corr, OPC}$)] × 100%

8.1.1 Establishing the baseline improvement (C-C_2S *vs*. OPC):

First, the carbonation (C-C_2S) itself provides a baseline improvement, reducing i_{corr} by approximately 54% compared to OPC. This establishes the "base" upon which the innovative functionalities are added.

8.1.2 Quantifying the individual contribution of radiative cooling (CB-C_2S *vs*. C-C_2S):

The effect of adding radiative cooling to the dense barrier is calculated by comparing CB-C_2S to C-C_2S:

- i_{corr} reduction: 60.447 - 33.644 = 26.803 μA/cm²
- Contribution of radiative cooling: 26.803 / 60.447 ≈ 44.3% reduction relative to the dense barrier baseline.

8.1.3 Quantifying the individual contribution of superhydrophobic barrier (T-PAC *vs*. CB-C_2S):

We assess the effect of adding the superhydrophobic barrier to the "Barrier + Cooling" system (CB-C_2S) by comparing it to the full T-PAC system.

- i_{corr} reduction: 33.644 - 1.453 = 32.191 μA/cm²
- Net Effect of Superhydrophobicity: 32.191 / 33.644 ≈ 95.7% reduction relative to the "Barrier + Cooling" baseline.

8.1.4 Quantifying the overall synergy

If the effects were purely multiplicative, the theoretical i_{corr} of T-PAC would be:

i_{corr}, theoretical = 130.868 × (1 - 53.8%) × (1 - 44.3%) × (1 - 95.7%) ≈ 2.94 μA/cm²

The actual measured i_{corr} of the full T-PAC system is 1.453 μA/cm^2, which is 50.6% lower than this theoretical multiplicative value, demonstrating that the combination achieves substantially better performance than predicted by simple additive models.

We then quantify the synergy between radiative cooling and superhydrophobic barrier using a Synergy Index (SI, a probability model for independent events) calculated as:

$$SI = (PE\_{combined}) / (PE\_{cooling} + PE\_{barrier} - PE\_{cooling} \times PE\_{barrier})$$

Where $PE\_{barrier}$ represents the estimated protection efficiency of the superhydrophobic barrier alone. Based on the dramatic performance improvement observed when individually adding the superhydrophobic barrier or the radiative cooling functionality to the system, we estimate the synergy index to be:

SI = 98.9% / (44.3% + 95.7% - 44.3% × 95.7%) ≈ 1.013

An SI > 1 quantitatively demonstrates that the two functions are not merely independent but interact synergistically, producing a combined effect that surpasses the theoretical prediction. Our calculated SI of 1.013 provides concrete evidence for this positive synergy, which we attribute to a mutual protection mechanisms described below.

8.2 In-depth analysis of the synergistic mechanism

The synergy is not merely additive but mutually protective and enhancing, operating through the following mechanisms:

8.2.1 Cooling-augmented barrier durability:

The radiative cooling layer does more than just slow down kinetics. By maintaining a sub-ambient surface temperature, it reduces the thermal stress on the hydrophobic SiO_2 overlayer. This mitigates thermos-degradation (e.g., oxidation, cracking), which are common failure modes for nano-composite coatings. Furthermore, the sub-ambient cooling effect suppresses surface condensation, particularly during high-humidity nights, which is crucial for maintaining the air-trapping Cassie-Baxter state. This helps prevent an irreversible transition to the wet Wenzel state and reduces damage from cyclic evaporation-condensation process, such as salt crystallization, thereby preserving the integrity of the delicate micro-nano structure essential for long-term superhydrophobicity

8.2.2 Barrier-protected cooling efficiency:

The superhydrophobic layer plays an active role in this synergy. It acts as an impermeable frontline barrier, preventing the pore blockage by seawater and the adhesion of contaminants. This protection is crucial for maintaining the underlying radiative cooling layer's high solar reflectance (94.6%) and thermal emissivity over the long term. The anti-wetting and self-cleaning capability are keys, as a fouled or wet surface would suffer from plummeting reflectance and a consequent dramatic increase in solar absorption, which would severely compromise cooling performance. Our tests (Fig. 4c) corroborate this mechanism, showing that even after forced wetting, the coating's reflectance recovers to >91% upon drying, demonstrating the self-cleaning effect working in concert with cooling.

8.2.3 Multi-stage corrosion suppression:

The synergy provides comprehensive defense through complementary mechanisms (Figure S26):

- Stage 1 (Initiation): The superhydrophobic layer is the primary defense, physically blocking >90% of chloride ingress (Fig. 3h).
- Stage 2 (Diffusion): The cooling function provides secondary defense by reducing the chloride diffusion coefficient by ~66.8%, dramatically delaying the time for any residual chlorides to reach the steel surface.
- Stage 3 (Propagation): Once chlorides breach the barrier, the cooling layer becomes the primary defense, reducing the corrosion reaction rate by ~74.3% via Arrhenius kinetics suppression (Fig. 5h).

The superhydrophobic layer acts as a primary shield, drastically reducing initial chloride ingress. The radiative cooling function then serves as a powerful secondary defense, significantly slowing the diffusion

and electrochemical reaction kinetics of any residual chlorides, their combination successfully mitigates the primary failure modes of each individual function, leading to a system whose overall performance is greater than the sum of its parts, achieving near-complete corrosion suppression.

Supplementary Figures

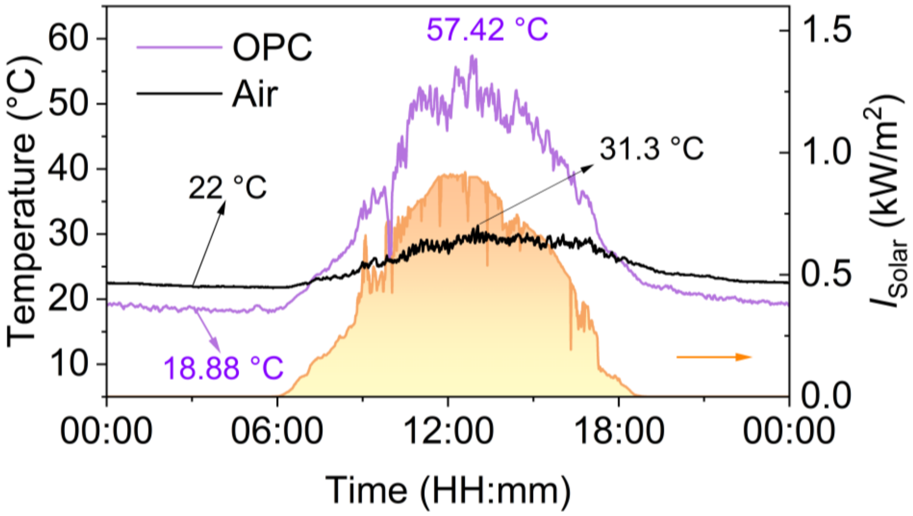

Figure S1. Time-dependent temperature variation of ordinary Portland cement under direct sunlight irradiation. On a summer day in ShenZhen City, the measured temperature fluctuates from18.9 to 57.4 °C in a diurnal cycle. This figure clearly shows the temperature difference between the concrete surface and the ambient environment under solar radiation. Source data are provided as a Source Data file.

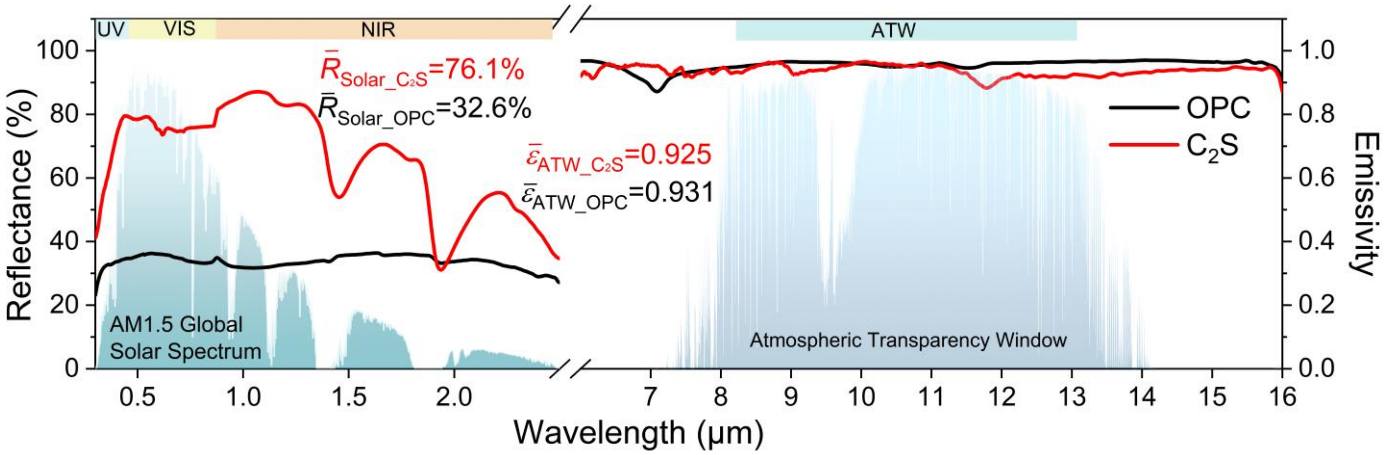

Figure S2. Solar reflectance and LWIR emittance of C_2S and ordinary Portland cement. In the solar spectrum, C_2S is more reflective than ordinary Portland cement. In the mid-infrared spectrum, both ordinary Portland cement and ordinary Portland cement are intensively emissive owing to the molecule vibration of Si-O-Si (8.3 - 12.5 μm). Source data are provided as a Source Data file.

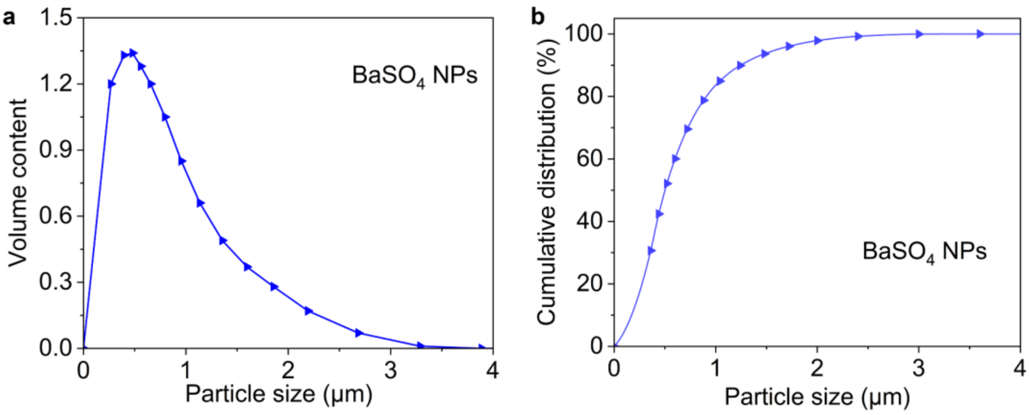

Figure S3. Size distribution of $BaSO_4$ NPs measured by dynamic light scattering. The $BaSO_4$ NPs display a broad size distribution from ~0.3 μm to 3.5 μm with an average particle size of ~0.48 μm. Source data are provided as a Source Data file.

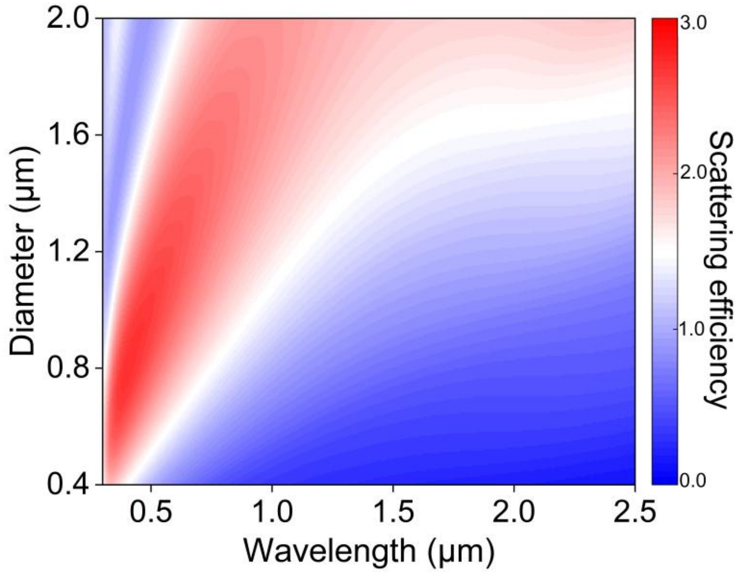

Figure S4. Simulated scattering efficiency of $BaSO_4$ of different particle sizes. The results show that nanoscale $BaSO_4$ particles effectively scatter light in the UV-Vis wavelength range, while microscale $BaSO_4$ particles extend this capability into the NIR region. Source data are provided as a Source Data file.

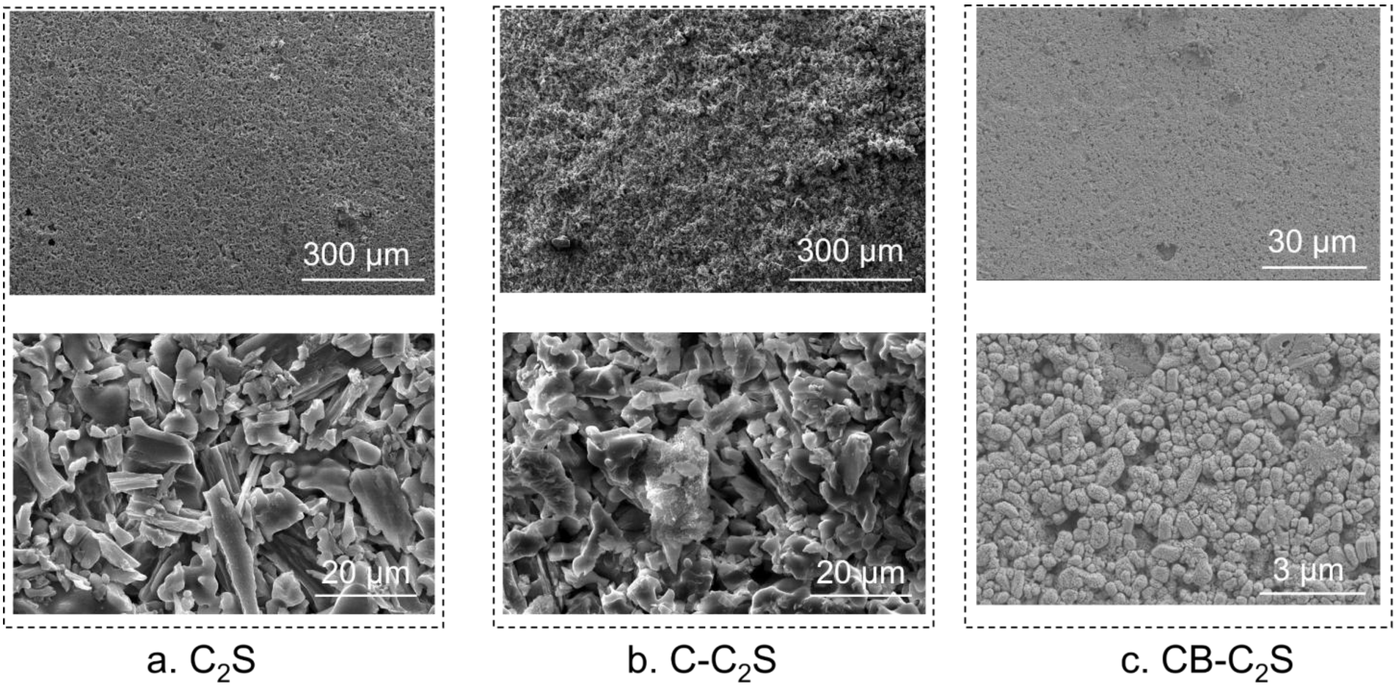

Figure S5. Surface micromorphology of (a) C_2S, (b) C-C_2S, and (c) CB-C_2S. The pristine C_2S coating exhibits flat morphology with substantial micro-voids randomly distributed on the surface. At higher magnification, numerous irregular column-like particles can be visualized on the pristine C_2S coating, which are randomly stacked or partially connected by gel-like hydration products on the surface, forming a hierarchical porous structure that is favorable for sunlight scattering and air accommodation. After carbonation treatment, the original smooth surface of pristine C_2S coating is transformed into an uneven surface with improved roughness and a denser microstructure, formed by continuous calcium carbonates encapsulating the unreacted particles and silica gels. When $BaSO_4$ NPs are blended with the carbonated C_2S, the microstructure of the coating surface becomes denser.

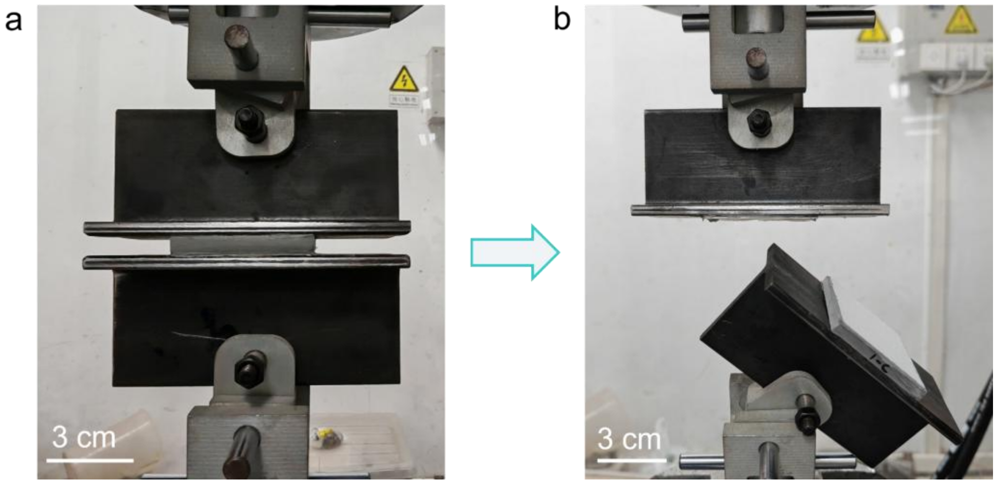

Figure S6. Measurement of interfacial bonding strength between T-PAC and OPC. a To evaluate the interfacial bonding strength, we performed a perpendicular pull test using a sandwich specimen, in which a layer of T-PAC was sandwiched between an OPC layer and a high-stiff resin layer. The specimen is prepared by inlaying a layer of T-PAC on a high-stiff resin layer, followed by casting a layer of OPC slurry on the surface of T-PAC. **b** After the OPC is completely hardened, the specimen is tested by a universal material testing machine under a loading rate of 0.02 mm/min. The debonding occurs at the interface between WPC

and resin, yielding a peak strength of ~1.61 MPa.

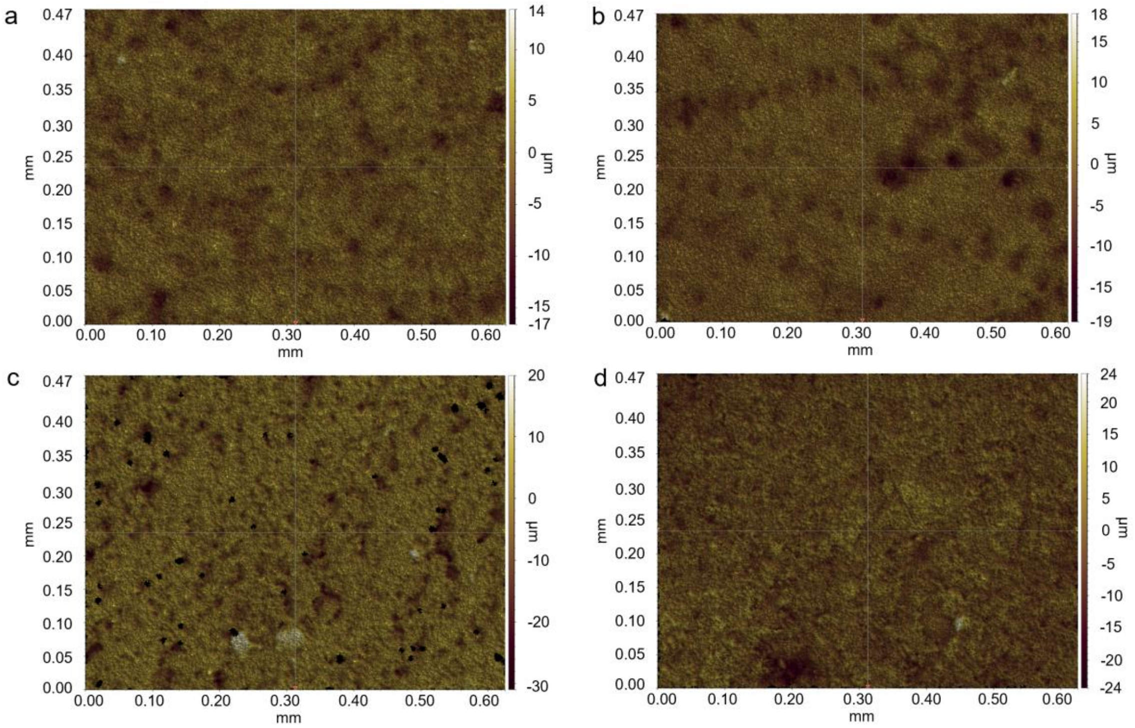

Figure S7. Multiscale surface roughness of the C_2S derived coatings on OPC substrate. a C_2S, **b** C-C_2S, **c** CB-C_2S, and **d** T-PAC. Pristine C_2S coatings showed microscale surface roughness that was moderately enhanced by carbonation. Synergistic carbonation with $BaSO_4$ nanoparticles further coarsened the surface topography relative to carbonation alone, whereas additional SiO_2 nanoparticle deposition reduced surface irregularities.

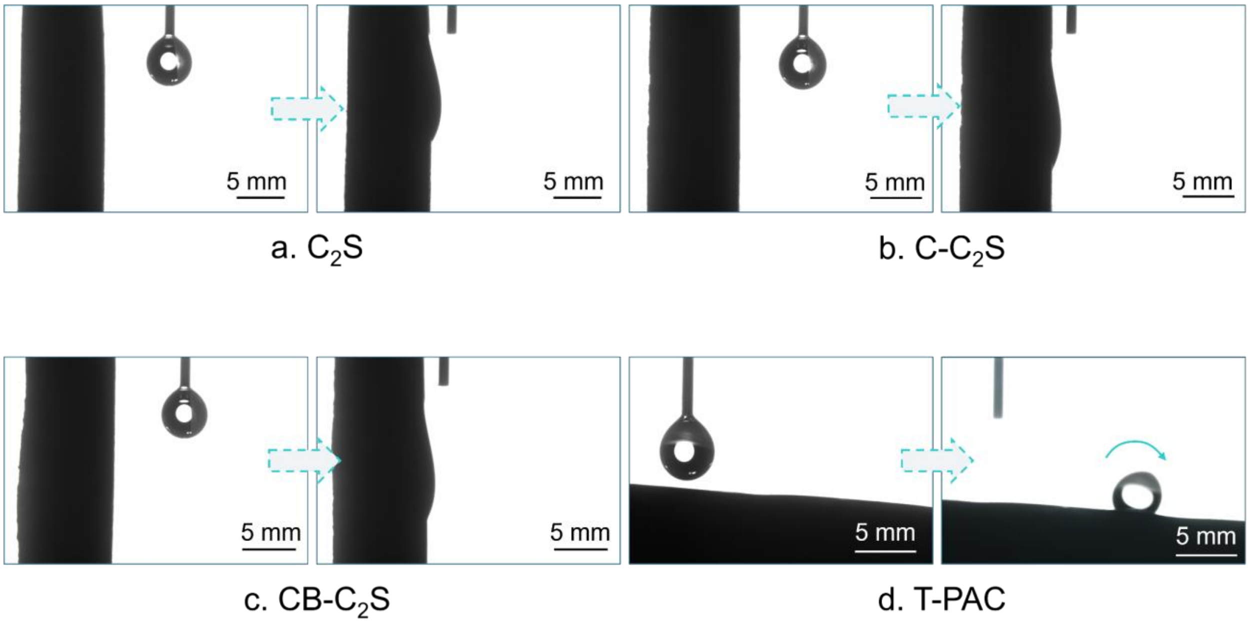

Figure S8. Roll-off angle measurements on inclined surfaces. The coating samples without hydrophobic overlayer undergo complete absorption upon droplet contact, failing to shed droplets gravitationally even at inclinations of 90° (Figure S8a-c), whereas the hydrophobically optimized T-PAC coating enables gravity-driven droplet detachment at angles below 5° (Figure S8d).

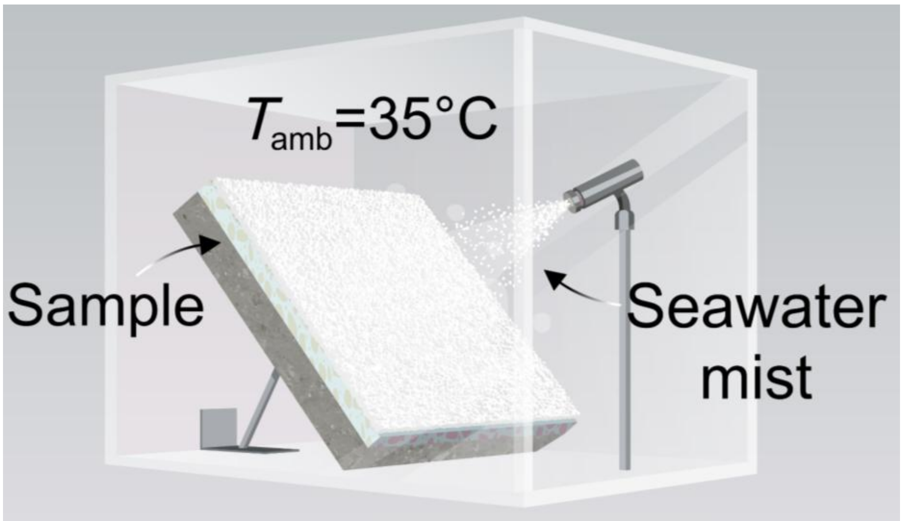

Figure S9. Dynamic seawater droplet repellency in coastal-simulated complex environments. The evaluation was conducted in coastal-simulated complex environments involving multiple droplets, high humidity (RH≈100%) and elevated temperature (35°C) using 3.5 wt.% NaCl solution.

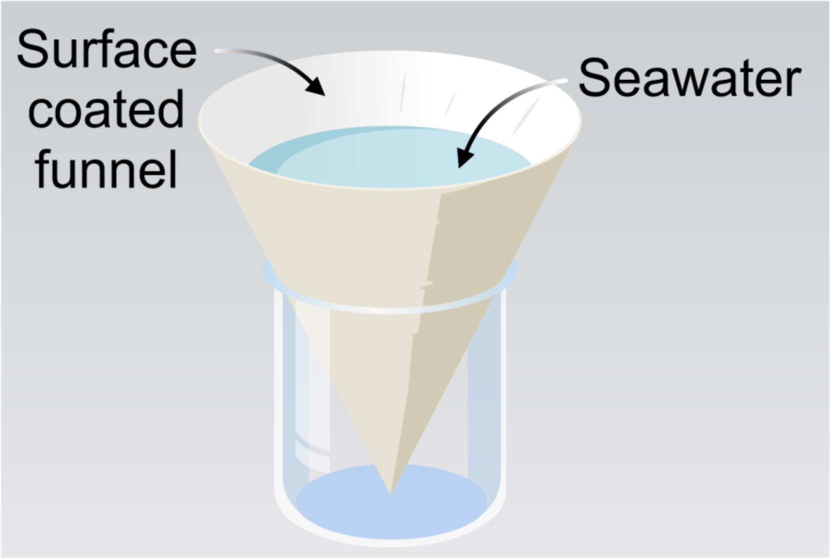

Figure S10. Evaluation of coatings' ability to block the ingression of Cl^- ions. The corrosive ion-blocking capabilities of different C_2S-derived coatings were evaluated by filtering seawater through porous films coated with various C_2S derived coatings.

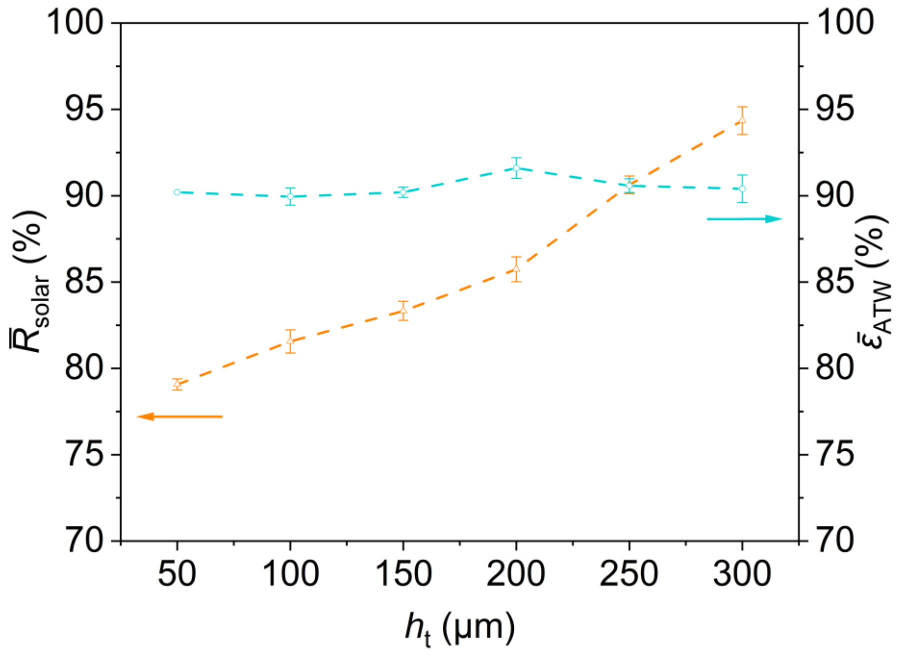

Figure S11. Measured $\bar{R}_{solar}$ and $\bar{\varepsilon}_{ATW}$ of the T-PAC in response to the coating thickness. The error bars represent the standard deviation, calculated based on three replicate experiments. Source data are provided as a Source Data file.

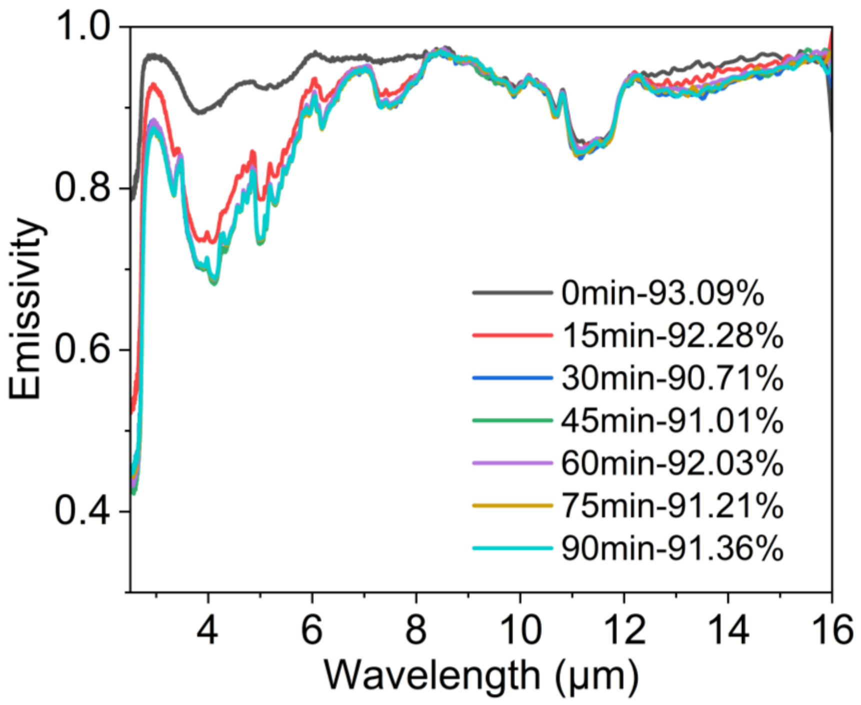

Figure S12. $\bar{\varepsilon}_{ATW}$ variation of the water-wetted T-PAC when losing superhydrophobic properties. As the absorbed seawater evaporates, the $\bar{\varepsilon}_{ATW}$ of the T-PAC only experienced slight variation between 93.09%~91.36%. Source data are provided as a Source Data file.

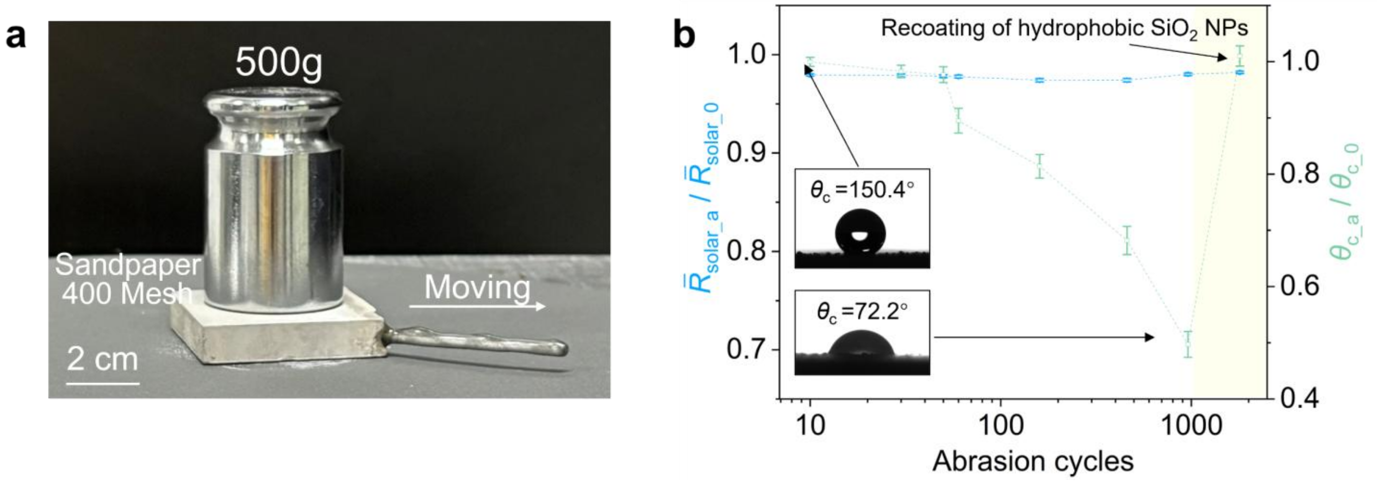

Figure S13. Abrasion tolerance test T-PAC. a The experimental configuration for the abrasion tolerance test. **b** The variation of solar reflectance and water repellence of T-PAC under different abrasion cycles. Inserted figures show the water droplet profile at the first 10 abrasion cycles and 1,000 abrasion cycles. The light-yellow shading highlights the recovery of superhydrophobicity after recoating SiO_2 NPs. The error bars in Fig. S13**b** represent the standard deviation, calculated based on three replicate experiments. Source data are provided as a Source Data file.

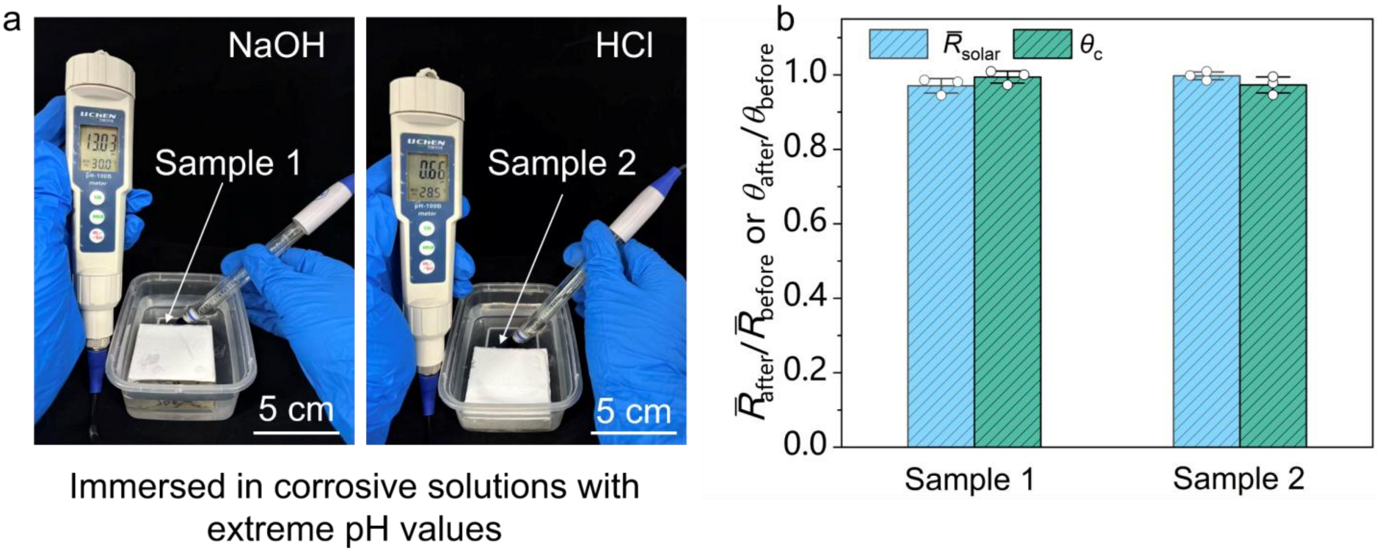

Figure S14. Corrosion resistance test of T-PAC. a Corrosion resistance test of two identical T-PAC samples in corrosive solutions (NaOH solution with a pH of 13.03 and HCl solution with a pH of 0.66). **b** Results of solar reflectance and water repellence of T-PAC after being immersed in the corrosive solutions for 15 minutes. The error bars in Fig. S14**b** represent the standard deviation, calculated based on three replicate experiments. Source data are provided as a Source Data file.

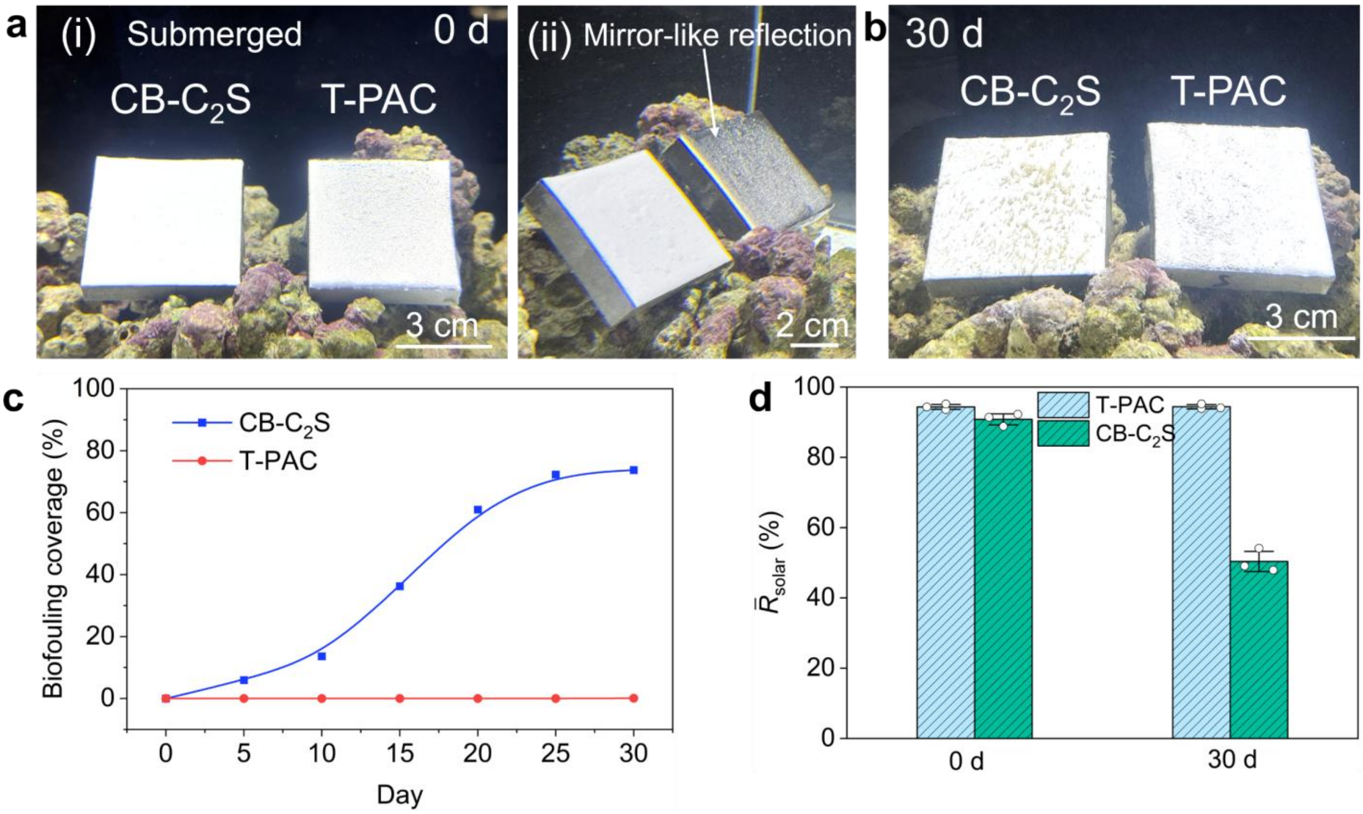

Figure S15. Anti-biofouling test of T-PAC and control sample without superhydrophobic protection (CB-C_2S). **a** Initial surface conditions of T-PAC and CB-C_2S in a submerged environment. **b** Surface morphology of T-PAC and CB-C_2S after 30 days of simulated seawater immersion. **c** Biofouling coverage of T-PAC and CB-C_2S in response to different immersion durations. **d** Measured $\bar{R}_{\text{solar}}$ of T-PAC and CB-C_2S before and after 30 days of seawater immersion. The error bars in Fig. S15**d** represent the standard deviation, calculated based on three replicate experiments. Source data are provided as a Source Data file.

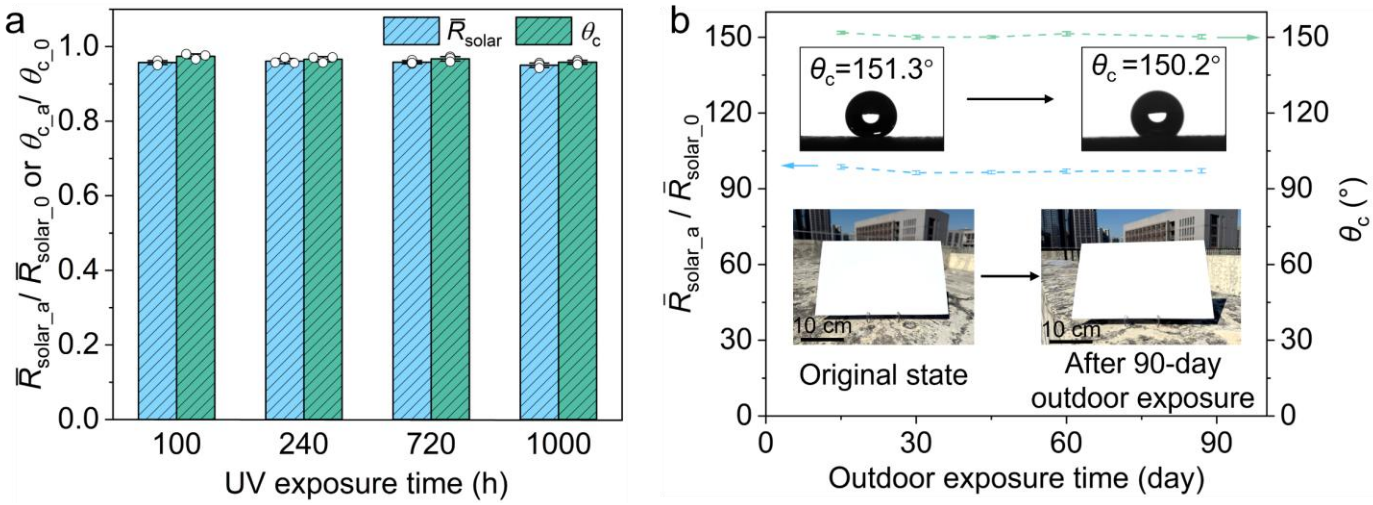

Figure S16. UV aging and weather resistance test. Solar reflectance and water contact angle of the T-PAC measured at different UV exposure times (**a**) and different outdoor exposure times (**b**). The error bars in Fig. S16**a** represent the standard deviation, calculated based on three replicate experiments. Source data are provided as a Source Data file.

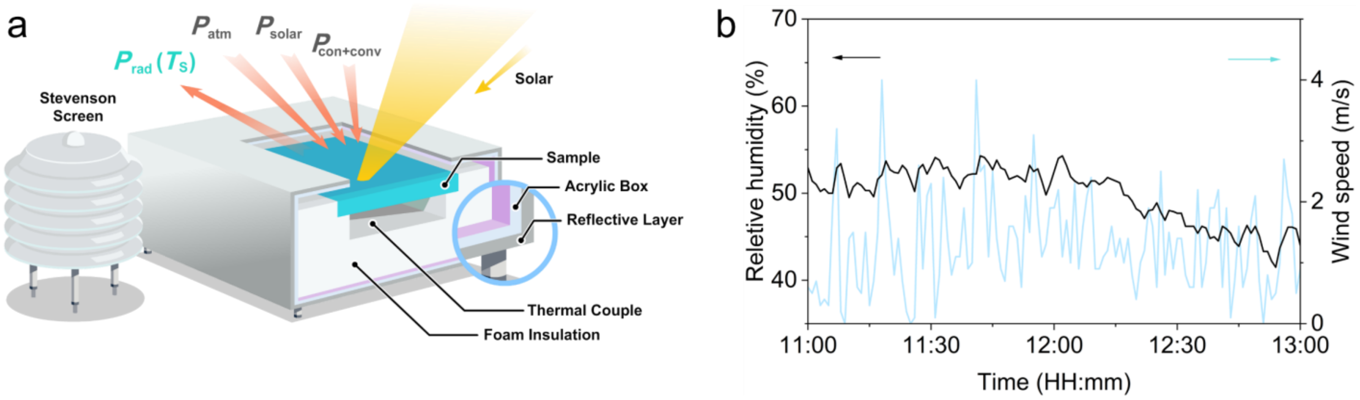

Figure S17. Experimental setup and environmental parameters of outdoor test. a Schematic of the test system used to monitor the real-time temperature variation of the experimental samples. **b** The recorded environmental parameters during the hottest period (11:00~13:00) of a day. Source data are provided as a Source Data file.

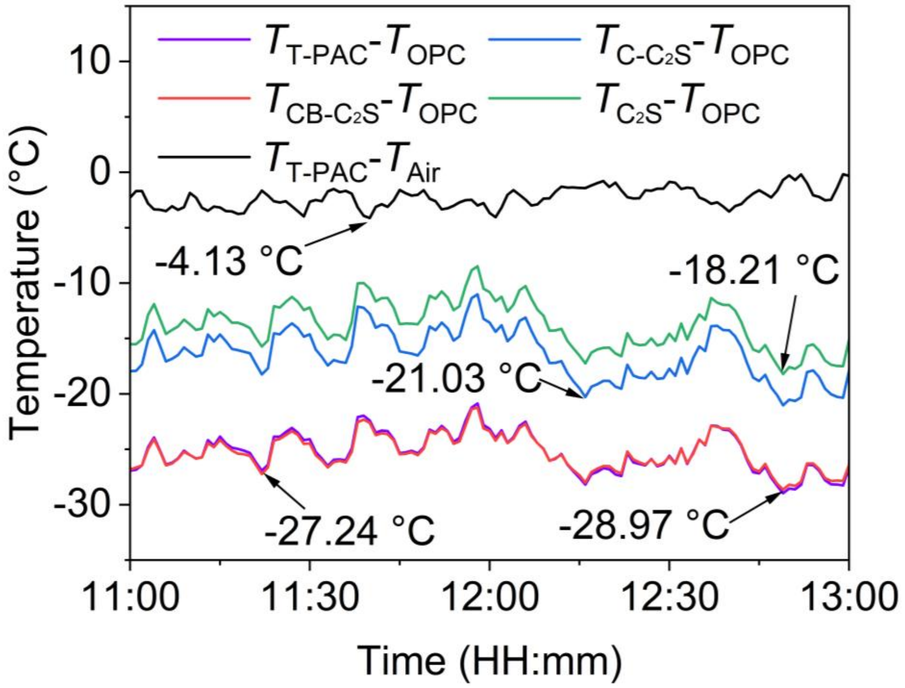

Figure S18. Calculation of temperature difference between the C_2S-derived coatings. In the hottest period of a sunny day (11:00–13:00), the CB-C_2S and T-PAC samples achieved an average temperature reduction of approximately 25.2 °C compared to the OPC sample, with a peak reduction of 28.97 °C. In contrast, the C-C_2S and C_2S samples exhibited a less pronounced cooling effect, reaching peak temperature reductions of 21.03 °C and 18.21 °C, respectively. Source data are provided as a Source Data file.

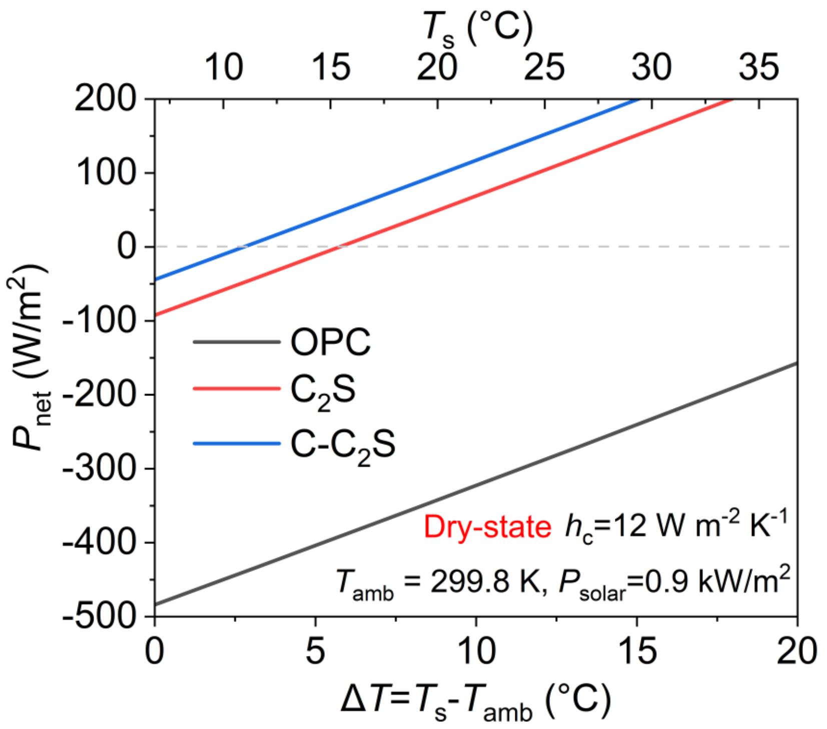

Figure S19. Net cooling power calculations. Calculations of P_{net} values from C-C_2S (-44.3 W/m^2), C_2S (-92.4 W/m^2), and OPC (-484 W/m^2) under non-radiative heat transfer coefficient of 12 Wm^{-2} K^{-1} and peak solar intensity of 900 W/m^2. The dash line represents lowest net cooling power. Source data are provided as a Source Data file.

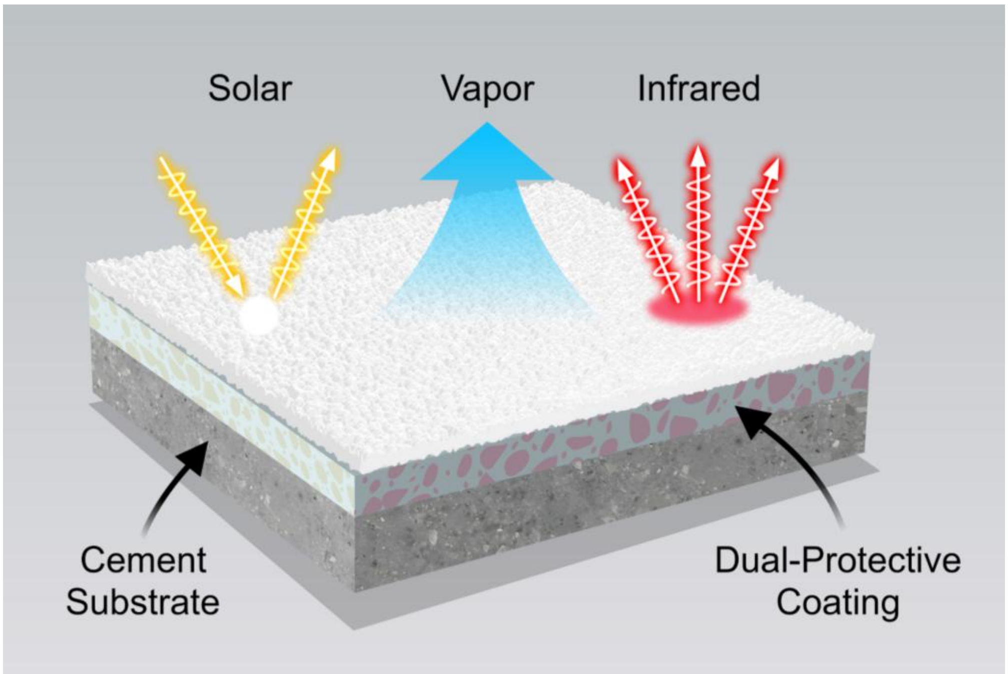

Figure S20. Schematic diagram showing the heat transfer of a water-wetted T-PAC sample. For a water-wetted T-PAC sample, the heat transfer process involves thermal input from solar radiation, thermal dissipation enabled by infrared emission, and non-radiative thermal loss from water evaporation.

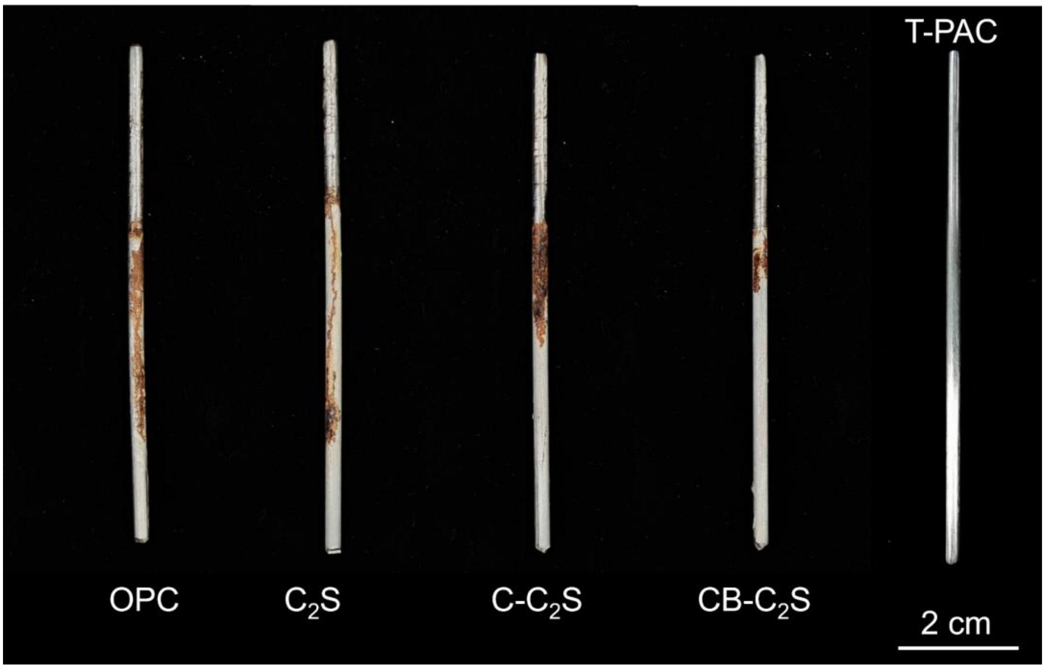

Figure S21. Photographs showing the corrosion area of the C_2S derived coating protected samples and control sample. The T-PAC-coated sample exhibited an intact surface morphology with no visible corrosion, validating the synergistic protective effect of its integrated radiative cooling and superhydrophobic layers. By contrast, the OPC, C_2S, C-C_2S, and even CB-C_2S samples showed heavily degraded, rough surfaces with filamentous rust, indicative of active, non-uniform corrosion due to the lack of effective protection.

.

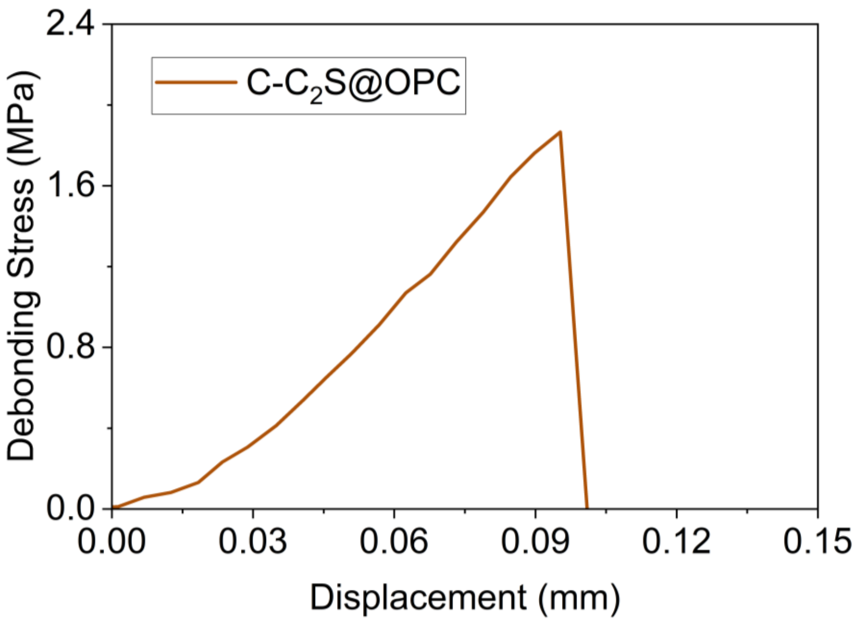

Figure S22. Interfacial bonding strength between C-C_2S and OPC substrate. The chemical compatibility between carbonated C_2S endows it with an improved interfacial debonding strength of 1.86 MPa with the OPC substrate. Source data are provided as a Source Data file.

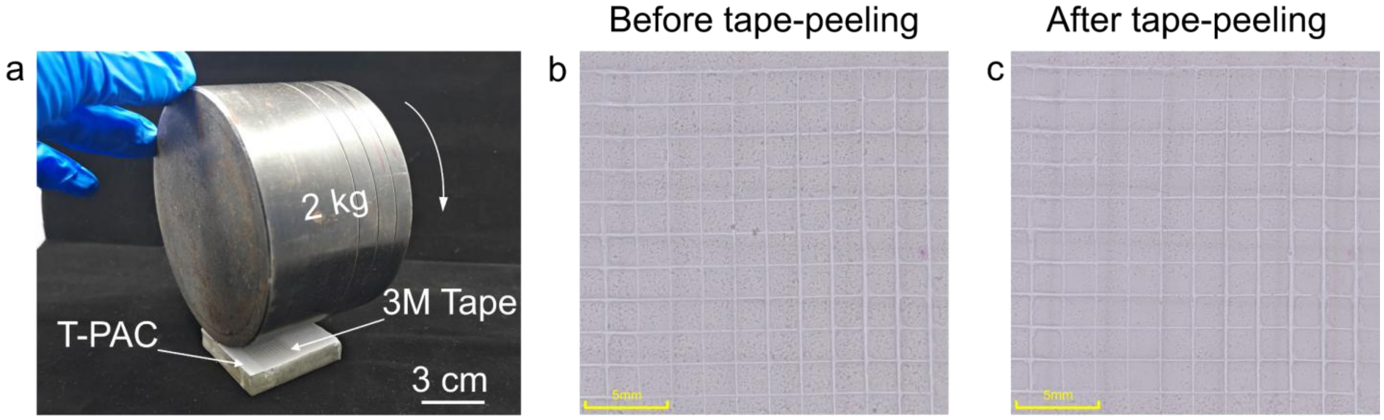

Figure S23. Tape-peeling test of T-PAC. a Photography of the tape-peeling test. **b** Surface of the T-PAC after being treated by the multiple tap cutters. **c** Surface of the T-PAC after tape-peeling test. The T-PAC only shows several detachment sites and maintains superior structural integrity after being peeled off using 3M tape under a uniform load of 2 kg, which is an indicator of adequate interfacial adhesion between the superhydrophobic SiO_2 overlayer and the carbonated gel substrate.

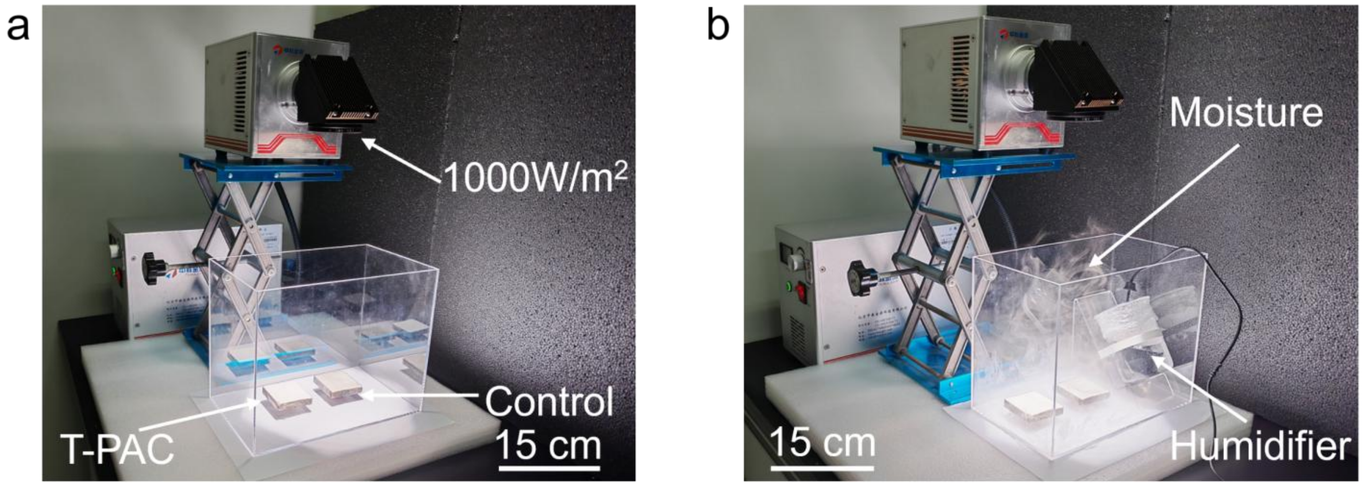

Figure S24. **Photograph of experimental setup for durability test under high humidity and 1000W/m^2 solar radiation. a** Experimental setup of continuous irradiation at ambient humidity for T-PAC and a control (superhydrophobic but solar absorptive OPC) sample. The two samples are sealed by epoxy with only one surface directly exposed to the air. **b** Experimental setup of continuous irradiation at high humidity for T-PAC and the control sample. The humidity is increased by a commercial humidifier.

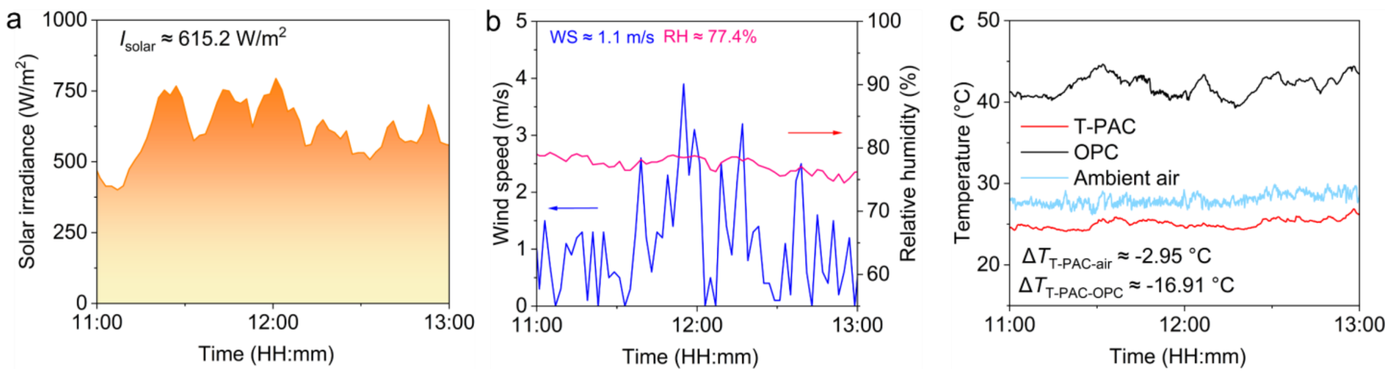

Figure S25. **Outdoor cooling performance of the T-PAC under a high relative humidity of 77.4%. a** Solar

irradiance recorded during the hottest period. **b** Wind speed and relative humidity during the outdoor measurement. **c** Temperature variation of T-PAC, OPC, and ambient air during the tested period. Source data are provided as a Source Data file.

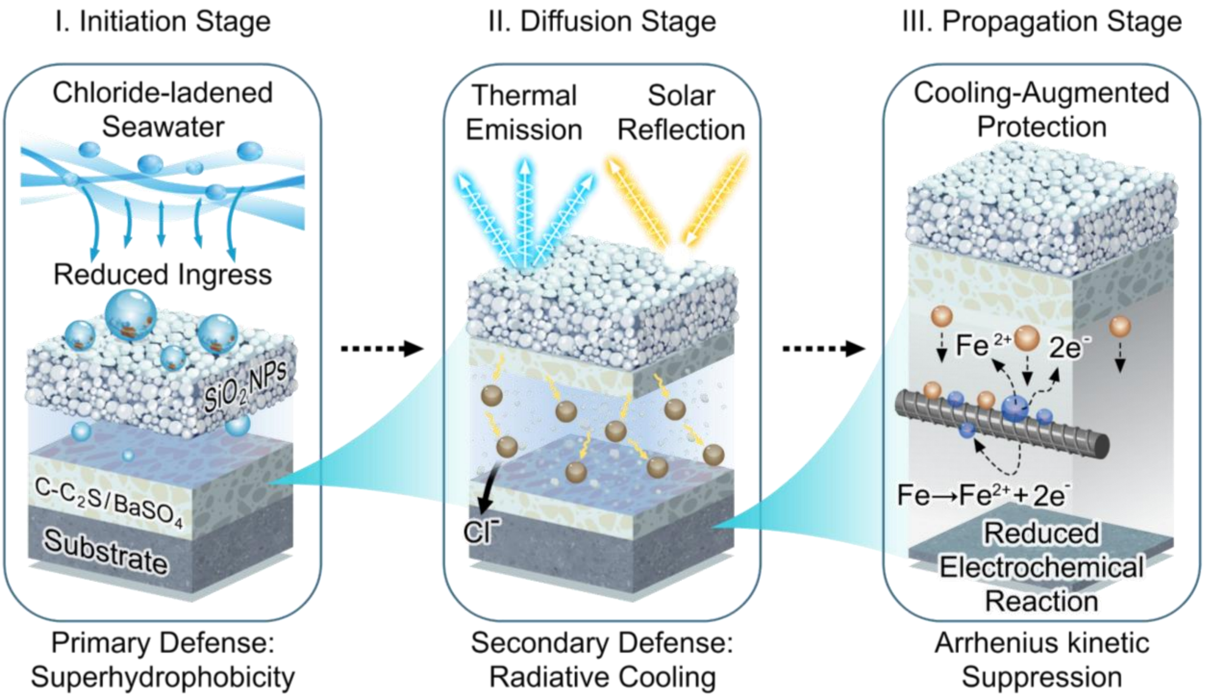

Figure S26. **Schematic diagram showing the multi-stage corrosion protection mechanism of T-PAC.** The superhydrophobic layer acts as a primary shield, drastically reducing initial chloride ingress. The radiative cooling function then serves as a powerful secondary defense, significantly slowing the diffusion and electrochemical reaction kinetics of any residual chlorides, their combination successfully mitigates the primary failure modes of each individual function, leading to a system whose overall performance is greater than the sum of its parts, achieving near-complete corrosion suppression.

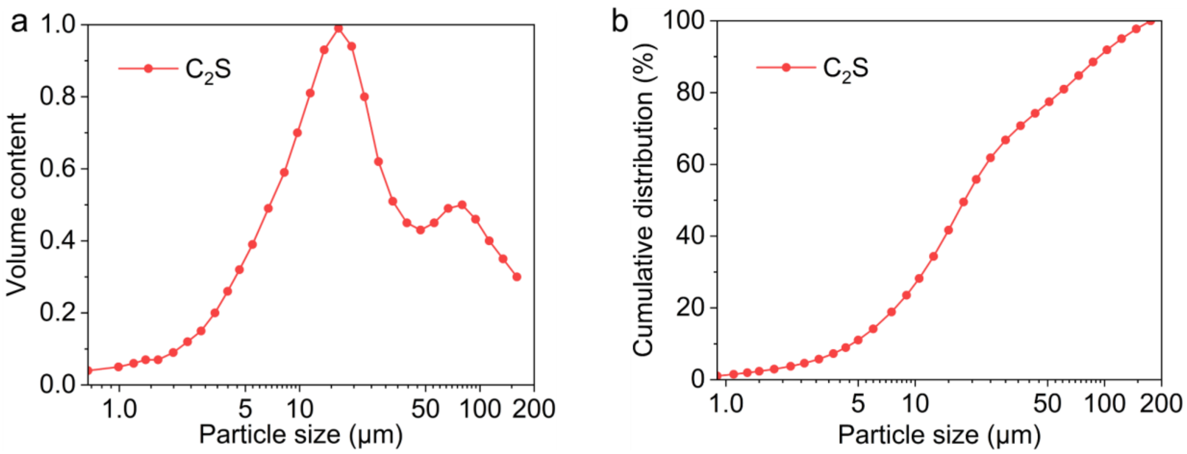

Figure S27. Particle size distribution analysis of the pristine C_2S powder. The results obtained from a laser particle size analyzer show the continuous and homogeneous particle size distribution of C_2S with a median diameter of 15 μm. Source data are provided as a Source Data file.

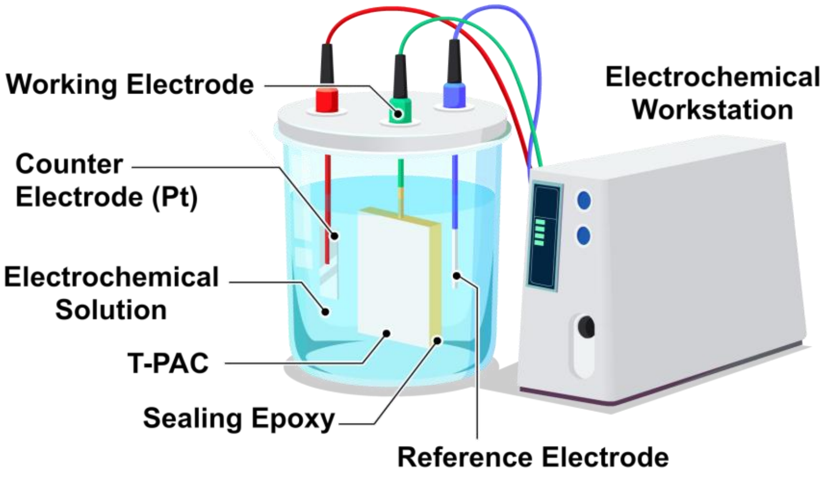

Figure S28. Schematic diagram showing sample illustration and the setup for the electrochemical tests in this study. The experiment was conducted using an electrochemical workstation coupled with a three-electrode cell under steady open-circuit voltage, where the working electrode was a two-end-sealed Q235 steel bar (Ø0.3 cm × 7 cm) partially embedded in a coated sample (50 mm × 50 mm × 10 mm), while a platinum electrode and a saturated calomel electrode served as the counter and reference electrodes, respectively.

Supplementary Table

Table S2. Chemical composition of C_2S

CaO	SiO_2	MgO	SrO	Au	Fe_2O_3	Re_2O_7	SO_3	P	TiO_2	GeO_2
65.22	34.34	0.374	0.019	0.0128	0.0124	0.0079	0.0067	0.0032	0.0026	0.0019

Table S2. Chemical analysis of C_2S powder. The chemical components of C_2S are tested by X-ray fluorescence spectroscopy. The results mainly show the weight ratio of common metal oxides in the cementitious materials, such as CaO, SiO_2, and others. Particularly, the content of solar-absorptive Fe_2O_3 in C_2S is extremely low, which endows it with pure whiteness as compared to the gray appearance of OPC.

Table S3. Comparing T-PAC with established coating systems for marine concrete protection.

Coating system	Protection Mechanism	Key Advantages	Key Limitations/Drawbacks
**This work: T-PAC**	**1. Passive Radiative Cooling:** Reduces surface temperature, suppressing Arrhenius kinetics. **2. Superhydrophobic Barrier:** Blocks chloride-laden water ingress. **3. Enhanced Bonding:** Silica gel ensures integral bonding.	**Multifunctional:** Simultaneously mitigates thermal and chemical corrosion drivers. **Energy-free & Passive:** Requires zero operational energy. **Chemically Compatible:** Seamless integration with concrete substrate. **Carbon-Negative Process:** Carbonation sequesters CO_2. **Durable Adhesion:** Cementitious bonding eliminates delamination risk.	**Application Specific:** Optimal for hot, solar-exposed climates.
**Epoxy / Polyurethane**	**1. Physical Barrier:** Dense film isolates substrate.	**Proven Technology:** Widely available and specified. **High Mechanical Strength:** Excellent abrasion resistance.	**Single-Function:** Only a chloride barrier; ignores thermal acceleration. **Poor UV/Heat Resistance:** Prone to chalking, yellowing, and degradation. **Adhesion Failure:** Thermo-mechanical mismatch with concrete causes delamination. **Environmental Concern:** Derived from petrochemicals.
**Silane Treatments**	**1. Pore Linings:** Hydrophobizes capillary pores.	**Deep Penetration:** Treats the substrate bulk. **Vapor Permeable**	**Single-Function:** Only a water repellent; no thermal regulation. **Durability Concerns:** Limited service life;
**Cathodic Protection**	**1. Electrochemical Intervention:** Forces steel to be cathodic.	**Highly Effective:** Can stop ongoing corrosion.	**Energy-Intensive:** Requires continuous power supply and monitoring. **High Maintenance:** Complex and costly over a full lifecycle. **Not a Preventive Coating:** An active system, not a passive material solution.

Table S3. Comparing T-PAC with established coating systems for marine concrete protection. As detailed in the table, our T-PAC coating establishes a new paradigm by moving beyond single-mechanism protection. Unlike epoxy/polyurethane coatings, which are susceptible to UV degradation[8] and delamination due to their polymeric nature and thermomechanical mismatch with concrete[9], the T-PAC's inorganic, cementitious matrix forms a compatible and robust interface (Figure 2g), ensuring long-term durability. Compared to pore-lining treatments such as silanes, which provide only hydrophobic functionality, our coating introduces a crucial second protective dimension: passive radiative cooling. This dual-functionality simultaneously suppresses the Arrhenius-type kinetic acceleration of corrosion (Figure 5h) by maintaining sub-ambient temperatures while also providing an excellent barrier against capillary seawater absorption (Figure 3g, f). This positions the T-PAC system as a comprehensive solution to the coupled thermo-chemo degradation inherent to marine environments. Most significantly, our approach provides a passive, zero-energy alternative to active systems like cathodic protection, eliminating the need for continuous energy input and sophisticated monitoring. This makes it particularly suitable for large-scale, remote marine infrastructure where maintenance and energy costs are prohibitive. Another crucial advantage of T-PAC lies in its sustainability, in which the use of carbonation-activated C_2S as a binder is a carbon-negative process, aligning with global decarbonization goals, whereas the production of organic coatings like epoxy carries a significant carbon footprint and petrochemicals. Thus, the T-PAC coating is a comprehensive solution that synergistically integrates multiple protective functions—thermal regulation, ion-blocking, and enhanced bonding—into a single, sustainable, energy-free anti-corrosion system, offering a comprehensive solution for enhancing the durability of marine concrete structures under coupled thermo-chemo environmental stress.

References

1 Chen, J., Zhao, M., Liu, Z., Wang, F. & Hu, S. Bioinspired in situ synthesis of high-strength bulk CO_2 mineralized ceramics at room temperature. *ACS Nano* **19**, 1732-1742 (2025).

2 Chen, J., Liu, Z., Zhang, S., Hu, S. & Wang, F. Energy-efficient fabrication of biomimetic materials for sustainable infrastructure applications. *Adv. Sci.* **12**, e03854 (2025).

3 Zhao, S., Liu, Z., Wang, F., Hu, S. & Liu, C. Effect of extended carbonation curing on the properties of γ-C_2S compacts and its implications on the multi-step reaction mechanism. *ACS Sustain. Chem. Eng.* **9**, 6673-6684 (2021).

4 Shafei, B., Alipour, A. & Shinozuka, M. Prediction of corrosion initiation in reinforced concrete members subjected to environmental stressors: A finite-element framework. *Cem. Concr. Res.* **42**, 365-376 (2012).

5 Page, C. L., Short, N. R. & El Tarras, A. Diffusion of chloride ions in hardened cement pastes. *Cem. Concr. Res.* **11**, 395-406 (1981).

6 Collepardi, M., Marcialis, A. & Turriziani, R. Penetration of chloride ions into cement pastes and concretes. *J. Am. Ceram. Soc.* **55**, 534-535 (1972).

7 Alhozaimy, A. M., Ahmed, M., Hussain, R. R. & Al Negheimish, A. Quantitative non-linear effect of high ambient temperature on chloride threshold value for steel reinforcement corrosion in concrete under extreme boundary conditions. *Materials* **14**, 7595 (2021).

8 Zhang, X. Metamaterials for perpetual cooling at large scales. *Science* **355**, 1023-1024 (2017).

9 Yan, X., Yang, M., Duan, W. & Cui, H. Particle–solid transition architecture for efficient passive building cooling. *ACS Nano* **18**, 27752-27763 (2024).
