# Supplementary material for: Dual-path suppression of thermal and wetting-driven steel corrosion for marine structure
Source: Nat Commun. 2026 Apr 21;17:5471. doi: 10.1038/s41467-026-71930-x (PMC13284360; doi:10.1038/s41467-026-71930-x)
Supplement: Supplementary file 2 — Transparent Peer Review file [file 41467_2026_71930_MOESM2_ESM.pdf]

nature portfolio

Peer Review File

Dual-Path Suppression of Thermal and Wetting-Driven Steel Corrosion for Marine Structure

Corresponding Author: Professor Hongzhi CUI

Parts of this Peer Review File have been redacted as indicated to remove third-party material.

This file contains all reviewer reports in order by version, followed by all author rebuttals in order by version.

Version 0:

Reviewer comments:

Reviewer #1

(Remarks to the Author)
In marine environment, chloride-induced corrosion of steel bars is one of the main causes for the deterioration of RC structures. Among the present corrosion prevention/protection methods, surface coating/treatment on concrete is cost-effective due to easy application, low cost and high barrier effect. Radiative cooling coatings are now widely studied for the building energy conservation and temperature regulation. Multi-functional radiative cooling coatings have been developed recently, including the superhydrophobic and self-cleaning functions to enhance the performance of temperature regulation. However, solar reflective coatings are rarely considered to be applied in the marine structures for corrosion protection. It is a potential way to address the influence of temperature on acceleration of chloride induced corrosion. Based on this, this work adopts radiative cooling technology combined with superhydrophobic functions to establish a dual-path-suppression coating system for enhancing the durability of concrete structures in marine environments. The concept of temperature regulation to mitigate steel corrosion in concrete is interesting and necessary, while anti-wetting by superhydrophobicity is a common path to inhibit chloride-induced corrosion in marine environment.
There are some key issues present in this work, which need further clarification and strengthening in terms of the mechanism demonstration, verification of long-term durability, connection with practical engineering applications, and specifically quantification of the exact role of temperature regulation in corrosion control.
1. In the Introduction, the detailed review of the passive cooling technology on concrete should be added to present the research progress and clarify the novelty of the passive cooling coating in this work. $BaSO_4$ and hydrophobic SiO_2 are commonly used in passive cooling coating and superhydrophobic coatings, and cementitious materials have already been studied to develop the passive cooling coating. A simple combination of passive cooling and superhydrophobic technology for the anti-corrosion doesn't seem so innovative.
2. The synthesis process of C2S/$BaSO_4$ composites coating should be reorganized to clearly present the details of preparation. The time for the $BaSO_4$ nanoparticle incorporation, the ratio of $BaSO_4$ NPs to deionized water during the ultrasonication, the method to control the amount of superhydrophobic SiO_2 NPs (10 wt.% of total solids) should be clarified. What is the carbonation duration for Control 2 and 3 samples? The SiO_2 NPs were sprayed on the carbonated gel surface (water-mixed), which is not reasonable due to the superhydrophobic properties. Is the gel fresh when spraying the SiO_2 NPs? How to ensure the adhesion performance between two layers?
3. For the proposed composite coating, the hydration or gelation mechanism, the phase assemblage, the microstructure and the adhesion mechanism between different layers are all critical for the analysis of the anti-corrosion performance. The lack of this information limited the depth of mechanism demonstration in this work.
4. The experimental program for the anti-corrosion performance should present the sample illustration and the setup for the electrochemical tests. The information of concrete type and cover thickness, sealing of the steel end, coating thickness et al. are all important for the analysis of the test results. According to Figure S17, the surface conditions of the steel bars are quite different, and it will influence the corrosion initiation induced by chloride ion. The corrosion morphology and the much lower initial Z modulus of OPC, C2S and C-C2S samples are strange, and detailed analysis is needed. There seems no passivation in concrete.
5. A temperature-wetting mediated passive anti-corrosion coating is proposed in this work. One of the highlights in this work may lie in the suppression efficiency of passive cooling performance of the coating on the corrosion behavior of steel in concrete, and to what degree the temperature regulation affects the corrosion behavior. To quantify the effect of passive cooling performance on the transport of chloride ion, the corrosion initiation and the corrosion rate might be critical issues to be addressed in this work. The analysis of corrosion behavior is superficial and lacks synergy and quantitative contribution analysis. The frame of the work should be reorganized to emphasize this highlight.

6. The anti-wetting and anti-corrosion experiments are mainly based on short term tests (no longer than 30 days). The long-term durability of C2S/BaSO4 composites coating steel needs to be verified. The physiochemical properties are also suggested to be studied, including the UV aging, abrasion resistance, weathering resistance, chemical stability and even the biofouling. If available, even preliminary data from a field exposure test (e.g., in a splash zone) would greatly enhance the impact of the manuscript.
7. Passive cooling coating is expected to exhibit dual effects both for energy conservation and also the corrosion inhibition in marine environment in this work. Comparison with that of standard marine coatings (e.g., epoxy, polyurethane, silane treatments) is of great significance. Include a brief discussion on the scalability, application cost, repairability, and limitations of the coating system, which are crucial for assessing its practical translational potential. Actually, passive cooling coatings are suitable for hot climate environments, while superhydrophobic coatings are not suitable for long-term humid or underwater environments.
8. The statement "During the heat transfer equilibrium, heat gain from the solar irradiation causes instability of passive films on steel rebars due to reduced critical chloride threshold required for depassivation under elevated temperatures" in line 129 is not logical for the knowledge of chloride induced corrosion. In Figure S1, if available, the ambient temperature in Shenzhen should be added.

Reviewer #2

(Remarks to the Author)
This manuscript presents a novel and interesting study on a multifunctional coating system that innovatively addresses the coupled thermo-wetting degradation mechanisms in marine concrete structures. The authors demonstrate remarkable insight by integrating radiative cooling physics with superhydrophobic materials science to develop a dual-barrier coating (T-PAC) that simultaneously suppresses both Arrhenius-type kinetic acceleration and chloride ion penetration. The work is scientifically rigorous, featuring multi-scale characterization—spanning spectral analysis, wetting/thermal performance validation under simulated environmental conditions, electrochemical corrosion monitoring and sophisticated interpretation of results through coupled heat-mass transfer modelling. The coating's passive operation, cementitious compatibility, and scalability further enhance its practical relevance. While the study makes a significant contribution to the field, a few minor revisions could further strengthen its clarity and impact.
This paper is recommended for publication in Nature Communications after addressing the following comments:
1. Except for C2S, C3S also exhibits carbonation reactivity and chemical compatibility with concrete surface, why do the authors choose C2S as the binder instead of C3S? A comparative discussion on potential benefits would provide valuable insights into this choice.
2. The abbreviation "T-PAC" (temperature-wetting mediated passive anti-corrosion coating) is introduced in both the Introduction and Results sections; however, terms such as "thermal-wetting" or "thermo-wetting" are inconsistently used elsewhere in the manuscript. Please use consistent description for better reader accessibility.
3. Reasons for choosing BaSO4 NPs over other dielectric particles (e.g., Al2O3, TiO2, CaCO3, or h-BN) as the functional filler for improving solar reflectance and mid-infrared emittance should be provided.
4. In line 73, the authors refer to "high solar reflectance (0.3~2.5 μm)", but, as a rule, spectral measurements are carried out in the range of 0.28~2.5 μm. The authors should unify the wavelength range of the solar band throughout the main text.
5. In line 75, the term "coastal concrete" is used, but elsewhere in the manuscript, "coastal concrete structure" appears to be the intended term. Please check the manuscript for the same problem.
6. Lines 422-423: In the outdoor test, the authors note that the outdoor test was conducted without a wind shelter, which differs from methodologies used in other studies. Please elaborate on this choice.

Version 1:

Reviewer comments:

Reviewer #1

(Remarks to the Author)
The authors performed additional experiments and refined the main content of the work. All the concerns with the manuscript have been addressed. The revised manuscript can be accepted for publication.

Reviewer #2

(Remarks to the Author)
With the incorporation of the revisions, the manuscript has achieved a high level of innovation and rigor. In its current form, I believe it is suitable for publication.

Open Access This Peer Review File is licensed under a Creative Commons Attribution 4.0 International License, which permits use, sharing, adaptation, distribution and reproduction in any medium or format, as long as you give appropriate credit to the original author(s) and the source, provide a link to the Creative Commons license, and indicate if changes were made.
In cases where reviewers are anonymous, credit should be given to 'Anonymous Referee' and the source.
The images or other third party material in this Peer Review File are included in the article's Creative Commons license, unless indicated otherwise in a credit line to the material. If material is not included in the article's Creative Commons license and your intended use is not permitted by statutory regulation or exceeds the permitted use, you will need to obtain permission directly from the copyright holder.
To view a copy of this license, visit https://creativecommons.org/licenses/by/4.0/

Point-by-point response to the reviewers' comments

Dear Editor and Reviewers:

The authors would like to thank the Editor and Reviewers for their review of our work and the constructive comments, which are very helpful in improving the quality of the manuscript. We have studied every comment carefully and incorporated them as suggested. Revised portions are highlighted in the revised manuscript with blue remarks and responses to the reviewers' comments are shown as below:

REVIEWER COMMENTS

Reviewer #1 (Remarks to the Author):

In marine environment, chloride-induced corrosion of steel bars is one of the main causes for the deterioration of RC structures. Among the present corrosion prevention/protection methods, surface coating/treatment on concrete is cost-effective due to easy application, low cost and high barrier effect. Radiative cooling coatings are now widely studied for the building energy conservation and temperature regulation. Multi-functional radiative cooling coatings have been developed recently, including the superhydrophobic and self-cleaning functions to enhance the performance of temperature regulation. However, solar reflective coatings are rarely considered to be applied in the marine structures for corrosion protection. It is a potential way to address the influence of temperature on acceleration of chloride induced corrosion. Based on this, this work adopts radiative cooling technology combined with superhydrophobic functions to establish a dual-path-suppression coating system for enhancing the durability of concrete structures in marine environments. The concept of temperature regulation to mitigate steel corrosion in concrete is interesting and necessary, while anti-wetting by superhydrophobicity is a common path to inhibit chloride-induced corrosion in marine environment.

There are some key issues present in this work, which need further clarification and strengthening in terms of the mechanism demonstration (1), verification of long-term durability (2), connection with practical engineering applications (3), and specifically quantification of the exact role of temperature regulation in corrosion control (4).

Response: We sincerely thank the reviewer for the positive assessment of the concept and for the constructive comments.

1. In the Introduction, the detailed review of the passive cooling technology on concrete should be added to present the research progress and clarify the novelty of the passive cooling coating in this work. $BaSO_4$ and hydrophobic SiO_2 are commonly used in passive cooling coating and superhydrophobic coatings, and cementitious materials have already been studied to develop the passive cooling coating. A simple combination of passive cooling and superhydrophobic technology for the anti-corrosion doesn't seem so innovative.

Response: We have expanded the Introduction (Page 3, Lines 78-90) to include research progress on passive cooling coating applied to concrete and to define the specific contributions of the present work.

The contributions lie in the integration of radiative cooling with superhydrophobicity within a carbonation-activated C_2S gel matrix for corrosion inhibition in marine environments. This integration provides:

1) Radiative cooling to reduce temperature-driven acceleration of chloride-induced corrosion in reinforced concrete, rather than solely for energy conservation.
2) Synergistic suppression of chloride transport and corrosion kinetics through combined temperature reduction and wetting resistance.
3) Mutual protection between the radiative cooling and superhydrophobic layers (Figure R1).

[Figure Redacted]

Figure R1. Schematic diagram showing the mutual protection mechanism of radiative cooling and superhydrophobic property in T-PAC. Briefly, the superhydrophobic layer repels seawater and resists biofouling, enabling rapid liquid shedding. This maintains a dry, clean surface that ensures unimpeded solar reflection and mid-infrared emission for efficient cooling. Meanwhile, the radiative cooling layer significantly lowers the surface temperature. This cooling effect substantially stabilizes the three-phase contact line between seawater and the underlying SiO_2 nanoparticles, thereby suppressing surface condensation under high-humidity coastal conditions and facilitating fast recovery of water repellency.

The proposed mutual protection mechanism has been elucidated via two comparative experiments under varied humidities and fixed simulated sunlight (1000 W m^{-2}, Figure R2a, b), which is included in the revised manuscript (Pages 24-25, Lines 747-774). First, the sliding angles of water droplets were measured on T-PAC (with radiative cooling and superhydrophobicity) and a superhydrophobic control sample (without radiative cooling) after each reached thermal steady state under ambient humidity. As shown in Figure R2c, the surface temperature of T-PAC was approximately 18.6 °C lower than that of the control (Figure R2c), and its water droplets slid off at a tilt angle of only ~3° (Figure R2d), compared to ~26.5° for the control (Figure R2e), indicating maintained Cassie–Baxter stability. Subsequently, under sustained irradiation while humidity was raised to >90 % RH, T-PAC exhibited discrete, fine condensation droplets (<1 mm), characteristic of dropwise condensation, whereas the control sample showed coalesced droplets and film-wise condensation (Figure R2f), signaling a transition toward the Wenzel state. These distinct behaviors are attributed to the temperature-dependent stability of the air layer trapped in the surface microstructures. The lower temperature of T-PAC helps preserve a robust air layer that confines liquid contact and vapor condensation to the texture tops, thereby

sustaining a non-wetting state and dropwise condensation. Conversely, the elevated temperature of the control thermally destabilizes this air layer, promoting vapor penetration, nucleation within the textures, and a local transition to the Wenzel state, which enhances droplet pinning, coalescence, and film formation. Thus, the radiative cooling layer effectively protects the superhydrophobic layer by stabilizing the solid–liquid–air interface, which suppresses the wetting transition and reduces condensation under coupled thermal-humid conditions.

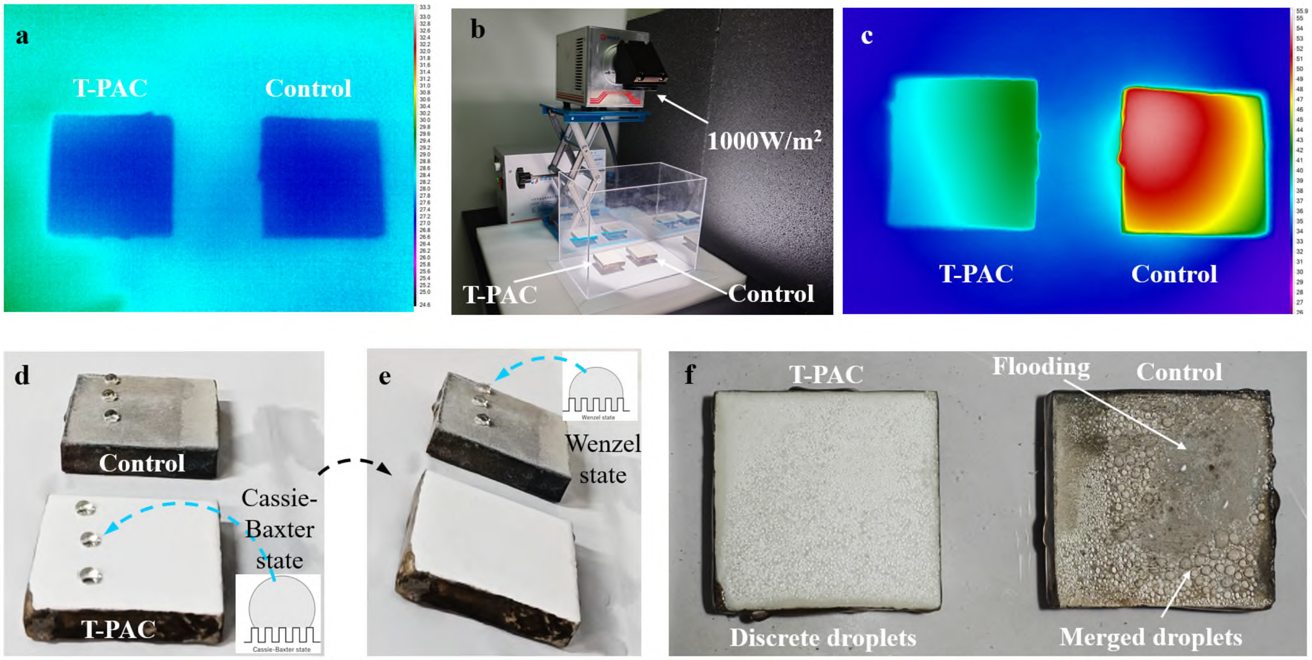

Figure R2. Wettability condensation behaviors of T-PAC (combining radiative cooling and superhydrophobicity) and control superhydrophobic sample. **a** Infrared image showing the temperature of T-PAC and control sample before simulated sunlight illumination. **b** Experimental setups of the simulated sunlight illumination. **c**. Infrared image showing the temperature of T-PAC and control sample after 60 mins simulated sunlight illumination. **d** Droplets on T-PAC begin to slide under a tilting angle of ~3°. **e** Droplets on control sample begin to slide under a tilting angle of ~35°. **f** Condensation behavior of T-PAC and control sample under simulated conditions combining sunlight illumination and high-humidity.

To further elaborate how the superhydrophobic layer protects the thermal regulation performance of the radiative cooling layer, we compared the thermal response of T-PAC and a control sample (radiative cooling only, without superhydrophobicity) before and after contamination with viscous mud slurry. As illustrated in Figure R3a, when viscous mud slurry was dripped on the T-PAC surface, it quickly converged into streams and slid off under gravity, leaving the surface clean and visually unchanged. This self-cleaning behavior stems from the low surface energy and hierarchical roughness of the superhydrophobic layer, which minimizes liquid spreading and adhesion. In contrast, the hydrophilic control sample (CB-C_2S) allowed the mud to spread and adhere across the surface, resulting in substantial coverage by dark residue (Figure R3b). Under irradiation from a xenon lamp (1000 W m^{-2}), T-PAC exhibited stable thermal performance before and after contamination: both the transient temperature profiles and the peak steady-state temperature (~33 °C) remained nearly identical (Figure R3c). This consistency confirms that the superhydrophobic layer effectively preserves optical access to the radiative cooling surface. By comparison, the control sample showed a marked increase in temperature after soiling, rising from ~32.9 °C to ~44.2 °C (Figure R3d), due to the optical shielding caused

by adhered mud. Infrared thermography over a 40-minute test further underscored this protective role. The temperature difference for T-PAC before and after contamination remained below 1.2 °C (Figure R3e), whereas the control sample experienced a temperature rise of ~11.1 °C under the same conditions (Figure R3f). These results demonstrate that the superhydrophobic layer sustains cooling performance through self-cleaning, which prevents fouling and maintains high solar reflectance and MIR emission.

Therefore, our novelty rests on the rational architectural integration within a cementitious system to pioneer a new application (marine corrosion control) based on a novelly articulated multi-physics principle (temperature-wetting mediation) through mutual interplay between radiative cooling and superhydrophobicity. We thank the reviewer again for prompting us to clarify this critical point.

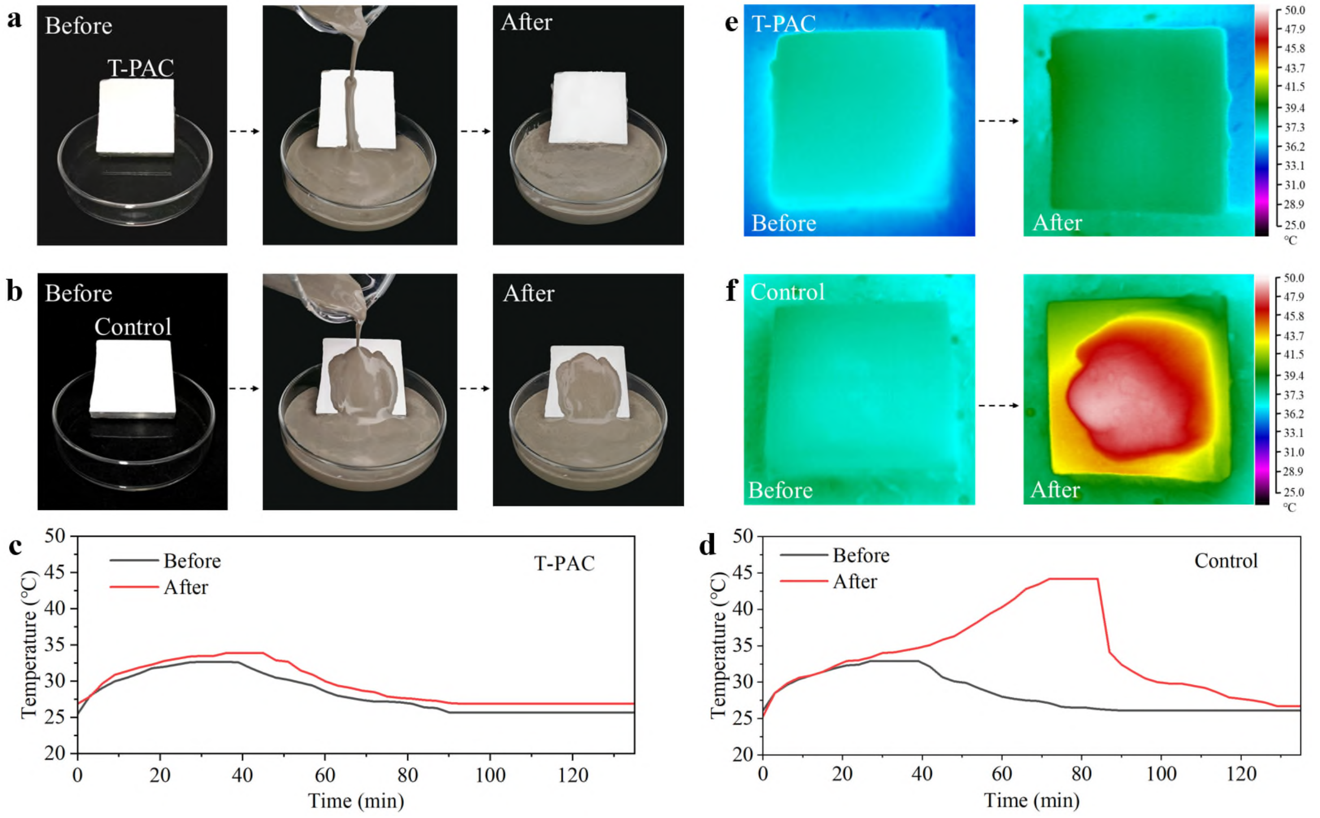

Figure R3. Thermal response of T-PAC and control sample before and after contamination. **a** Contamination test of T-PAC. **b** Contamination test of control. **c** Infrared image showing the temperature of T-PAC before and after contamination treatment. **d** Infrared image showing the temperature of control before and after contamination treatment. **e** Temperature variation of T-PAC before and after contamination treatment. **f** Temperature variation of control before and after contamination treatment.

2. The synthesis process of $C_2S/BaSO_4$ composites coating should be reorganized to clearly present the details of preparation. The time for the $BaSO_4$ nanoparticle incorporation, the ratio of $BaSO_4$ NPs to deionized water during the ultrasonication, the method to control the amount of superhydrophobic SiO_2 NPs (10 wt.% of total solids) should be clarified. What is the carbonation duration for Control 2 and 3 samples? The SiO_2 NPs were sprayed on the carbonated gel surface (water-mixed), which is not reasonable due to the superhydrophobic properties. Is the gel fresh when spraying the SiO_2 NPs? How to ensure the adhesion performance between two layers?

Response: Following the reviewer's suggestion, detailed synthesis parameters have been added to the

Methods section, including the mass ratios for $BaSO_4$ dispersion (1:5, 2.5:5, 5:5 $BaSO_4$ to H_2O), the spraying specifications for the SiO_2 overlayer (10 wt.% in ethanol, 0.3 mm nozzle, 0.2 MPa), and the standardized carbonation duration (30 min at 20% CO_2, 70% RH, 25 °C). The interfacial adhesion between the superhydrophobic SiO_2 layer and the carbonated gel substrate is also mechanistically elucidated.

Detailed additions regarding synthesis parameters can be found in the revised Methods section (Page 28, Lines 848-860 and 866-868):

1) **$BaSO_4$ dispersion**: the mass ratio of $BaSO_4$ to deionized water during the ultrasonication was set at 1:5, 2.5:5, and 5:5. After ultrasonication treatment, the resulting $BaSO_4$ dispersion was immediately incorporated into the mixture of C_2S and water-reducing agent. (Page 28, lines 851-856)

2) **SiO_2 spraying**: a 10 wt.% hydrophobic SiO_2 in ethanol solution was sprayed onto the dry carbonated gel surface using an airbrush (nozzle diameter: 0.3 mm, pressure: 0.2 MPa). The amount of deposited SiO_2 NPs was controlled by varying the spraying time and was quantitatively verified through mass measurement after ethanol evaporation. The underlying carbonated gel before SiO_2 spraying is a dry, rigid hybrid coating composed of silica gel, $BaSO_4$, and $CaCO_3$. (Page 28, lines 859-868)

3) **Carbonation duration**: Controls 2 and 3 samples underwent identical 30 minutes carbonation in a controlled environment (20% CO_2, 70% RH, 25 °C). (Page 28, lines 874-876)

4) **Adhesion performance**: The interfacial adhesion between the superhydrophobic SiO_2 overlayer and the carbonated gel substrate is ensured through a combination of morphological and nanoparticle-engineering strategies. The carbonated gel surface provides ample roughness and hydroxyl groups for nanoparticle anchoring. Additionally, the use of low surface tension ethanol (~22 mN/m) as spraying medium promotes capillary wicking into the gel's pores, ensuring the SiO_2 nanoparticles are firmly compacted and anchored upon solvent evaporation, thereby fostering interfacial locking with the rough, hydroxyl-rich gel substrate.

To underpin the adequate interfacial adhesion between the superhydrophobic SiO_2 overlayer and the carbonated gel substrate, we performed tape-peeling tests (Figure R4a) on T-PAC according to the ASTM 5B standard. Cross-cutting of the sample was performed using a QHF cutting knife, with a spacing of 1 mm between each grid. 100 small grids were firmly affixed to the samples using 3M 600 tape and a 2 kg load was applied to ensure full contact with the tested area. Following a resting period of 90 seconds, the tape was rapidly and vertically pulled off at an angle of 90°. This process was repeated twice on the same area. Subsequently, the remaining coating on the substrate was examined to evaluate the interfacial adhesive strength. Results in Figure R4b, c show that the T-PAC only exhibits several detachment sites and maintains structural integrity after being peeled off using 3M tape under a uniform load of 2 kg, which is an indicator of adequate interfacial adhesion between the superhydrophobic SiO_2 overlayer and the carbonated gel substrate. The result of interfacial adhesion measurement have been included in the supplementary information.

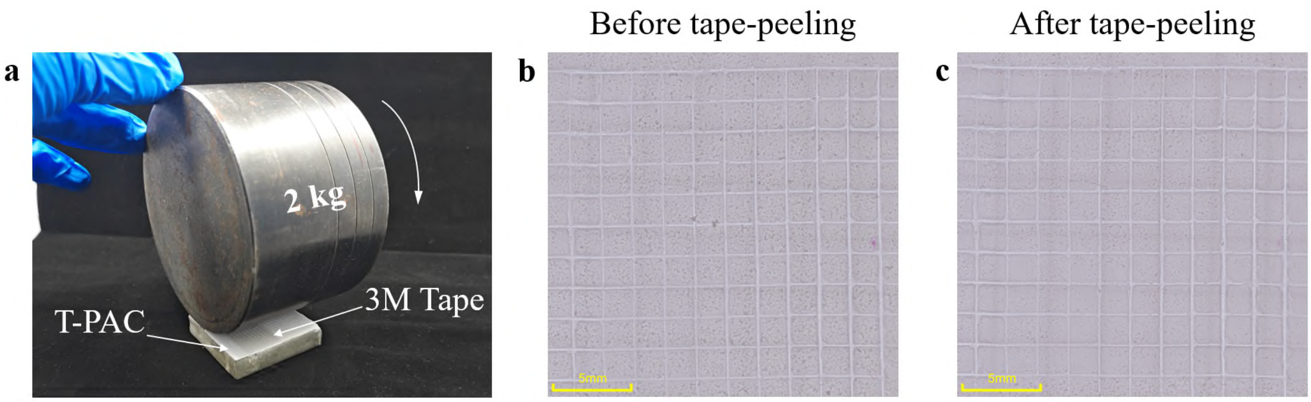

Figure R4. a Photography of the tape-peeling test. **b** Surface of the T-PAC after being treated by the multiple tap cutters. **c** Surface of the T-PAC after tape-peeling test.

3. For the proposed composite coating, the hydration or gelation mechanism, the phase assemblage, the microstructure and the adhesion mechanism between different layers are all critical for the analysis of the anti-corrosion performance. The lack of this information limited the depth of mechanism demonstration in this work.

Response: In response to the reviewer's comment for deeper mechanistic insight, we have added a new section (Section 2.6, Pages 21-23, Lines 613-702) to establish a clear materials/structure-property relationship. The revisions elucidate that the coating's superior performance originates from three integrated mechanisms: **i)** A carbonation-activated gelation of γ-C_2S forms the primary gel matrix, with $BaSO_4$ NPs catalyzing the aragonite-to-calcite transition. This enhances solar reflectance to ~94.35%, reducing surface temperature by up to 28 °C and significantly suppressing Cl^- transport and corrosion kinetics. **ii)** A designed hierarchical microstructure, featuring a densified base from carbonation and a bimodal-pore SiO_2 overlayer, simultaneously improves barrier properties and enables superhydrophobicity for radiative cooling, delaying corrosion initiation. **iii)** Robust multi-scale adhesion is achieved through chemical bonding at the substrate interface and mechanical interlocking at the upper interface, ensuring long-term protection.

Detailed revisions are summarized as follows:

1) Gelation and phase assemblage mechanism:

The primary binder of carbonated C_2S-derived coatings form through a carbonation-activated gelation process of γ-C_2S rather than conventional hydraulic hydration. The reaction proceeds as: $\gamma\text{-}C_2S + 2CO_2 + 2H_2O \rightarrow CaCO_3$ (Calcite/Aragonite) + $SiO_2\cdot nH_2O$ (Calcium-modified silicate gel)[1], which is supported by multi-modal characterization. Specifically, XRD analysis (Fig. 2j) shows the consumption of γ-C_2S and the formation of carbonate phases, while TGA (Fig. 2l) quantifies the associated mass loss from carbonate decomposition. ATR-FTIR (Fig. 2k) further provides direct evidence of the resulting silicate gel (a C–S–H-like phase) through its characteristic Si–O and O–H vibrational signals. Additionally, the coating's phase evolution is notably catalyzed by incorporated $BaSO_4$ NPs, which lower the energy barrier for calcite nucleation ($\Delta G^*_{\_het} \approx 8.96 \times 10^{-21}$ J *vs.* $\Delta G^*_{\_hom} \approx 7.03 \times 10^{-19}$ J, Supplementary note 4), thereby promoting the transition from metastable aragonite to stable calcite and enhancing the matrix's crystallinity and mechanical robustness. Owing to calcite's wider bandgap (~1.0 eV higher than aragonite, calculated as 4.23 eV)[2], the coating exhibits

superior solar reflectance. This reflectance is further amplified by the additional scattering interfaces introduced by $BaSO_4$ NPs, leading to a remarkable increase from 76.14% to ~94.35% in both CB-C_2S and T-PAC systems (Figure 4a). Such high solar reflectance significantly reduces thermal energy absorption, lowering the surface temperature by up to 28 °C compared to ordinary Portland cement. Consequently, the coating suppresses Cl^- transport by 66.8% (Supplementary Note 6) and reduces Arrhenius-driven corrosion kinetics by 71.6% (Supplementary Note 5).

2) Microstructure and pore structure evolution:

The final T-PAC coating exhibits a unique hierarchical structure (Figs. 2c-f), revealing a dense, carbonated C_2S/$BaSO_4$ base layer seamlessly fused to the concrete substrate, overlaid by a densely packed hydrophobic SiO_2 nanosphere layer that introduces micro-nano voids. MIP data (Fig. 2i) quantitatively corroborate that this structure arises from a sequence of pore-structure modifications: carbonation of C_2S substantially reduces the nanoporosity, while additional incorporation of $BaSO_4$ and spray-coating of SiO_2 establishes a favorable bimodal pore distribution. This tailored pore structure underpins the coating's dual functionality: diminished nanoporosity in matrix enhances the barrier properties, whereas the hierarchical porosity and surface roughness are essential for achieving superhydrophobicity and augmenting light scattering for effective radiative cooling. Furthermore, the densified morphology and lower overall porosity, characteristic of both CB-C_2S and T-PAC, improve corrosion resistance by substantially limiting capillary absorption of seawater. This integrated structural advantage results in a collective delay of corrosion initiation by approximately 2.5 times (CB-C_2S vs. C-C_2S, Fig. 5h).

3) Adhesion mechanism between layers:

The coating system in our research exhibits robust adhesion through distinct mechanisms at each interface. At the gel matrix-substrate interface, the carbonation-derived silicate gel shares an identical composition with the main binding phase (calcium silicate hydrate) in concrete substrate, which facilitates direct chemical bonding[3] rather than mere physical attachment. This chemical affinity yields an exceptional interfacial strength of 1.61 MPa (Fig. 2g), significantly exceeding the 0.4–0.8 MPa range typical of conventional cementitious coatings[4]. At the SiO_2-gel matrix interface, the adhesion of superhydrophobic SiO_2 overlayer to the underlying gel matrix relies on mechanical interlocking and interparticle forces, including hydrogen bonding and van der Waals interactions. The carbonated gel surface provides sufficient nanoscale roughness, together with the use of ethanol as a spraying medium—which promotes capillary penetration into the gel pores—ensuring that the SiO_2 nanoparticles become firmly anchored upon solvent evaporation. Collectively, this robust, multi-mechanism adhesion effectively prevents delamination (Figure R4), thereby providing durable anti-corrosion performance under coastal environments.

In conclusion, these detailed mechanistic elucidations directly link the intrinsic material properties—chemically bonded gel matrix, $BaSO_4$-enhanced crystallinity, hierarchical pore structure, and robust multi-scale adhesion—to the coating's demonstrated anti-corrosion performance. We thank the reviewer for prompting this significant improvement in the depth of our work. Please see the detailed revision in the revised manuscript (Pages 21-23, Lines 613-702).

4. The experimental program for the anti-corrosion performance should present the sample illustration and the setup for the electrochemical tests. The information of concrete type and cover thickness, sealing of the steel end, coating thickness et al. are all important for the analysis of the test results. According to Figure S17, the

surface conditions of the steel bars are quite different, and it will influence the corrosion initiation induced by chloride ion. The corrosion morphology and the much lower initial Z modulus of OPC, C_2S and C-C_2S samples are strange, and detailed analysis is needed. There seems no passivation in concrete.

Response: The reviewer's constructive suggestions regarding the experimental details and electrochemical results have been rigorously addressed through comprehensive revisions to the manuscript: First, all critical parameters (e.g., cement grade, w/c ratio, coating thickness) and a schematic of the electrochemical setup (Supplementary Figure S28) have been included in the revised Materials and Methods. Second, a detailed analysis linking the distinct post-test steel surface morphologies to coating performance has been added. Third, the analysis of the low initial low-frequency impedance ($|Z|_{0.01}$) for control samples has been incorporated (Section 2.5).

Detailed responses are shown below:

For the experimental details, we have systematically expanded the Materials and Methods section (Page 27, Line 829; Page28, Lines 849-850, 852-854, 856-858; Page 30, Lines 923-925) to include a complete set of experimental parameters, such as the use of 42.5-grade ordinary Portland cement (with a w/c = 0.4), a 10 mm cover thickness, two-end sealed steel rebars, controlled coating thickness of 50 ~ 300 μm, and three-electrode system with saturated calomel reference. Additionally, a new Supplementary Figure S28 has been incorporated to schematically illustrate the configuration of the three-electrode electrochemical cell (Figure R5).

[Figure Redacted]

Figure R5. Schematic diagram showing sample illustration and the setup for the electrochemical tests in this study.

The distinct surface conditions of the steel rebars observed in Figure S17 are direct results of the differing levels of protection offered by the coatings. Prior to sample preparation, all steel rebars were meticulously polished to a smooth surface to eliminate potential influence of initial defects or surface roughness on the corrosion results. Therefore, the distinct morphologies observed post-testing are unequivocally attributable to the performance of the respective coatings during the exposure period. Specifically, the severely degraded, rough surfaces with filamentous rust signs on the OPC, C_2S, C-C_2S, and even the CB-C_2S samples indicate a state of active and non-uniform corrosion. The roughness is a consequence of the widespread breakdown of

the passive film and the formation of porous, non-protective corrosion products (e.g., FeOOH, Fe_2O_3, Fe_3O_4) as chloride ions penetrated the inadequate protective layers. The presence of both corroded and seemingly intact areas on the same rebar, particularly in OPC, C_2S and C-C_2S, suggests the formation of localized macro-cells, where anodic sites (corroding) and cathodic sites (passive) coexist, accelerating the deterioration process. In stark contrast, the smooth and pristine surface of the T-PAC sample is direct visual proof of its exceptional barrier property. The coating successfully prevented chloride ions and moisture from reaching the steel surface, thereby preserving the initial passivity and preventing any visual corrosion initiation. The superior surface condition of the T-PAC sample validates the synergistic protective effect of its integrated radiative cooling and superhydrophobic layers. Moreover, it is also noteworthy that the smooth and pristine surface of the steel bar in T-PAC sample enabled by the synergistic protective effect leads to a metallic luster, which may give a visual impression of an unpassivated surface. Its pristine surface condition, without noticeable corrosion site, requires increased illumination to resolve surface details during photography. This heightened light intensity resulted in the pronounced metallic reflection, in contrast to the matte appearance of the uncorroded areas on the other samples due to micro-scale surface heterogeneity. However, it is crucial to emphasize that the fundamental conclusion regarding the superior corrosion protection of the T-PAC coating is drawn from the stark contrast in physical and chemical surface morphology rather than from optical appearance alone. We have incorporated this detailed analysis into the revised manuscript (Pages 19-20, Lines 589-600) to more thoroughly link the observed surface conditions to the corrosion protection mechanisms.

Regarding corrosion morphology, the uniform and irregular corrosion across the OPC sample is characteristic of a complete lack of protection. The hydrophilic and porous concrete cover offers no significant barrier, allowing chloride ions to reach the entire steel surface homogeneously, leading to generalized corrosion in high-Cl^- environments. The non-uniform, patchy corrosion on the C_2S sample suggests the formation of macro-cell corrosion, because the C_2S layer provides inconsistent coverage and limited barrier effect. This creates distinct anodic (heavily corroded areas) and cathatic (less corroded areas) regions on the same rebar, where the anodic sites suffer accelerated localized attack. In C-C_2S rebar, the localized, thick rust patch at the upper section is a classic sign of localized pitting corrosion as carbonation densifies the matrix but does not impart hydrophobicity. The occurrence of corrosion in the upper area, potentially due to minor imperfections or being the primary path of chloride ingress from the electrolyte, became the preferential site for pit initiation. Thus, once a pit starts, the large cathode (the entire passive surface) to small anode (the pit) area ratio drives intense, localized corrosion, resulting in the observed thick, bulky rust products. In striking contrast, the CB-C_2S sample, with its radiative cooling layer, shows only small, isolated patches, indicating a significantly delayed and restricted corrosion initiation. The superior performance of the T-PAC sample, which remained virtually free of rust, unequivocally demonstrates the synergistic effect of combining the radiative cooling layer (CB-C_2S) with the superhydrophobic top layer, which effectively blocks both thermal and chemical drivers of corrosion.

The low initial $|Z|_{0.01}$ values in OPC/C_2S/C-C_2S samples result from: (1) Rapid chloride penetration through hydrophilic surfaces. (2) Limited passivation in high-Cl^- environments (3.5 wt.% NaCl). We have incorporated this analysis in Section 2.5 (Pages 18-19, Lines 555-559).

5. A temperature-wetting mediated passive anti-corrosion coating is proposed in this work. One of the highlights in this work may lie in the suppression efficiency of passive cooling performance of the coating on the corrosion behavior of steel in concrete, and to what degree the temperature regulation affects the corrosion

behavior. To quantify the effect of passive cooling performance on the transport of chloride ion, the corrosion initiation and the corrosion rate might be critical issues to be addressed in this work. The analysis of corrosion behavior is superficial and lacks synergy and quantitative contribution analysis. The frame of the work should be reorganized to emphasize this highlight.

Response: In response to the reviewer's insightful comment on quantifying the individual contribution of passive cooling to corrosion suppression, we have added quantitative analysis demonstrating that passive cooling alone reduces the corrosion rate by 71.6% and chloride diffusion by 66.8%. These two effects synergistically extend the theoretical corrosion initiation time by approximately 15.1 times. Further, we experimentally demonstrated that the integration between cooling and superhydrophobicity is synergistic (Synergy Index > 1), not merely additive, which enables a multi-stage defense: the hydrophobic layer blocks initial ingress, while cooling retards subsequent diffusion and reaction kinetics. This mechanistic elaboration is now centralized in the revised manuscript.

Below are the detailed responses:

1. Quantifying the effect on corrosion rate

1.1 Calculation basis and experimental data

The Arrhenius equation describes the relationship between the reaction rate constant (k) and temperature (T):

$$k = A\exp\left(-\frac{E_a}{RT}\right) \tag{1}$$

where k is the reaction rate constant, A is the pre-exponential factor (related to the collision frequency), E_a is the activation energy for the reaction (we adapted a well-established value of ~40 kJ/mol for cement paste[5]), R is the universal gas constant (8.314 $J\cdot mol^{-1}\cdot K^{-1}$), T is the absolute temperature in Kelvin (K).

For two different temperatures T_1 (uncoated) and T_2 (coated), the relationship between temperature and corrosion rate follows the Arrhenius equation:

$$\frac{i_{corr}(T_2)}{i_{corr}(T_1)} = \frac{i_0\cdot\exp\left(-\frac{E_a}{RT_2}\right)}{i_0\cdot\exp\left(-\frac{E_a}{RT_1}\right)} = \exp\left[-\frac{E_a}{R}\left(\frac{1}{T_2}-\frac{1}{T_1}\right)\right] \tag{2}$$

Our experimental temperature measurements during the peak solar period (11:00-13:00) showed:

- Average ambient temperature: $T_{ambient}$ = 26.56 °C =299.8 K
- Average coated surface temperature: T_{coated} = 24.35 °C =297.5 K (2.3 °C below ambient)
- Average uncoated concrete temperature: $T_{uncoated}$ = 49.55 °C =322.7 K (25.2 °C above coated surface)

1.2 Calculation Procedure

Calculate the reciprocal temperature difference:

$$\frac{1}{T_{coated}} = \frac{1}{297.50} = 0.003361\ K^{-1}$$

$$\frac{1}{T_{\text{uncoated}}} = \frac{1}{322.70} = 0.003099\ \text{K}^{-1}$$

$$\Delta(\frac{1}{T}) = \frac{1}{T_{\text{coated}}} - \frac{1}{T_{\text{uncoated}}} = 0.003361 - 0.003099 = 0.000262\ \text{K}^{-1}$$

Calculate the exponent term:

$$-\frac{E_a}{R} = -\frac{40000}{8.314} = -4811.6$$

$$\text{Exponent} = -\frac{E_a}{R} \times \Delta(\frac{1}{T}) = -4811.6 \times 0.000262 = -1.26$$

Calculate the corrosion current density ratio:

$$\frac{i_{\text{corr}}(T_{\text{coated}})}{i_{\text{corr}}(T_{\text{uncoated}})} = \exp\,(-1.26) = 0.284$$

This means the corrosion current density for the coated sample is only 28.4% of that for the uncoated concrete. Therefore, the passive cooling effect alone provides a 71.6% reduction in corrosion rate, a result that aligns closely with our measured value of 74.3% (calculated as [$i_{\text{corr\_OPC}}$ - $i_{\text{corr\_CB-C_2S}}$] / $i_{\text{corr\_OPC}}$). The slight discrepancy of 2.7% is primarily due to an additional physical barrier provided by the CB-C_2S layer and, consequently, resulting in an underestimation in the theoretical calculations of the corrosion rate–temperature relationship. This calculation demonstrates that temperature regulation is a pivotal mechanism in the coating's overall anti-corrosion strategy, and we have added this quantitative analysis to the revised manuscript (Page 23, Lines 705-713).

2. Quantifying the effect on chloride ion transport

To quantitatively address the multifaceted impact of passive cooling on chloride transport and corrosion processes, we then performed a comprehensive analysis based on established physical models and our experimental data. The key findings are summarized as follows:

2.1 Theoretical derivation of the temperature-dependent diffusion coefficient:

The temperature dependence of the chloride diffusion coefficient follows the Arrhenius-type relationship[6] derived from the transition state theory for ionic transport in porous materials. The fundamental form is:

$$D_{Cl}(T) = D_0 \cdot \exp\,(-\frac{E_{a,diff}}{RT}) \tag{3}$$

where D_0 is the pre-exponential factor (m^2/s), $E_{a,diff}$ is the apparent activation energy for chloride diffusion (J/mol), typically ranging from 30–45 kJ/mol in cementitious systems[6].

2.2 Derivation of the relative diffusion ratio:

For two different temperatures T_1 (uncoated) and T_2 (coated), the ratio of diffusion coefficients is derived as follows:

$$\frac{D_{Cl}(T_2)}{D_{Cl}(T_1)} = \frac{D_0 \cdot \exp\,(-\frac{E_{a,diff}}{RT_2})}{D_0 \cdot \exp\,(-\frac{E_{a,diff}}{RT_1})} = \exp\,[-\frac{E_{a,diff}}{R}(\frac{1}{T_2} - \frac{1}{T_1})] \tag{4}$$

Using our experimental data:

$T_1 = 322.70\ \mathrm{K}$ (49.55°C, uncoated)

$T_2 = 297.50\ \mathrm{K}$ (24.35°C, coated)

$E_{a,diff} = 35\ \mathrm{kJ/mol} = 35{,}000\ \mathrm{J/mol}$ (a conservative situation)

$$\frac{D_{Cl}(T_2)}{D_{Cl}(T_1)} = \exp\left[-\frac{35000}{8.314}\left(\frac{1}{297.50} - \frac{1}{322.70}\right)\right] = \exp(-1.103) \approx 0.332$$

This indicates a 66.8% reduction in the chloride diffusion coefficient due to the temperature reduction, which is quite close to the experimental results (69.05%) of a previous study[6], conducted at similar temperature conditions. This quantitative analysis has been included in the revised manuscript (Page 23, Lines 713-719).

3. Quantifying the effect on corrosion initiation time

3.1 Derivation from Fick's second law

The time for chloride ions to reach the critical concentration at the steel surface can be estimated by the error function solution to Fick's second law under non-steady-state conditions for a semi-infinite medium[7] :

$$C(x,t) = C_s \cdot \left[1 - \mathrm{erf}\left(\frac{x}{2\sqrt{D \cdot t}}\right)\right] \tag{5}$$

where $C(x,t)$ is the chloride concentration at depth x and time t, C_s is the surface chloride concentration, D is the chloride diffusion coefficient.

The corrosion initiation time t_{init} occurs when $C(x,t)$ at the steel depth $x = d$ equals the critical chloride threshold C_{cr}:

$$\frac{C_{cr}}{C_s} = 1 - \mathrm{erf}\left(\frac{d}{2\sqrt{D \cdot t_{init}}}\right) \tag{6}$$

3.2 Derivation of the time ratio

For a fixed depth d and concentration ratio C_{cr}/C_s, the argument of the error function must remain constant. Therefore:

$$\frac{d}{2\sqrt{D_1 \cdot t_{init,1}}} = \frac{d}{2\sqrt{D_2 \cdot t_{init,2}}} \tag{7}$$

The ratio of initiation times for two different conditions is given by:

$$\frac{t_{init,coated}}{t_{init,uncoated}} = \frac{D_{uncoated}}{D_{coated}} \cdot \left(\frac{C_{cr,coated}}{C_{cr,uncoated}}\right)^2 \tag{8}$$

This equation reveals that the extension of service life is proportional to the reduction in the diffusion coefficient and the square of the increase in the critical chloride threshold. Substituting the calculated value of relative diffusion ratio ($D_{Cl,\,T_1} / D_{Cl,\,T_2} = 3.012$), the ratio of initiation times for two different temperatures can be expressed as:

$$\frac{t_{init,coated}}{t_{init,uncoated}} = 3.012 \cdot \left(\frac{C_{cr,coated}}{C_{cr,uncoated}}\right)^2 \tag{9}$$

3.3 Quantifying the increase in critical chloride threshold ($\frac{C_{cr,coated}}{C_{cr,uncoated}}$)

The critical chloride threshold is also highly temperature dependent. An empirical relationship states that the threshold decreases by approximately ~36% for every 10 ℃ increase in temperature[8]. The relationship can be expressed as:

$$\frac{C_{cr,coated}}{C_{cr,uncoated}} = (1.36)^{\Delta T/10} \quad (10)$$

where ΔT is the temperature drop in ℃. Based on our temperature results, we have:

$$\frac{C_{cr,coated}}{C_{cr,uncoated}} = (1.36)^{\frac{25.2}{10}} = (1.36)^{2.52} \approx 2.17$$

therefore, the cooling effect increases the critical chloride threshold by a factor of 2.17.

3.4 Final calculation of initiation time extension

We now combine the two effects in the theoretical model:

$$\frac{t_{init,coated}}{t_{init,uncoated}} = \frac{D_{uncoated}}{D_{coated}} \cdot \left(\frac{C_{cr,coated}}{C_{cr,uncoated}}\right)^2 = 3.012 \times (2.17)^2 = 3.21 \times 4.71 \approx 15.1$$

This indicates that the initiation time is calculated to be extended by approximately 15.1-fold solely due to the passive cooling effect. These derivations and calculations have been incorporated into the revised manuscript (Pages 23-24, Lines 721-726) to provide rigorous theoretical foundation for our experimental findings.

4. Synergistic analysis

We have added a new paragraph (Page 24, Lines 727-746) to provide a clear decomposition of the individual and synergistic contributions, using our experiments data: OPC (130.868 μA/cm^2), C_2S (107.514 μA/cm^2), C-C_2S (60.447 μA/cm^2), CB-C_2S (33.644 μA/cm^2), and T-PAC (1.453 μA/cm^2).

4.1 Quantitative analysis of individual contributions and synergistic enhancement

To accurately isolate the effects of each functionality, we employed a step-by-step analytical framework, with each step building upon the previous one to quantify the added value of each component.

**Sample**	**Functionality**	**i_{corr} (μA/cm^2)**	**Protection Efficiency***	**Purpose in Analysis**
**OPC**	None (Baseline)	130.868	0%	Reference for no protection
**C-C_2S**	Dense Barrier (Carbonation)	60.447	53.8%	Base improvement from densification
**CB-C_2S**	Dense Barrier + Radiative Cooling	33.644	74.3%	Quantify added value of radiative cooling
**T-PAC**	Full System (All	1.453	98.9%	Quantify total synergy

Sample	Functionality	i_{corr} (μA/cm^2)	Protection Efficiency*	Purpose in Analysis
	components)			

*Protection Efficiency (PE) = [1 - ($i_{corr, sample}$ / $i_{corr, OPC}$)] × 100%

4.1.1 Establishing the baseline improvement (C-C_2S *vs*. OPC):

First, the carbonation (C-C_2S) itself provides a baseline improvement, reducing i_{corr} by approximately 54% compared to OPC. This establishes the "base" upon which the innovative functionalities are added.

4.1.2 Quantifying the individual contribution of radiative cooling (CB-C_2S *vs*. C-C_2S):

The effect of adding radiative cooling to the dense barrier is calculated by comparing CB-C_2S to C-C_2S:

- i_{corr} reduction: 60.447 - 33.644 = 26.803 μA/cm^2
- Contribution of radiative cooling: 26.803 / 60.447 ≈ 44.3% reduction relative to the dense barrier baseline.

4.1.3 Quantifying the individual contribution of superhydrophobic barrier (T-PAC *vs*. CB-C_2S):

We assess the effect of adding the superhydrophobic barrier to the "Barrier + Cooling" system (CB-C_2S) by comparing it to the full T-PAC system.

- i_{corr} reduction: 33.644 - 1.453 = 32.191 μA/cm^2
- Net Effect of Superhydrophobicity: 32.191 / 33.644 ≈ 95.7% reduction relative to the "Barrier + Cooling" baseline.

4.1.4 Quantifying the overall synergy

If the effects were purely multiplicative, the theoretical i_{corr} of T-PAC would be:

i_{corr}, theoretical = 130.868 × (1 - 53.8%) × (1 - 44.3%) × (1 - 95.7%) ≈ 2.94 μA/cm^2

The actual measured i_{corr} of the full T-PAC system is 1.453 μA/cm^2, which is 50.6% lower than this theoretical multiplicative value, demonstrating that the combination achieves substantially better performance than predicted by simple additive models.

We then quantify the synergy between radiative cooling and superhydrophobic barrier using a Synergy Index (SI, a probability model for independent events[9]) calculated as:

$$SI = (PE\_{combined}) / (PE\_{cooling} + PE\_{barrier} - PE\_{cooling} \times PE\_{barrier})$$

where $PE\_{barrier}$ represents the estimated protection efficiency of the superhydrophobic barrier alone. Based on the dramatic performance improvement observed when individually adding the superhydrophobic barrier or the radiative cooling functionality to the system, we estimate the synergy index to be:

$$SI = 98.9\% / (44.3\% + 95.7\% - 44.3\% \times 95.7\%) \approx 1.013$$

An SI > 1 quantitatively demonstrates that the two functions are not merely independent but interact

synergistically, producing a combined effect that surpasses the theoretical prediction. Our calculated SI of 1.013 provides concrete evidence for this positive synergy, which we attribute to the mutual protection mechanisms described below.

4.2 In-depth analysis of the synergistic mechanism (Pages 24-25, Lines 747-774)

The synergy is not merely additive but mutually protective and enhancing, operating through the following mechanisms (Figure R1):

4.2.1 Cooling-augmented barrier durability:

The radiative cooling layer does more than just slow down kinetics. By maintaining a sub-ambient surface temperature, it reduces the thermal stress on the hydrophobic SiO_2 overlayer. This mitigates thermos-degradation (e.g., oxidation, cracking), which are common failure modes for nano-composite coatings. More importantly, the sub-ambient cooling effect helps to stabilize the three-phase contact line, which maintains air-trapping Cassie-Baxter state and prevents an irreversible transition to the wetted Wenzel state. This is crucial for suppressing surface condensation (Figure R2), particularly during high-humidity nights, and facilitating the rapid recovery of water repellence essential for long-term corrosion protection.

4.2.2 Barrier-protected cooling efficiency:

The superhydrophobic layer plays an active role in this synergy. It acts as an impermeable frontline barrier, preventing the pore blockage by seawater and the adhesion of contaminants. This protection is crucial for maintaining the underlying radiative cooling layer's high solar reflectance (94.6%) and thermal emissivity over the long term. Our tests corroborate this mechanism, showing that even after being treated to viscous mud slurry, the T-PAC recovered its clean and white surface (Figure R3a) with its thermal regulation performance remains unchanged upon fouling shedding (Figure R3c). This capability is of great significance for durable corrosion protection, as a fouled (Figure R3b) or wet surface (Figure 4c) would suffer from plummeting reflectance, which would severely compromise cooling performance (Figure R3d).

4.2.3 Multi-stage corrosion suppression (Page 25, Lines 775-790):

The synergy provides comprehensive defense through complementary mechanisms (Figure R6):

- Stage I (Initiation): The superhydrophobic layer is the primary defense, physically blocking >90% of chloride ingress (Fig. 3h).
- Stage II (diffusion): The cooling function provides secondary defense by reducing the chloride diffusion coefficient by ~66.8%, dramatically delaying the time for any residual chlorides to reach the steel surface.
- Stage III (Propagation): Once chlorides breach the barrier, the cooling layer becomes the primary defense, reducing the corrosion reaction rate by ~74.3% via Arrhenius kinetics suppression (Fig. 5h).

Thus, the superhydrophobic layer acts as a primary shield, drastically reducing initial chloride ingress. The radiative cooling function then serves as a powerful secondary defense, significantly slowing the diffusion and electrochemical reaction kinetics of any residual chlorides, their combination successfully mitigates the primary failure modes of each individual function, leading to a system whose overall performance is greater than the sum of its parts, achieving near-complete corrosion suppression.

[Figure Redacted]

Figure R6. Schematic diagram showing the multi-stage corrosion protection mechanism of T-PAC.

These calculations quantitatively demonstrate that the combination of superhydrophobic barrier and radiative cooling in the T-PAC coating creates a synergistic, multi-stage defense system, which delivers a deep, quantitative, and mechanistic understanding of how the T-PAC coating achieves its exceptional performance. We have incorporated this rigorous quantitative and mechanistic discussion into the revised manuscript (Pages 24-25; Lines 747-790) to better highlight this key finding as suggested by the reviewer.

5. Reorganizing the frame of our work:

The reviewer's comment on reorganizing the work's frame is also well-taken. We have undertaken a major reorganization of the manuscript's framework to place the highlight of this work at the forefront and ensure it permeates the entire narrative. The key revisions are summarized below:

5.1 Revised introduction (Page 2, Lines 49-50):

The introduction now opens with the critical challenge of thermo-wetting coupling in marine corrosion. We immediately position temperature-mediated suppression as a significant, yet underexplored, pathway. Our coating is then introduced as a material solution designed specifically to exploit this novel pathway through synergistic integration with superhydrophobicity.

5.2 Restructured results and discussion:

We have added a new Section (Section 2.6, Page 21) to establish a logical, cause-effect narrative that centralizes the corrosion suppression mechanism. The discussion section is now tightly focused on interpreting the EIS results through the lens of "materials-performance correlation, multi-stage synergistic suppression". Detailed revisions are summarized as follows:

- Correlation between intrinsic material properties and anticorrosion performance as the entry

point: The analysis begins with intrinsic materials properties analysis, directly linking them to the enhanced performance of T-PAC compared to the control samples.

- Quantifying the cooling contribution: Using the Arrhenius model and measured temperature differentials, we quantify the specific suppression effect of radiative cooling on corrosion kinetics (74.3% reduction in corrosion rate), followed by showing how cooling retards chloride diffusion, thereby extending the corrosion initiation time
- Mechanistic synergy analysis: The spectral, morphological, and phase characterization are integrated to provide a material-based explanation of how the cooling and hydrophobic layers interact to enhance durability and barrier performance, making the narrative more compelling.

The newly introduced Section 2.6 transforms the discussion into a coherent narrative that progresses from performance observation to mechanistic quantification, culminating in a unified interpretation under the "multi-stage synergistic suppression" framework.

5.3 Strengthened conclusion (Page 27, Lines 808, 819-822):

The conclusion has been strengthened to explicitly reflect how the temperature-wetting mediation paradigm established in this work offers a new design framework for next-generation anti-corrosion coatings, moving beyond conventional single-mechanism strategies.

In essence, we have transformed the manuscript into a compelling demonstration of a novel anti-corrosion principle. We envision that this refocused framework will improve the central contribution highlighted by the reviewer and significantly improve the clarity and impact of this work.

6. The anti-wetting and anti-corrosion experiments are mainly based on short-term tests (no longer than 30 days). The long-term durability of $C_2S/BaSO_4$ composites coating steel needs to be verified. The physiochemical properties are also suggested to be studied, including the UV aging, abrasion resistance, weathering resistance, chemical stability and even the biofouling. If available, even preliminary data from a field exposure test (e.g., in a splash zone) would greatly enhance the impact of the manuscript.

Response: Following the reviewer's advice, we have conducted comprehensive durability assessments and studied the physiochemical properties of T-PAC. Our multi-stress accelerated corrosion protocol (combining salt spray, solar irradiation, and dry-wet cycles) is exceptionally severe, with control samples failing rapidly within several cycles. The T-PAC coating's excellent performance over 30 such cycles is conservatively estimated to be equivalent to ~79 years of service in a marine atmosphere. Furthermore, separate long-term tests confirm its persistent water repellency, UV resistance (>95% property retention after 1000 h), abrasion tolerance, and anti-biofouling capability, collectively demonstrating its robustness for practical application.

Detailed responses to durability concerns are summarized as follows:

6.1 Response to the comment on short-term tests of anti-corrosion performance

In our study, we employed a specially designed accelerated aging protocol that subjects the coating to extreme cyclic conditions, significantly harsher than standard laboratory tests. The exceptional performance of our T-PAC coating over 30 cycles in this rigorous regime is a robust indicator of its long-term durability,

which is actually equivalent to much longer exposure in real-world, less aggressive conditions.

6.1.1 The accelerated and harsh nature of our testing protocol

Regarding the “short-term tests”, we note that the 30 cycles of our test, while equivalent to only 30 days in absolute time, subject the coating to an intensity of coupled thermo-chemo-hygroscopic stress that would be accumulated over a much longer period in a natural marine splash zone environment. In particular, our 30-cycle corrosion test was intentionally designed to be an accelerated and severe evaluation, not a mild, short-term exposure. Accordingly, the duration alone does not reflect the severity of our test. The key to this severity lies in the synergistic application of multiple stressors (3-hour salt spray + 3-hour solar irradiation + 18-hour ambient exposure) within each 24-hour cycle:

- Chemical stressor (Salt spray): This is a standard accelerated corrosion test method that simulates years of marine atmospheric exposure in a short time by creating continuous, high-concentration saline fog.
- Thermal and radiation stressor: A 3-hour high-intensity solar irradiation phase (1000 W/m^2) not only rapidly drives temperature fluctuations and chloride diffusion rate[10] but also provides the energy to accelerate electrochemical reactions at the steel-concrete interface[11] , should chlorides have reached it.
- Dry-wet cycles: The introduction of intense solar heating and ambient drying creates repeated thermal and hydration stresses. This cycle is far more damaging than a continuous immersion test[12,13], as it promotes the crystallization of salts within pores and microcracks, mechanically stressing the coating and accelerating degradation. This “weathering” effect is a key factor in long-term coating failure[14].

This multi-stress protocol is far more aggressive than standard single-mechanism tests (e.g., continuous salt spray or static immersion[15]). The fact that our control samples (OPC, C_2S, C-C_2S) failed sequentially and rapidly (within 1 to 11 cycles) under these conditions underscores the severity of our test. In a milder, standard test, these controls would likely have survived for a much longer duration. Combining these findings, we can deduce that our 30-cycle test for the T-PAC coating is equivalent to a significantly longer period of standard exposure.

6.1.2 Equivalent service life estimation based on experimental data

Corrosion initiation in costal concrete structure usually takes several years or decades[16]. Our test, by inducing multiple aggressive wet-dry-thermal cycles per day, compresses the degradation timeline. Although directly correlating our 30 accelerated cycles to a specific number of years of field service requires long-term monitoring data, we can provide a conservative engineering estimate based on the experimental data of our samples and established equivalence between indoor chlorine salt dry-wet cycling tests and actual engineering exposure time. According to a prediction model of equivalent exposure time in indoor dry-wet cycling tests for concrete in costal zones, 1 chlorine salt dry-wet cycle in an indoor simulated harbor environment (average temperature of 12.6°C and average relative humidity of 56.7 %) is equivalent to 2.63 years of chloride salt exposure erosion in an actual project, which is verified through experiments data from over 20 coastal structures across 6 countries and outdoor observation time from 0.5 to 44.4 years[17] . Compared to the single-mechanism dry-wet cycling test, our multi-stress accelerated protocol (solar radiation + dry/wet cycles) is much harsher. Accordingly, based on this correlation, our 30 cycles can be estimated to represent at least 30 days of standard salt dry-wet cycling tests, which is indicative of ~79 years[17] of service life in a typical marine atmosphere, thus adequately addressing the long-term durability concern. Please note that the correlation is

not perfect, and the linkage between 30 accelerated dry-wet cycles and 79 years of coastal exposure is not a universal rule. But this equivalence allows for the quick evaluation of our protective coatings to see how they would perform in a natural marine environment without waiting years for filed exposure test data. We have added these discussions in the revised manuscript (Page 19, Lines 567-573).

6.2 Response to the comment on long-term durability of anti-wetting performance

The reviewer's concern about the "short-term" nature of the tests can be further addressed by our separate long-term water repellency assessment (Figure 3g). In this test, we evaluated the permeability of coatings applied to filter paper by monitoring the transmission of a 3.5 wt% NaCl solution over time. The results were striking: All control samples reached their saturation plateau within 2,000 minutes (≈1.4 days). The CB-C_2S sample (with radiative cooling but without the full superhydrophobic barrier) took approximately 10,000 minutes (≈6.9 days) to reach equilibrium. By contrast, the T-PAC coating exhibited an exceptionally low transmission rate, with only a negligible amount of NaCl solution passing through even after 10,000 minutes, which may occur through limited surface imperfections or imperfectly sealed microvoids within the coating. Moreover, after 30 days of continuous immersion in seawater, the T-PAC exhibited negligible deterioration in solar reflectance (Figure R10d), further illustrating its durable anti-wetting performance. More importantly, considering that T-PAC is deployed in the splash zone—where it experiences intermittent seawater exposure rather than full immersion—these results should correspond to a significantly longer service life under practical conditions. This experiment demonstrates that the impermeability of the T-PAC coating persists effectively over a duration several times longer than that required for conventional coatings to fail completely. Although the water repellency of the T-PAC may degrade over time, it can be restored by simply recoating hydrophobic SiO_2 NPs (Figure R8b), eliminating the need for full coating replacement as is typically required with traditional films. This offers a direct and compelling demonstration of its long-term barrier potential.

Thus, we have designed a corrosion test that is inherently accelerated and severe. The sequential failure of all control samples confirms the protocol's effectiveness. The outstanding performance of the T-PAC coating—showing negligible corrosion current over 30 aggressive cycles while simultaneously demonstrating superior, long-lasting water repellency in a separate weeks-long test—provides compelling and multi-faceted evidence for its long-term durability. We therefore envision that the coating possesses the robustness required for extended service life in marine environments.

6.3 Response to the comment on the T-PAC's physiochemical properties

We also conducted additional durability tests as suggested (Pages 14-15, Lines 419-446):

Accelerated UV aging and weathering resistance test. The UV radiation resistance of the T-PAC was evaluated using an accelerated weathering tester (QUV, Q-Lab). The instrument used UVA-340 fluorescent bulbs to simulate the medium-to-short wavelength range in the UV part of sunlight. To accelerate the weathering processes, the T-PAC was exposed to UV irradiance of 0.89 W m^{-2} at 340 nm and 60 °C for 1,000 h (~42 days). The UV dosage in this test is equivalent to ~275 MJ m^{-2}, which is an international benchmark for durability tests of construction materials. As illustrated in Figure R7a, after 1,000 hours of UV irradiation, the T-PAC coating retained over 95% of both its solar reflectance and water contact angle, demonstrating its exceptional resistance to UV aging. Additionally, weathering resistance tests were conducted to evaluate the comprehensive durability of T-PAC under more complex environmental conditions involving photodegradation, thermal-oxidative aging, and hydrolytic aging. This assessment was performed by directly

exposing the T-PAC on the rooftop of a building in Shenzhen, China, facing the sky under open-air conditions. Continuously monitoring revealed that, even after nearly three months of outdoor exposure—a condition under which most radiative cooling materials tend to undergo significant optical degradation—the T-PAC coating exhibited less than 4% reduction in $\bar{R}_{solar}$ (Figure R7b). Moreover, the anti-wetting properties before and after exposure showed negligible deterioration, with only a minimal decrease in the θ_c (≈ 1.1°, see inset in Figure R7b), further confirming the excellent weatherability of the T-PAC.

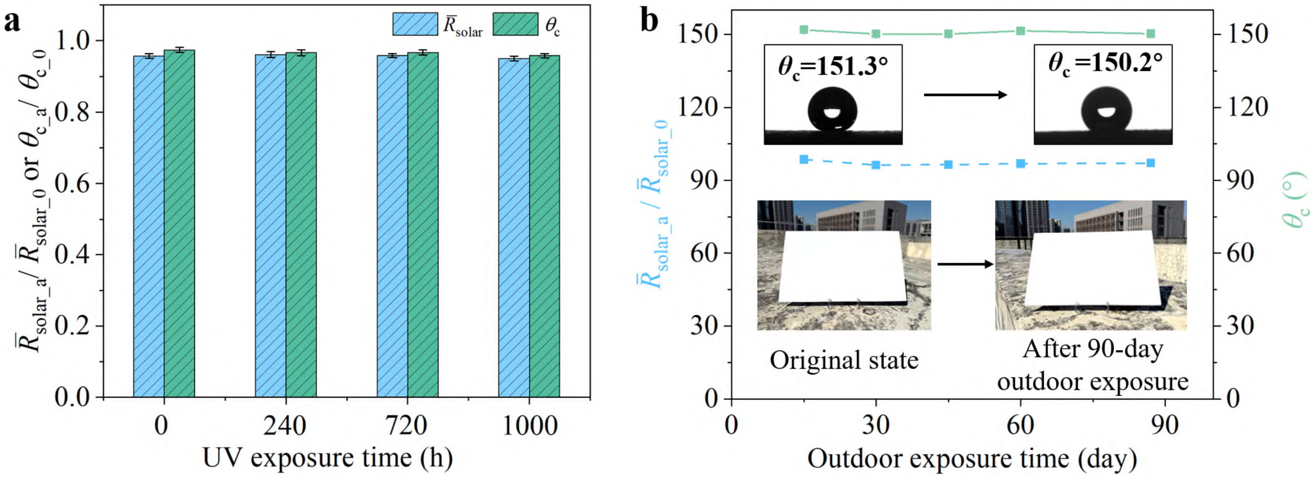

Figure R7. UV aging and weather resistance test. Solar reflectance and water contact angle of the T-PAC measured at different UV exposure times (**a**) and different outdoor exposure times (**b**).

Abrasion tolerance test. The abrasion tolerance test of the T-PAC was performed with the following steps: First, a T-PAC modified sample was gently and horizontally placed on the rough surface of a 400-mesh sandpaper. Second, a 500-gram weight was placed on the top surface of the sample to impose a normal load and ensure its intimate contact with the rough surface of the underlying sandpaper. Then, the T-PAC was pulled at a constant speed of ~0.02 m/s to induce defective sites on the T-PAC surface. The pulling distance for each test was 20 cm and the solar reflectivity as well as water contact angle were measured at pre-designed cycles. As shown in Figure R8a, when subjected to cyclic mechanical abrasions using sandpaper under 1.96 KPa, the T-PAC maintained both high solar reflectance and water repellency in the first 50 abrasion cycles and maintained a high solar reflectance even after 1,000 abrasion cycles (Figure R8b). Although the water contact angle experienced a significant decline from 150.4° to 72.2° after 50 abrasions (Inserted figure of Figure R8b), it can be restored after recoating the hydrophobic SiO_2 NPs. Please note that the T-PAC serves as a coating on the external surface of costal infrastructures, instead of a structural component, which has small chance of being abraded. Thus, from the practical application perspective, 50 abrasion cycles under a pressure of ~1.96 kPa should be sufficient to support its implementation as a coating material in coastal area. Moreover, even if the T-PAC is abraded, it can be easily repaired by repainting T-PAC on the local defective sites, rather than replacing the entire layer, as is the case with traditional film.

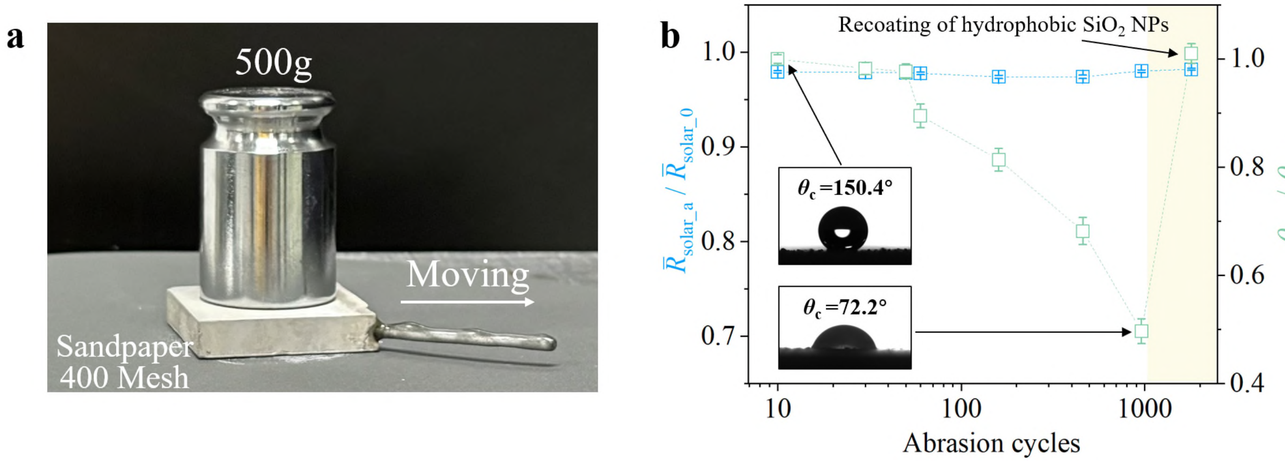

Figure R8. Abrasion tolerance test T-PAC. a. The experimental configuration for the abrasion tolerance test. **b**. The variation of solar reflectance and water repellence of T-PAC under different abrasion cycles. Inserted figures show the water droplet profile at the first 10 abrasion cycles and 1,000 abrasion cycles.

Corrosive solution resistance test. The corrosion resistance of the T-PAC was studied via the full immersion method. The samples were prepared by fully immersing the T-PAC in corrosive solutions with pH values of 0.66 and 13.03 (Figure R9a). The pH values of corrosive solutions were adjusted by 1 mol/L sodium hydroxide and 18.4 mol/L hydrochloric acid through titration. The immersion time was set to 15 min for sufficient contact in the corrosive liquid. After the immersion process, the T-PAC samples were moved into an oven and dried at 40 ℃ for 120 min. As the absorbed moisture completely evaporated, the corrosion resistance of the T-PAC was evaluated by comparing the solar reflectance and water contact angle before and after corrosive medium immersion. As shown in Figure R9b, the T-PAC samples retained high solar reflectance and remained effectively water-repellent after 15 minutes of immersion in highly aggressive corrosive solutions (at pH 0.66 and 13.03). This result directly demonstrates the exceptional robustness of the T-PAC coating.

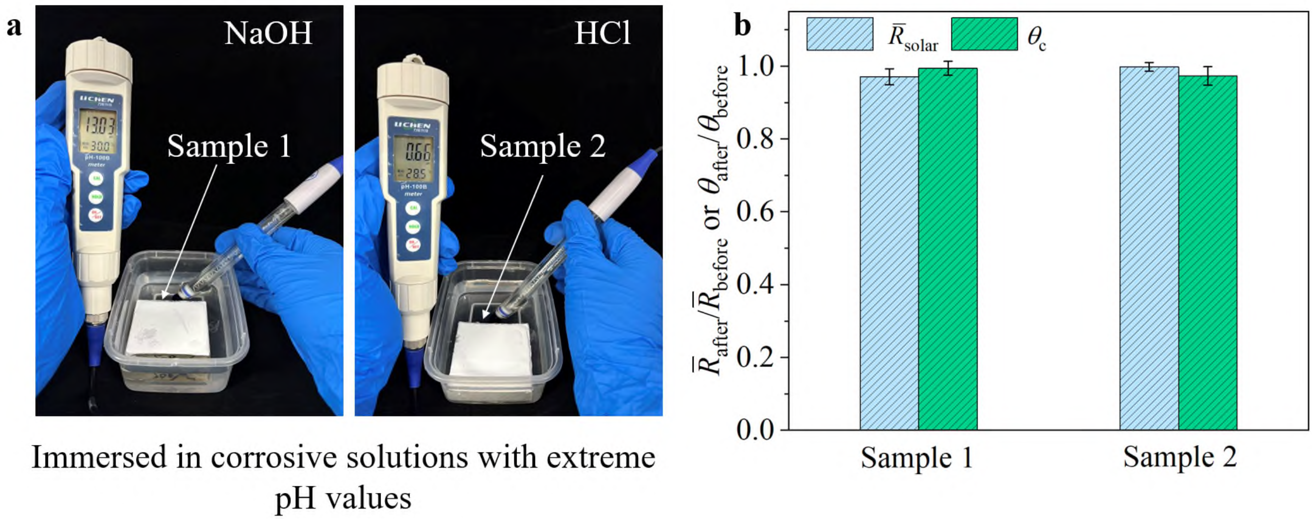

Figure R9. a. Corrosion resistance test of T-PAC in corrosive solutions (NaOH solution with a pH of 13.03 and HCl solution with a pH of 0.66). **b**. Results of solar reflectance and water repellence of T-PAC after being immersed in the corrosive solutions for 15 minutes.

Anti-biofouling test: The anti-biofouling test was conducted in a transparent glass tank with a dimension

of 45 × 45 × 45 cm^3 simulating a natural marine ecosystem. The tank was filled with 91 L of simulated seawater (salinity of 33 g/L), and water circulation was maintained using an Eheim 250 filter. To establish a marine-simulative environment, several artificially cultivated coral stones, sourced from the local market in Shenzhen, were introduced into the tank. Subsequently, 10 mL of high-purity commercial nitrifying bacteria was added to function as decomposers and accelerate the nitrogen cycle within the system. Simultaneously, a 100 W light-emitting diode lamp emitting visible light in the wavelength range of 400–750 nm was employed to provide the illumination necessary for microorganism growth and biofilm formation. After allowing the aquatic environment to stabilize for 7 days, T-PAC and CB-C_2S samples (50 × 50 × 10 mm^3) were placed on the top of the coral stones and subjected to daily light emission diode irradiation for 8 hours over a period of 30 days, to accelerate the biofouling process. The anti-biofouling performance was assessed by comparing the surface biofouling coverage as well as the optical properties of the T-PAC and CB-C_2S samples before and after the biofouling test.

Figure R10a demonstrates the initial surface conditions of T-PAC and CB-C_2S in a submerged environment. Both samples appear white from the front view. However, in a side-view observation, the superhydrophobic T-PAC exhibits a mirror-like reflection, which is a result of the entrapped air layer (air cushion) on its surface. As shown in Figure R10b, after 30 days of immersion in an aquatic environment, the T-PAC maintained a clean and white surface, whereas the CB-C_2S surface was extensively covered by yellowish biofouling, reaching approximately 75% of its area (Figure R10c). This marked difference is attributed to the entrapped air layer on the submerged T-PAC surface, which forms an air/water interface that effectively inhibits the contact and subsequent adhesion of microorganisms—preventing anchoring via pili and other cellular mechanisms typically employed on solid surfaces[18]. Consequently, the T-PAC retained a $\bar{R}_{solar}$ value close to the initial level, which is in striking contrast to the CB-C_2S remarkably losing its solar reflectivity due to biofouling coverage that blocks light/CB-C_2S interaction (Figure R10d).

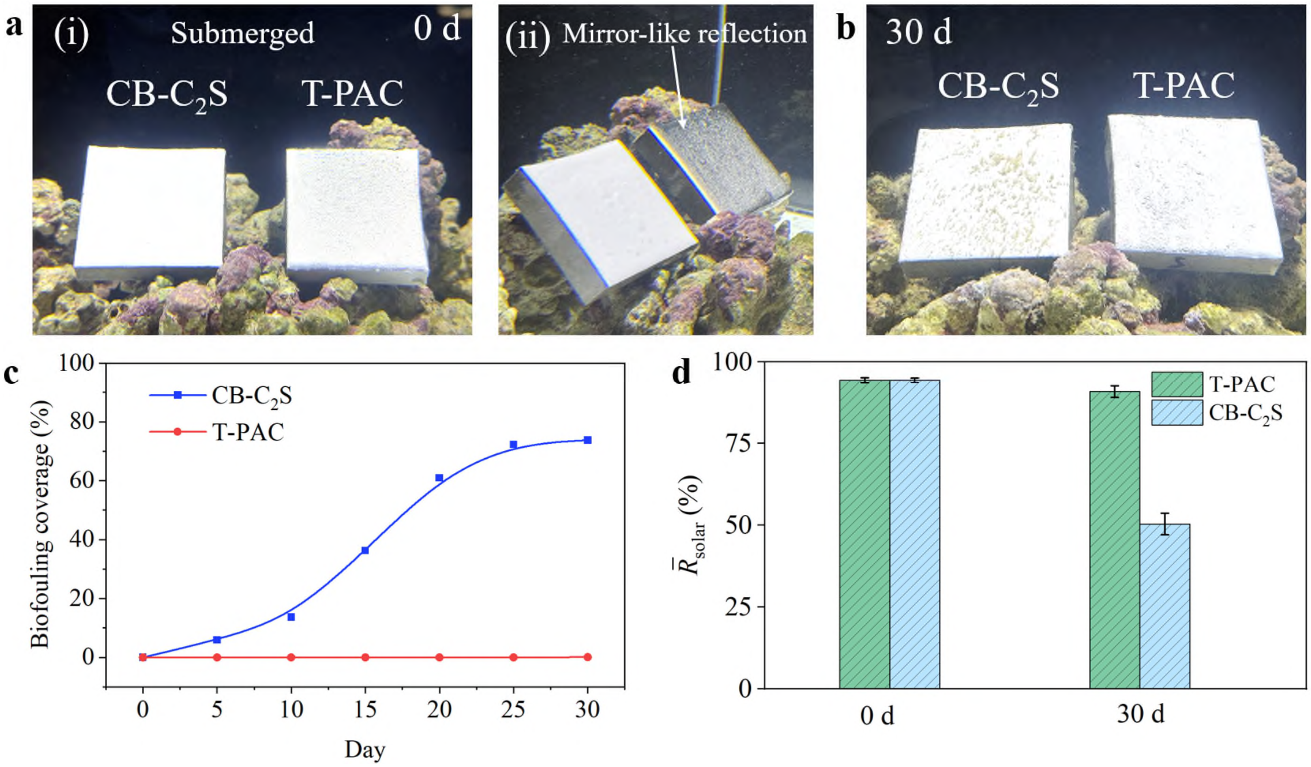

Figure R10. a. Initial surface conditions of T-PAC and CB-C_2S in a submerged environment. **b**. Surface

morphology of T-PAC and CB-C_2S after 30 days of simulated seawater immersion. **c**. Biofouling coverage of T-PAC and CB-C_2S in response to different immersion durations. **d**. Measured $\bar{R}_{solar}$ of T-PAC and CB-C_2S before and after 30 days of seawater immersion.

7. Passive cooling coating is expected to exhibit dual effects both for energy conservation and also the corrosion inhibition in marine environment in this work. Comparison with that of standard marine coatings (e.g., epoxy, polyurethane, silane treatments) is of great significance. Include a brief discussion on the scalability, application cost, repairability, and limitations of the coating system, which are crucial for assessing its practical translational potential. Actually, passive cooling coatings are suitable for hot climate environments, while superhydrophobic coatings are not suitable for long-term humid or underwater environments.

Response: In response to the reviewer's suggestion, we have added a systematic comparative analysis between our T-PAC coating and established marine protection strategies. This addition clarifies T-PAC's multifunctional paradigm, which synergistically integrates passive radiative cooling with a superhydrophobic barrier—moving beyond single-mechanism approaches. Moreover, we have elaborated on its practical advantages, including excellent UV resistance, cementitious compatibility, sustainability of its carbonated matrix, a low preliminary cost (~\$2.71/m^2), and excellent repairability. Furthermore, we deepened the discussion on the mutual reinforcement between the cooling and anti-wetting layers, justifying its effectiveness in high-humid splash zone environments. These revisions underscore T-PAC's potential as a comprehensive and energy-free solution, which are detailed in following:

Table R1. Comparing T-PAC with established coating systems for marine concrete protection.

Coating system	Protection Mechanism	Key Advantages	Key Limitations/Drawbacks
**This work: T-PAC**	**1. Passive Radiative Cooling:** Reduces surface temperature, suppressing Arrhenius kinetics. **2. Superhydrophobic Barrier:** Blocks chloride-laden water ingress. **3. Enhanced Bonding:** Silica gel ensures integral bonding.	**Multifunctional:** Simultaneously mitigates thermal and chemical corrosion drivers. **Energy-free & Passive:** Requires zero operational energy. **Chemically Compatible:** Seamless integration with concrete substrate. **Carbon-Negative Process:** Carbonation sequesters CO_2. **Durable Adhesion:** Cementitious bonding eliminates delamination risk.	**Application Specific:** Optimal for hot, solar-exposed climates.
**Epoxy / Polyurethane**	**1. Physical Barrier:** Dense film isolates substrate.	**Proven Technology:** Widely available and specified. **High Mechanical Strength:** Excellent abrasion resistance.	**Single-Function:** Only a chloride barrier; ignores thermal acceleration. **Poor UV/Heat Resistance:** Prone to chalking, yellowing, and degradation. **Adhesion Failure:** Thermo-mechanical mismatch with concrete causes delamination. **Environmental Concern:** Derived from petrochemicals.
**Silane Treatments**	**1. Pore Linings:** Hydrophobizes capillary pores.	**Deep Penetration:** Treats the substrate bulk. **Vapor Permeable**	**Single-Function:** Only a water repellent; no thermal regulation. **Durability Concerns:** Limited service life;
**Cathodic Protection**	**1. Electrochemical Intervention:** Forces steel to be cathodic.	**Highly Effective:** Can stop ongoing corrosion.	**Energy-Intensive:** Requires continuous power supply and monitoring. **High Maintenance:** Complex and costly over a full lifecycle. **Not a Preventive Coating:** An active system, not a passive material solution.

As detailed in table R1, our T-PAC coating establishes a new paradigm by moving beyond single-mechanism protection. Particularly, compared to pore-lining treatments such as silanes, which provide only hydrophobic functionality, our coating introduces a crucial second protective dimension: passive radiative cooling. This dual-functionality simultaneously suppresses the Arrhenius-type kinetic acceleration of corrosion (Figure 5h) by maintaining sub-ambient temperatures while also providing an excellent barrier against capillary seawater absorption (Figure 3g, f). This positions the T-PAC system as a comprehensive solution to the coupled thermo-chemo degradation inherent to marine environments. Additionally, unlike epoxy/polyurethane coatings, which are susceptible to UV degradation[19] and delamination due to their polymeric nature and thermomechanical mismatch with concrete[20], the T-PAC's inorganic, cementitious matrix forms a compatible and robust interface (Figure 2g), ensuring long-term durability. Moreover, our approach provides a passive, zero-energy alternative to active systems like cathodic protection, eliminating the need for continuous energy input and sophisticated monitoring. This makes it particularly suitable for large-scale, remote marine infrastructure where maintenance and energy costs are prohibitive. Another crucial advantage of T-PAC lies in its sustainability, in which the use of carbonation-activated C_2S as a binder is a carbon-negative process, aligning with global decarbonization goals, whereas the production of organic coatings like epoxy carries a significant carbon footprint and petrochemicals (Page 8, Lines 251-253). Thus, the T-PAC coating is a comprehensive solution that synergistically integrates multiple protective functions—thermal regulation, Cl^--blocking, and enhanced bonding—into a single, sustainable, energy-free anti-corrosion system, offering a comprehensive solution for enhancing the durability of marine concrete structures under coupled thermo-chemo environmental stress.

Following the reviewer's suggestion, we have added dedicated content in the Discussion section addressing the scalability (Page 8, Lines 248–250), application cost (Page 8, Lines 250–251), repairability (Page 15, Lines 429–430), and limitations (Pages 24-25, Lines 760–763) of the T-PAC. Regarding scalability, T-PAC coating demonstrates strong potential for large-scale deployment, as it employs widely available, mature, low-cost raw materials (C_2S, $BaSO_4$, and SiO_2) and can be applied via simple, scalable methods such as slurry brushing and spray-coating. These processes align with conventional construction practices and do not require sophisticated equipment. A preliminary cost estimate, based on typical prices of key raw materials (C_2S at \$4.93 kg^{-1}, $BaSO_4$ NPs at ≈\$0.17 kg^{-1}, hydrophobic SiO_2 NPs at \$4.2 kg^{-1}), indicates a material cost of approximately \$2.71/m^2 (Supplementary Note 3), which is highly competitive against commercial marine coatings like epoxy (\$28.9/m^2) and silane treatments (\$4.38/m^2). Although C_2S is currently the primary cost driver, its price is expected to decrease significantly with mass production using established synthetic routes referenced in previous studies[21-23]. Furthermore, the cementitious nature of T-PAC endows it with excellent repairability (Figure R8). Localized damage can be efficiently restored by reapplying the $C_2S/BaSO_4$ layer or the hydrophobic top layer. This spot-repair capability offers a distinct advantage over many polymer-based coatings, which often require large-scale removal and reapplication to ensure adequate adhesion. We also noted that the T-PAC coating system has certain limitations. In particular, its performance performs optimally in hot, sunny climates, where radiative cooling effects are most pronounced, but can be compromised by cloudy conditions due to its reliance on radiative heat dissipation. Nonetheless, T-PAC presents a compelling, energy-free, and multifunctional solution for the vast number of marine structures where thermal loading and chloride ingress are the predominant degradation mechanisms.

We agree with the reviewer that traditional radiative coolers perform optimally in hot, dry climates, while

superhydrophobic surfaces face durability issues in submerged environments. However, our coating targets the splash and atmospheric zones of marine structures—the most corrosion-vulnerable areas—where surfaces undergo repeated cycles of wetting (from spray) and drying (driven by solar and wind exposure). Despite humid atmospheres can reduce radiative cooling efficacy, our coating maintains sub-ambient temperature drop of ~2.95 °C (Figure R11) in elevated humidity (~77.4%) due to optimized radiative cooling-wetting design. Furthermore, the core innovation of our work addresses this challenge by integrating both functionalities into a composite system that exhibits mutually enhanced robustness and applicability—surpassing what either approach could achieve alone. As illustrated in Figure R1, the superhydrophobic top layer preserves radiative cooling performance via preventing seawater absorption or fouling adhesion, thus ensuring sustained high solar reflectance and unobstructed mid-infrared thermal emissions to the sky. In return, the integrated radiative cooling function enhances anti-wetting performance by promoting rapid drying through sub-ambient cooling, since it thermally stabilizes the three-phase contact line of seawater during dry intervals, which reduces surface condensation and facilitates the recovery of water repellency. In essence, the two functionalities are not simply additive but operate in a mutually reinforcing and protective manner. This synergy renders the T-PAC uniquely suited for real marine applications, overcoming the inherent limitations of each technology when used independently. We have incorporated this discussion into the revised manuscript (Pages 24-25, Lines 747-777) to better articulate this conceptual advance.

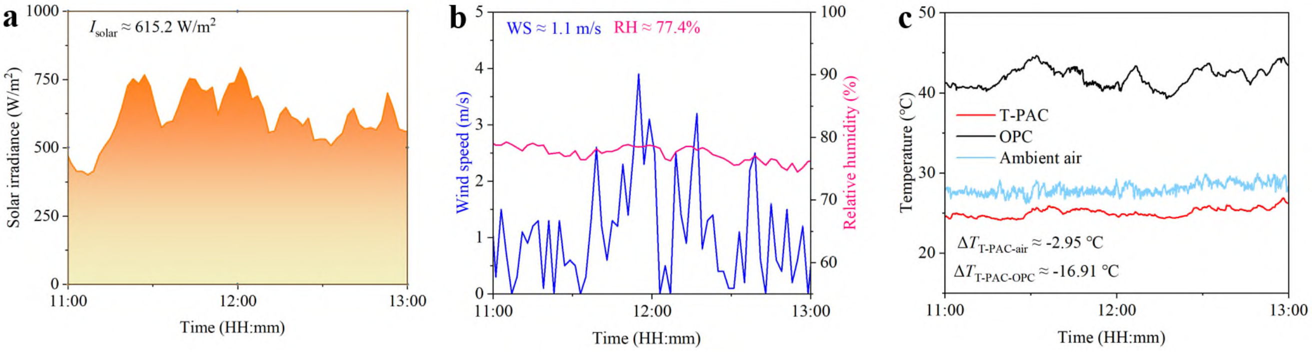

Figure R11. **Outdoor cooling performance of the T-PAC under a high relative humidity of 77.4%. a** Solar irradiance recorded during the hottest period. **b** Wind speed and relative humidity during the outdoor measurement. **c** Temperature variation of T-PAC, OPC, and ambient air during the tested period.

8. The statement "During the heat transfer equilibrium, heat gain from the solar irradiation causes instability of passive films on steel rebars due to reduced critical chloride threshold required for depassivation under elevated temperatures" in line 129 is not logical for the knowledge of chloride induced corrosion. In Figure S1, if available, the ambient temperature in Shenzhen should be added.

Response: The reviewer's comments regarding the clarity of expression and the necessity of including ambient temperature data for Shenzhen have been addressed in the revised manuscript.

Specifically, we have corrected it to "During the heat transfer equilibrium, heat gain from solar irradiation elevates the temperature of concrete-steel system, which significantly accelerates the transport of chlorides to the steel surface, thereby promoting earlier depassivation and corrosion initiation[24]". Please see the revision in the revised manuscript (Page 4, Lines 143-145).

Following the reviewer's suggestion, we have added the ambient temperature data of Shenzhen (Nanshan

District) on the same day to Figure S1. The updated figure clearly shows the temperature difference between the concrete surface and the ambient environment, underscoring the significance of thermal regulation.

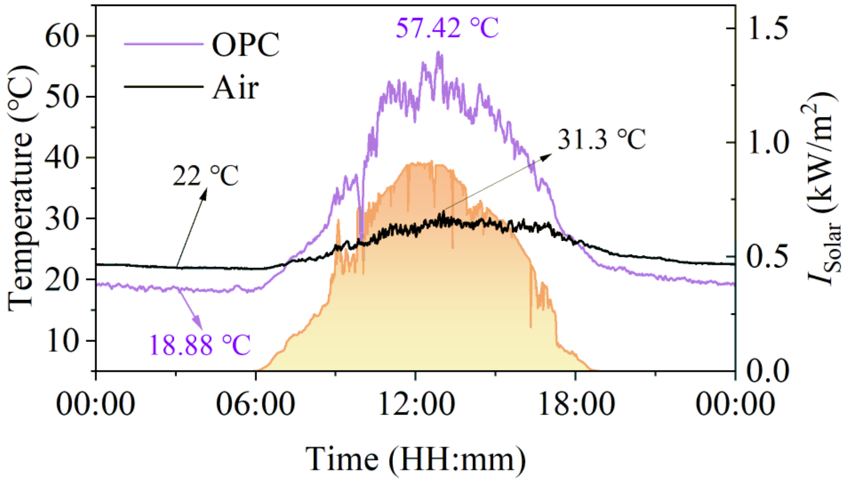

Figure R12. Time-dependent temperature variation of ordinary Portland cement and ambient air under direct sunlight irradiation.

In summary, we tried our best to improve the manuscript and made some changes with remarks in the revised manuscript. We appreciate for the Reviewer, kind comments and questions and hope that the reply will meet your approval. Finally, we would like to express our great appreciation for your valuable suggestions.

Reviewer #2 (Remarks to the Author):

This manuscript presents a novel and interesting study on a multifunctional coating system that innovatively addresses the coupled thermo-wetting degradation mechanisms in marine concrete structures. The authors demonstrate remarkable insight by integrating radiative cooling physics with superhydrophobic materials science to develop a dual-barrier coating (T-PAC) that simultaneously suppresses both Arrhenius-type kinetic acceleration and chloride ion penetration. The work is scientifically rigorous, featuring multi-scale characterization—spanning spectral analysis, wetting/thermal performance validation under simulated environmental conditions, electrochemical corrosion monitoring and sophisticated interpretation of results through coupled heat-mass transfer modelling. The coating's passive operation, cementitious compatibility, and scalability further enhance its practical relevance. While the study makes a significant contribution to the field, a few minor revisions could further strengthen its clarity and impact.

Response: We are grateful for the reviewer's high comments on our research.

This paper is recommended for publication in Nature Communications after addressing the following comments:

1. Except for C_2S, C_3S also exhibits carbonation reactivity and chemical compatibility with concrete surface, why do the authors choose C_2S as the binder instead of C_3S? A comparative discussion on potential benefits would provide valuable insights into this choice.

Response: We appreciate the reviewer's interest in the comparison between C_2S and C_3S. We have added comparative discussion in the revised manuscript (Page 7, Lines 203-204). Specifically, compared

to C_3S, C_2S has the following advantages: (1) Slower hydration and higher carbonation reactivity allows better carbonation control[25]; (2) Lower Fe_2O_3 content (0.2% vs 3.1% in C_3S) reduces solar absorption (Figure R1).

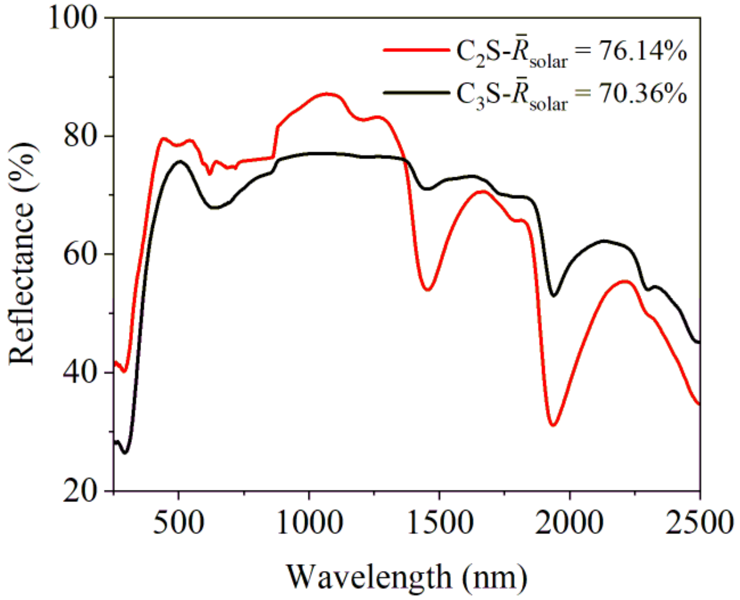

Figure R1 Solar reflectance measurement of C_2S and C_3S.

2. The abbreviation "T-PAC" (temperature-wetting mediated passive anti-corrosion coating) is introduced in both the Introduction and Results sections; however, terms such as "thermal-wetting" or "thermo-wetting" are inconsistently used elsewhere in the manuscript. Please use consistent description for better reader accessibility.

Response: We have standardized all terminology to "temperature-wetting" throughout the manuscript and defined T-PAC only once in the Results section (Page 4, Line 126) for consistency.

3. Reasons for choosing $BaSO_4$ NPs over other dielectric particles (e.g., Al_2O_3, TiO_2, $CaCO_3$, or *h*-BN) as the functional filler for improving solar reflectance and mid-infrared emittance should be provided.

Response: In our research, $BaSO_4$ NPs were selected as the radiative cooling filler due to their balanced optical, stability, and cost advantages. Their wide bandgap (~7.6 eV) and suitable refractive index enable strong solar scattering, while SO_4^{2-} phonons provide high emissivity in the atmospheric window (8 - 13 μm). Compared to alternatives like Al_2O_3, which can dissolve in cement, $BaSO_4$ remains chemically stable in alkaline environments. Furthermore, it is significantly more cost-effective (~$0.17/kg) than high-performance alternatives such as *h*-BN ($135.4/kg), supporting scalable application. These points have been elaborated in the revised manuscript (Page 27, Lines 827-828).

4. In line 73, the authors refer to "high solar reflectance (0.3~2.5 μm)", but, as a rule, spectral measurements are carried out in the range of 0.28~2.5 μm. The authors should unify the wavelength range of the solar band throughout the main text.

Response: We have corrected the solar spectrum to "0.28–2.5 μm" throughout the manuscript (Page 3, Line 76; Page 7, Line 222; etc.).

5. In line 75, the term “coastal concrete” is used, but elsewhere in the manuscript, “coastal concrete structure” appears to be the intended term. Please check the manuscript for the same problem.

Response: We have standardized to “coastal concrete structures” throughout the text, which can be found in Page 3, Line 78; Page 13, Line 378; etc.

6. Lines 422-423: In the outdoor test, the authors note that the outdoor test was conducted without a wind shelter, which differs from methodologies used in other studies. Please elaborate on this choice.

Response: The wind-open configuration in our research was intentionally chosen to simulate real marine exposure conditions where convective heat transfer significantly influences cooling performance. While wind shelters maximize laboratory-measured cooling power, they overestimate real-world performance, our approach provides conservative, practically relevant cooling data. This clarification is now included in the revised manuscript (Page 16, Line 470).

References

1 Ashraf, W. & Olek, J. Carbonation activated binders from pure calcium silicates: Reaction kinetics and performance controlling factors. *Cem. Concr. Compos.* **93**, 85-98 (2018).

2 Hossain, F. M., Murch, G. E., Belova, I. V. & Turner, B. D. Electronic, optical and bonding properties of $CaCO_3$ calcite. *Solid State Commun.* **149**, 1201-1203 (2009).

3 Peng, L., Shen, P., Poon, CS., Zhao, Y. & Wang, F. Development of carbon capture coating to improve the durability of concrete structures. *Cem. Concr. Res.* **168**, 107154 (2023).

4 Diamanti, M. V. *et al.* Effect of polymer modified cementitious coatings on water and chloride permeability in concrete. *Constr. Build. Mater.* **49**, 720-728 (2013).

5 Shafei, B., Alipour, A. & Shinozuka, M. Prediction of corrosion initiation in reinforced concrete members subjected to environmental stressors: A finite-element framework. *Cem. Concr. Res.* **42**, 365-376 (2012).

6 Page, C. L., Short, N. R. & El Tarras, A. Diffusion of chloride ions in hardened cement pastes. *Cem. Concr. Res.* **11**, 395-406 (1981).

7 Collepardi, M., Marcialis, A. & Turriziani, R. Penetration of chloride ions into cement pastes and concretes. *J. Am. Ceram. Soc.* **55**, 534-535 (1972).

8 Alhozaimy, A. M., Ahmed, M., Hussain, R. R. & Al Negheimish, A. Quantitative non-linear effect of high ambient temperature on chloride threshold value for steel reinforcement corrosion in concrete under extreme boundary conditions. *Materials* **14**, 7595 (2021).

9 Rothman, K. J. Synergy and antagonism in cause-effect relationships. *AM. J. Epidemiol.* **99**, 385-388 (1974).

10 Farahani, A., Taghaddos, H. & Shekarchi, M. Prediction of long-term chloride diffusion in silica fume concrete in a marine environment. *Cem. Concr. Compos.* **59**, 10-17 (2015).

11 Alhozaimy, A., Hussain, R. R., Al Zaid, R. & Al Negheimish, A. Coupled effect of ambient high relative humidity and varying temperature marine environment on corrosion of reinforced concrete. *Constr. Build. Mater.* **28**, 670-679 (2012).

12 Wu, J., Li, H., Wang, Z. & Liu, J. Transport model of chloride ions in concrete under loads and drying-wetting cycles. *Constr. Build. Mater.* **112**, 733-738 (2016).

13 Zuquan, J., Xia, Z., Tiejun, Z. & Jianqing, L. Chloride ions transportation behavior and binding capacity of concrete exposed to different marine corrosion zones. *Constr. Build. Mater.* **177**, 170-183 (2018).

14 Ting, M. Z. Y., Wong, K. S., Rahman, M. E. & Meheron, S. J. Deterioration of marine concrete exposed to wetting-drying action. *J. Clean. Prod.* **278**, 123383 (2021).

15 De Weerdt, K., Wilson, W., Machner, A. & Georget, F. Chloride profiles – What do they tell us and how should they be used? *Cem. Concr. Res.* **173**, 107287 (2023).

16 Melchers, R. E. & Li, C. Q. Reinforcement corrosion initiation and activation times in concrete structures

exposed to severe marine environments. *Cem. Concr. Res.* **39**, 1068-1076 (2009).

17 Chen, C. *et al.* Prediction model for equivalent exposure time in indoor dry-wet cycling tests for concrete in marine tidal zones. *Constr. Build. Mater.* **451**, 138778 (2024).

18 Epstein, A. K., Wong, T. S., Belisle, R. A., Boggs, E. M. & Aizenberg, J. Liquid-infused structured surfaces with exceptional anti-biofouling performance. *Proc. Natl. Acad. Sci.* **109**, 13182-13187 (2012).

19 Zhang, X. Metamaterials for perpetual cooling at large scales. *Science* **355**, 1023-1024 (2017).

20 Yan, X., Yang, M., Duan, W. & Cui, H. Particle–solid transition architecture for efficient passive building cooling. *ACS Nano* **18**, 27752-27763 (2024).

21 Chen, J., Zhao, M., Liu, Z., Wang, F. & Hu, S. Bioinspired in situ synthesis of high-strength bulk CO_2 mineralized ceramics at room temperature. *ACS Nano* **19**, 1732-1742 (2025).

22 Chen, J., Liu, Z., Zhang, S., Hu, S. & Wang, F. Energy-efficient fabrication of biomimetic materials for sustainable infrastructure applications. *Adv. Sci.* **12**, e03854 (2025).

23 Zhao, S., Liu, Z., Wang, F., Hu, S. & Liu, C. Effect of extended carbonation curing on the properties of γ-C_2S compacts and its implications on the multi-step reaction mechanism. *ACS Sustain. Chem. Eng.* **9**, 6673-6684 (2021).

24 Hussain, S. E., Rasheeduzzafar, Al Musallam, A. & Al Gahtani, A. S. Factors affecting threshold chloride for reinforcement corrosion in concrete. *Cem. Concr. Res.* **25**, 1543-1555 (1995).

25 Mu, Y., Liu, Z. & Wang, F. Comparative study on the carbonation-activated calcium silicates as sustainable binders: reactivity, mechanical performance, and microstructure. *ACS Sustain. Chem. Eng.* **7**, 7058-7070 (2019).
